# Structural and Functional Analysis of Peptides Derived from KEX2-Processed Repeat Proteins in Agaricomycetes Using Reverse Genetics and Peptidomics

**DOI:** 10.1128/spectrum.02021-22

**Published:** 2022-10-31

**Authors:** Eva Vogt, Lukas Sonderegger, Ying-Yu Chen, Tina Segessemann, Markus Künzler

**Affiliations:** a ETH Zürich, Department of Biology, Institute of Microbiology, Zürich, Switzerland; University of Guelph

**Keywords:** dikaritins, KEX2, KEX2-processed repeat proteins, peptides, RiPPs, STE13, mycology

## Abstract

Bioactivities of fungal peptides are of interest for basic research and therapeutic drug development. Some of these peptides are derived from “KEX2-processed repeat proteins” (KEPs), a recently defined class of precursor proteins that contain multiple peptide cores flanked by KEX2 protease cleavage sites. Genome mining has revealed that KEPs are widespread in the fungal kingdom. Their functions are largely unknown. Here, we present the first in-depth structural and functional analysis of KEPs in a basidiomycete. We bioinformatically identified KEP-encoding genes in the genome of the model agaricomycete Coprinopsis cinerea and established a detection protocol for the derived peptides by overexpressing the C. cinerea KEPs in the yeast Pichia pastoris. Using this protocol, which includes peptide extraction and mass spectrometry with data analysis using the search engine Mascot, we confirmed the presence of several KEP-derived peptides in C. cinerea, as well as in the edible mushrooms Lentinula edodes, Pleurotus ostreatus, and Pleurotus eryngii. While CRISPR-mediated knockout of C. cinerea *kep* genes did not result in any detectable phenotype, knockout of *kex* genes caused defects in mycelial growth and fruiting body formation. These results suggest that KEP-derived peptides may play a role in the interaction of C. cinerea with the biotic environment and that the KEP-processing KEX proteases target a variety of substrates in agaricomycetes, including some important for mycelial growth and differentiation.

**IMPORTANCE** Two recent bioinformatics studies have demonstrated that KEX2-processed repeat proteins are widespread in the fungal kingdom. However, despite the prevalence of KEPs in fungal genomes, only few KEP-derived peptides have been detected and studied so far. Here, we present a protocol for the extraction and structural characterization of KEP-derived peptides from fungal culture supernatants and tissues. The protocol was successfully used to detect several linear and minimally modified KEP-derived peptides in the agaricomycetes C. cinerea, L. edodes, P. ostreatus, and P. eryngii. Our study establishes a new protocol for the targeted search of KEP-derived peptides in fungi, which will hopefully lead to the discovery of more of these interesting fungal peptides and allow a further characterization of KEPs.

## INTRODUCTION

Fungi produce a wide range of bioactive natural products, including peptides. Some peptides have been shown to be of immense value as therapeutics. For example, the use of the non-ribosomal peptides, penicillin, and cyclosporine as antibiotics and immunosuppressants, respectively, has revolutionized modern medicine (1). While the synthesis of non-ribosomal peptides (NRPs) relies on large modular enzymes called non-ribosomal peptide synthases (NRPSs) (2), the sequences of “ribosomally synthesized and posttranslationally modified peptides” (RiPPs) are genetically encoded as parts of precursor proteins. Residues in the core peptide region undergo posttranslational modifications, e.g., cyclization, acetylation, glycosylation, epimerization, and methylation, followed by release of the core peptide from the precursor protein by proteolytic cleavage (3).

**FIG 1:**
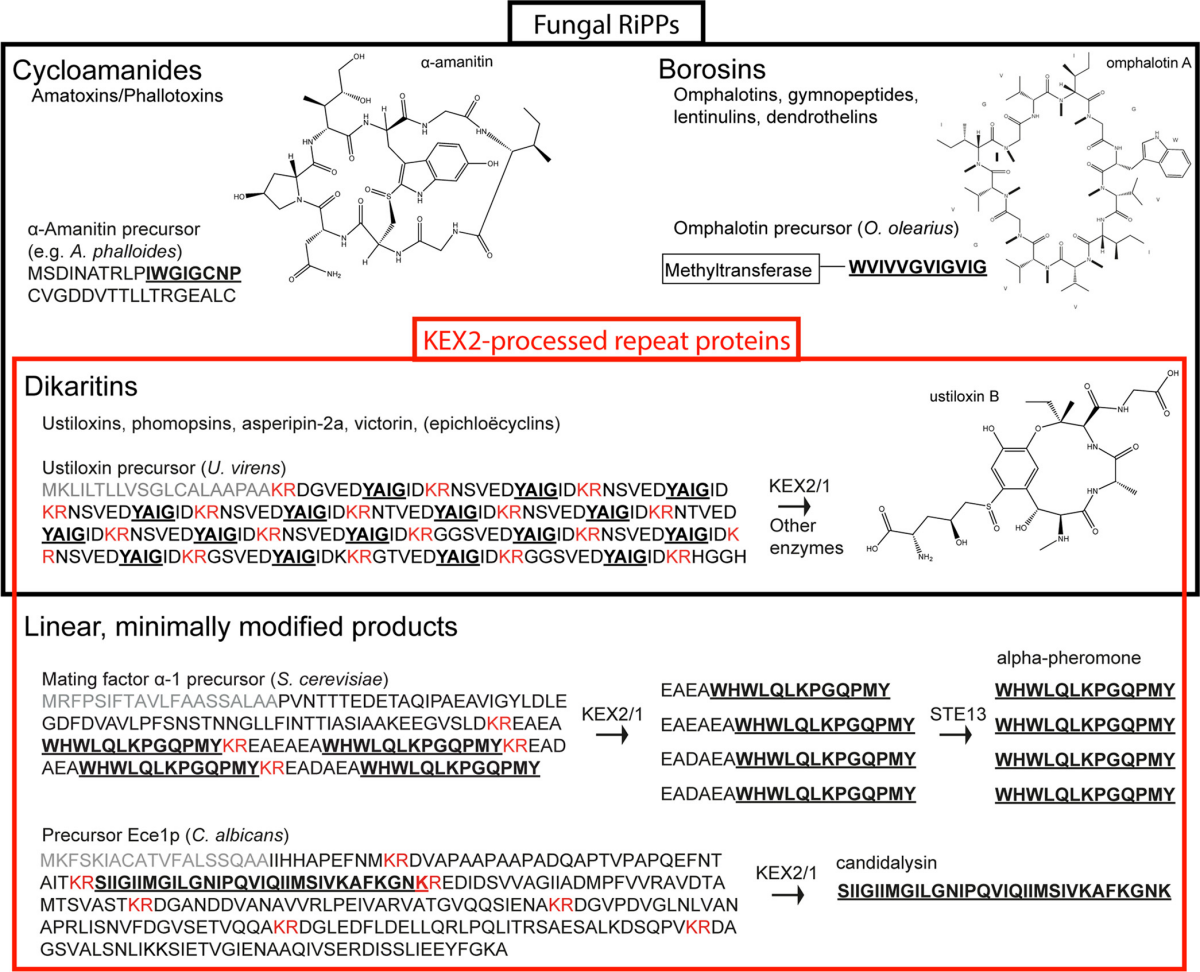
Illustration of the 3 major classes of fungal “ribosomally synthesized and post-translationally modified peptides” (RiPPs, black box) and their overlap with peptides derived from KEX2-processed repeat proteins (KEPs, red box). Fungal RiPPs include cycloamanides (7) and borosins (8, 65, 66), as well as dikaritins (10, 12–14). The dikaritin precursors possess a KEP-like organization with a signal peptide for secretion (labeled in gray) and repetitive units of core peptides that are flanked by dibasic KEX2 cleavage sites (labeled in red). The dibasic residues are cleaved by endoproteinase KEX2 and carboxypeptidase KEX1, and the resulting peptides are further modified and cyclized. According to the current consensus, the cyclic, modified peptides of the dikaritins are considered RiPPs (e.g., ustiloxin B) (9, 11), whereas the linear, minimally modified peptides of other KEPs are not. These KEP-derived peptides include, for example, the α-pheromones of ascomycetes, in which the dipeptides XP/XA are removed by the dipeptidyl aminopeptidase STE13 (20), and candidalysin of C. albicans (17).

To date, only a few RiPP classes from fungi have been discovered (Fig. 1) (4, 5). The precursors of the 2 RiPP classes cycloamanides and borosins are processed in the cytoplasm by oligopeptidases of the S9 protease family (6–8), whereas processing of dikaritins, including ustiloxins, phomopsins, asperipin-2a, victorin, and possibly epichloëcyclins, is mediated by the Golgi-localized kexin endoproteinase KEX2 (9–14). The dikaritin precursors contain an N-terminal signal peptide for secretion and multiple repeats of core peptides separated by dibasic residues “KR,” “RR,” or “KK” that serve as recognition and cleavage sites for the KEX2 endoproteinase. The signal peptide directs the protein to the secretory pathway and is removed by the signal peptidase upon translocation into the lumen of the endoplasmic reticulum (ER). KEX2 is localized in the late Golgi and cleaves the precursor protein at the C-terminal end of the dibasic residue motifs. Subsequently, the C-terminal basic residues are removed from the peptides by the Golgi-localized exopeptidase KEX1 and the peptides are secreted after additional modification in the Golgi (Fig. 1) (4).

Recently, bioinformatic studies by Le Marquer et al. 2019 and Umemura 2020 showed that precursor proteins with a dikaritin-like architecture are widely distributed in all fungal phyla (15, 16). These proteins, which contain an N-terminal signal sequence for secretion and repeats of short peptide sequences flanked by KEX2 cleavage sites, were termed “KEX2-processed repeat proteins” (KEPs). Well-characterized representatives of KEP-derived peptides are the aforementioned dikaritins. However, while the dikaritins are extensively posttranslationally modified and therefore considered RiPPs, this does not seem to be the case for many other KEP-derived peptides. The following KEP-derived peptides are linear with no or at most one additional processing step after KEX2/1 cleavage: The cytolytic candidalysin is produced by the opportunistic pathogen Candida albicans and is crucial for mucosal infection (Fig. 1) (17), while peptides from the precursor protein Rep1 in Ustilago maydis form amyloid-like fibrils (18, 19). The best studied KEP-derived peptides are the α-pheromones of ascomycetes, e.g., Saccharomyces cerevisiae, as shown in Fig. 1. The α-pheromone precursor contains an STE13 recognition motif, a repetitive dipeptide sequence of XP or XA, where X is often aspartic acid or glutamic acid, located after the KEX2 cleavage site and removed by the dipeptidyl aminopeptidase STE13 after KEX2/1 cleavage (20, 21).

Most KEPs encode peptides of unknown function. Many ascomycetous KEPs likely represent precursors of α-type mating pheromones, based on the presence of STE13 recognition motifs. Interestingly, the same precursor architecture is also found in many KEPs from basidiomycetes. This is surprising because basidiomycetous mating pheromones are of the a-type which are synthesized in the cytoplasm, prenylated, and secreted via ABC transporters (22, 23). Thus, the identification of α-type pheromone precursors in basidiomycetes was unexpected and raises the question for the function of these peptides.

In the past, fungal RiPPs and KEP-derived peptides were mostly studied using forward genetics, where the peptides were first isolated and characterized and only then the corresponding precursor genes were identified (e.g., ustiloxins, phomopsins, candidalysin, Rep1, omphalotin). In this paper, we established a protocol for the identification of KEP-derived peptides in fungal samples by a combination of reverse genetics and peptidomics. The protocol was developed using the model agaricomycete Coprinopsis cinerea, but is applicable to other fungi. In a first step, we bioinformatically screened the predicted proteome of C. cinerea for KEP-encoding genes. Second, we expressed 6 different KEP genes from C. cinerea in the yeast Pichia pastoris to, (i) establish a protocol for extraction and detection of KEP-derived peptides by mass spectrometry in P. pastoris culture supernatants known for their low complexity in terms of proteins and peptides (24); (ii) obtain an indication that the detected KEPs were indeed processed to peptides, and (iii) use the structures of the detected peptides as a reference as to which peptides to expect in C. cinerea in terms of peptide length and peptide modifications. In a third step, we applied the established peptide extraction and detection protocols to detect KEP-derived peptides in culture supernatants and tissue samples of the agaricomycetes C. cinerea, Lentinula edodes, Pleurotus ostreatus, and Pleurotus eryngii. Finally, we examined the phenotypes of C. cinerea *kep* and *kex* knockout strains regarding mycelial growth and fruiting body formation, and tested synthetic KEP-derived peptides for growth inhibition of bacteria. The results of these analyses suggest that individual KEX proteases are redundant in the processing of KEPs but are required for normal mycelial growth and fruiting body formation. Our results did not reveal any specific function for any of the KEP-derived peptides examined.

## RESULTS

### Bioinformatic analysis predicts 22 KEX2-processed repeat proteins (KEPs) in C. cinerea.

We screened the predicted proteome of C. cinerea AmutBmut available on JGI Mycocosm (25, 26) for proteins with a KEP-like organization. We used 2 different methods: In method 1, all proteins were cleaved *in silico* at putative KEX2 cleavage sites, and the resulting fragments of each protein were aligned with BLAST+ (27, 28). Proteins lacking a signal sequence for secretion were excluded. Method 2 used the tool RADAR (29) to highlight repeats in signal peptide containing proteins shorter than 300 amino acids, which were then visually inspected.

The first method using BLAST+ yielded 19 putative C. cinerea KEPs. The results of method 2 largely overlapped with the ones of method 1, with 2 additional hits. Additionally, 1 protein that had been missed by both methods was manually detected by chance and added to the list of putative KEPs. Of these total 22 hits, 2 were previously detected by both Le Marquer et al. 2019 and Umemura 2020, and 8 were detected only by Umemura 2020. Additionally, Le Marquer et al. and Umemura had found 2 and 7 proteins, respectively, that were not detected in this study, resulting in a total number of 31 putative C. cinerea KEPs (Table S1). In comparison, the KEP screening method of Le Marquer et al. used a similar approach to ours, i.e., proteins with signal sequences were cut *in silico* at KEX2 cleavage sites and a sequence comparison was performed with the resulting peptide fragments (15). The main difference is the use of the tool FIMO (30) to determine sequence similarity instead of BLAST+. Umemura's approach differed slightly in that this author first screened the genome for proteins with signal sequences and sequence repeats using an in-house script, before checking for the presence of KEX2 cleavage sites.

### Heterologous expression of KEPs from C. cinerea in P. pastoris and detection of the derived peptides in the culture supernatant.

**FIG 2:**
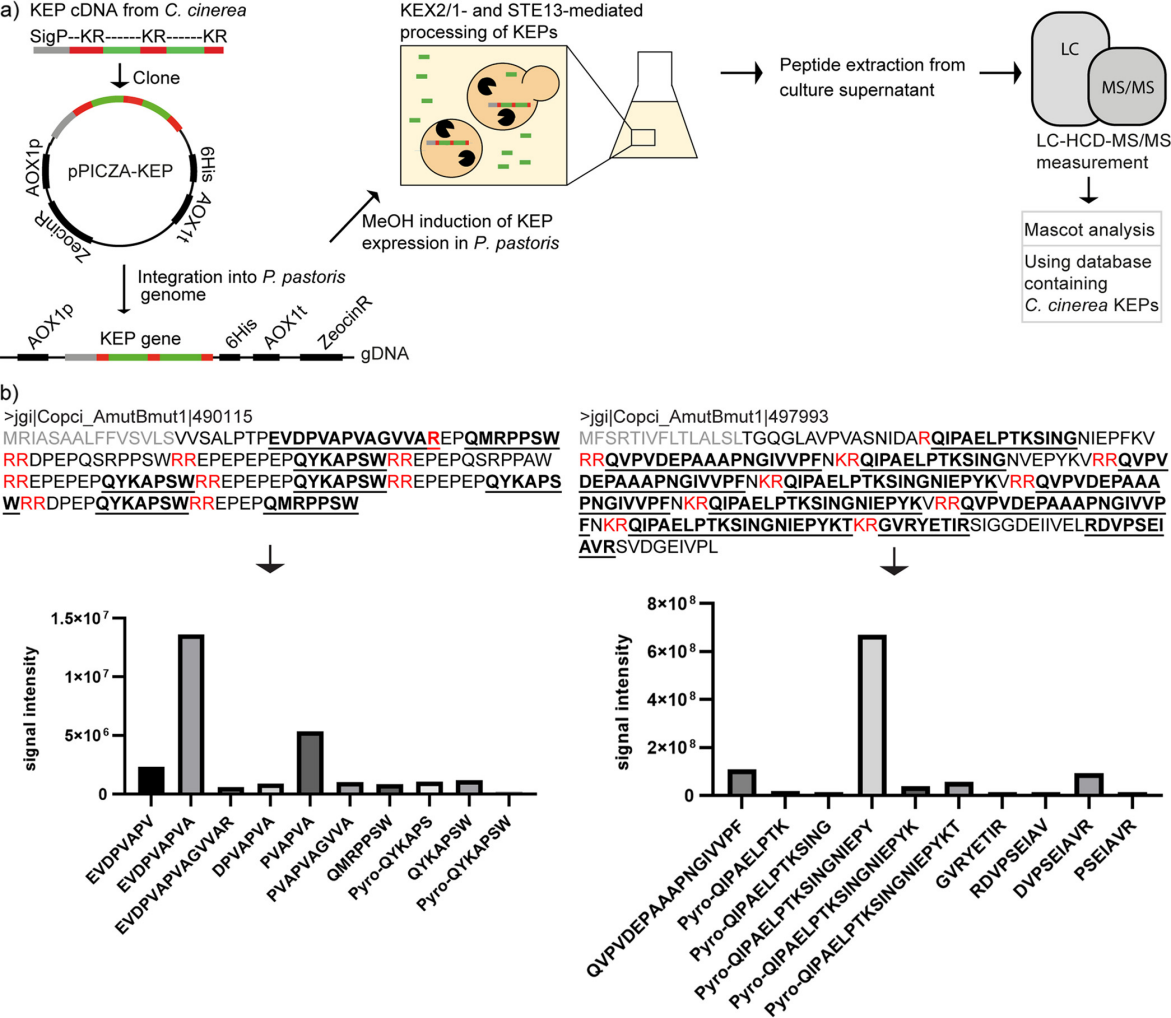
KEP-derived peptides detected in culture supernatant of Pichia pastoris after heterologous expression of KEX2-processed repeat proteins (KEPs) from Coprinopsis cinerea. (a) Workflow of the experimental procedure. KEP cDNAs from C. cinerea were cloned into P. pastoris expression vectors, integrated into the alcohol oxidase 1 (*AOX1)* locus of the P. pastoris genome and KEP production was induced by methanol. The supernatant of the liquid cultures was harvested, peptides were extracted, and the extracts measured by mass spectrometry (MS). MS/MS spectra analysis was performed using Mascot and a database of C. cinerea KEP sequences. (b) Peptides derived from expression of C. cinerea KEPs 490115 and 497993 in P. pastoris. In the respective protein sequences, the KEX2 cleavage sites are shown in red and the signal peptides in gray. The 10 peptides that were detected with the highest signal intensities in the supernatant are listed in the graph below. Their sequences are labeled in bold and underlined in the protein sequence. Many of the detected peptides contain a modification of their N-terminal glutamine residue to pyroglutamate (labeled as “pyro-Q”).

We selected 6 KEPs from C. cinerea for further investigation. All of these proteins are short and contain a high number of KEX2 cleavage sites separating peptide repeats. Previously acquired transcriptome data indicated that some of the corresponding genes were transcribed in the vegetative mycelium and fruiting bodies of C. cinerea (Fig. S1) (26, 31, 32). We expressed the C. cinerea KEPs in the yeast P. pastoris, which produced functional homologs of the endoproteinase KEX2 (Fig. S11a) (33), the carboxypeptidase KEX1 (Fig. S11c) (34), and the dipeptidyl aminopeptidase STE13 (Fig. S12a) (35). The function of P. pastoris KEX2 and KEX1 on C. cinerea proteins and peptides was demonstrated by the successful production of the KEX2-cleaved antimicrobial peptide copsin (CPP1) and its paralog CPP2 from C. cinerea in P. pastoris (31, 36). Thus, we expected that the KEPs from C. cinerea would be properly processed in this host and that the derived peptides would accumulate in the culture supernatants of the respective transformants (Fig. 2a).

Expression of the C-terminally His6-tagged C. cinerea KEPs in P. pastoris was confirmed by immunoblotting whole cell extracts of the respective transformants using anti-His6 antibodies (Fig. S2a). In some of the extracts, a distinct pattern of several smaller proteins was detectable below the intact precursor protein, possibly corresponding to KEX2 cleavage products (Fig. S2b). These bands were of equal intensity, indicating that KEX2 cleaves KEPs stochastically rather than in an N- to C- or C- to N-terminus-directed manner. Peptides were extracted from P. pastoris culture supernatant by solid phase extraction (SPE) and the extracts were analyzed by Liquid Chromatography Higher-energy Collisional Dissociation tandem Mass Spectrometry (LC-HCD-MS/MS) and the MS/MS database search tool Mascot (Fig. 2a).

We found a variety of C. cinerea KEP-derived peptides in the P. pastoris supernatants, as shown in Fig. 2b for the expression of the 2 C. cinerea KEPs 490115 and 497993 or in Fig. S3 for KEPs 405832, 434504, 503649, and 426342. Listed are the top 10 peptides detected in the P. pastoris supernatant with the highest signal intensity. Some of these peptides were modified at their N-terminal glutamine to pyroglutamate, a modification known to occur spontaneously or enzymatically and to confer increased thermal and proteolytic stability to peptides (37). Here, this modification must have occurred spontaneously as the P. pastoris genome does not encode a glutaminyl cyclase homolog. As a negative control, we confirmed that no C. cinerea KEP-derived peptides could be detected in an induced P. pastoris strain transformed with an empty PICZA vector.

The data for KEP 490115 demonstrates that P. pastoris not only successfully cleaved the KEX2 cleavage site RR, but also removed the STE13 recognition motif XA/XP from subsequent peptide products, resulting in the peptides QMRPPSW, pyro-QYKAPS, pyro-QYKAPSW, and QYKAPSW (Fig. 2b). Interestingly, the other detected peptides seem to be derived from the non-repetitive sequence between the N-terminal signal peptide and a single arginine residue. BLAST searches using KEP 490115 reveal many other basidiomycetous KEPs with STE13 recognition motifs and core peptides with a C-terminal tryptophan (Fig. S10a).

Heterologous expression of KEP 497993 resulted in the detection of the peptide pyro-QIPAELPTKSINGNIEPYKV and 4 C-terminally truncated cleavage products thereof (Fig. 2b). These results are consistent with the N-terminal pyroglutamate protecting the N-terminus from further degradation, whereas the C-terminus appears to be further trimmed under these conditions. Four other peptides among the top 10 peptide hits were rather short peptides from the C-terminal end of the protein without obvious sequence repetitiveness. Upon heterologous expression of KEPs 490115, 497993, and 434504 (Fig. 2b and Fig. S3), several peptide products appear to result from cleavage after single arginine residues, possibly indicating that not only dibasic but also monobasic residues are cleaved by KEX2 *in vivo*, as previously shown in S. cerevisiae (38). Similarly, many products of KEP 503649 end with a lysine residue (Fig. S3). Taken together, these results confirm that our peptide extraction protocol combined with the search engine Mascot is suitable for detecting heterologously expressed KEP-derived peptides from culture supernatants of the ascomycetous yeast P. pastoris.

### KEP-derived peptides are detected in C. cinerea culture supernatant, vegetative mycelium, and fruiting bodies.

**FIG 3:**
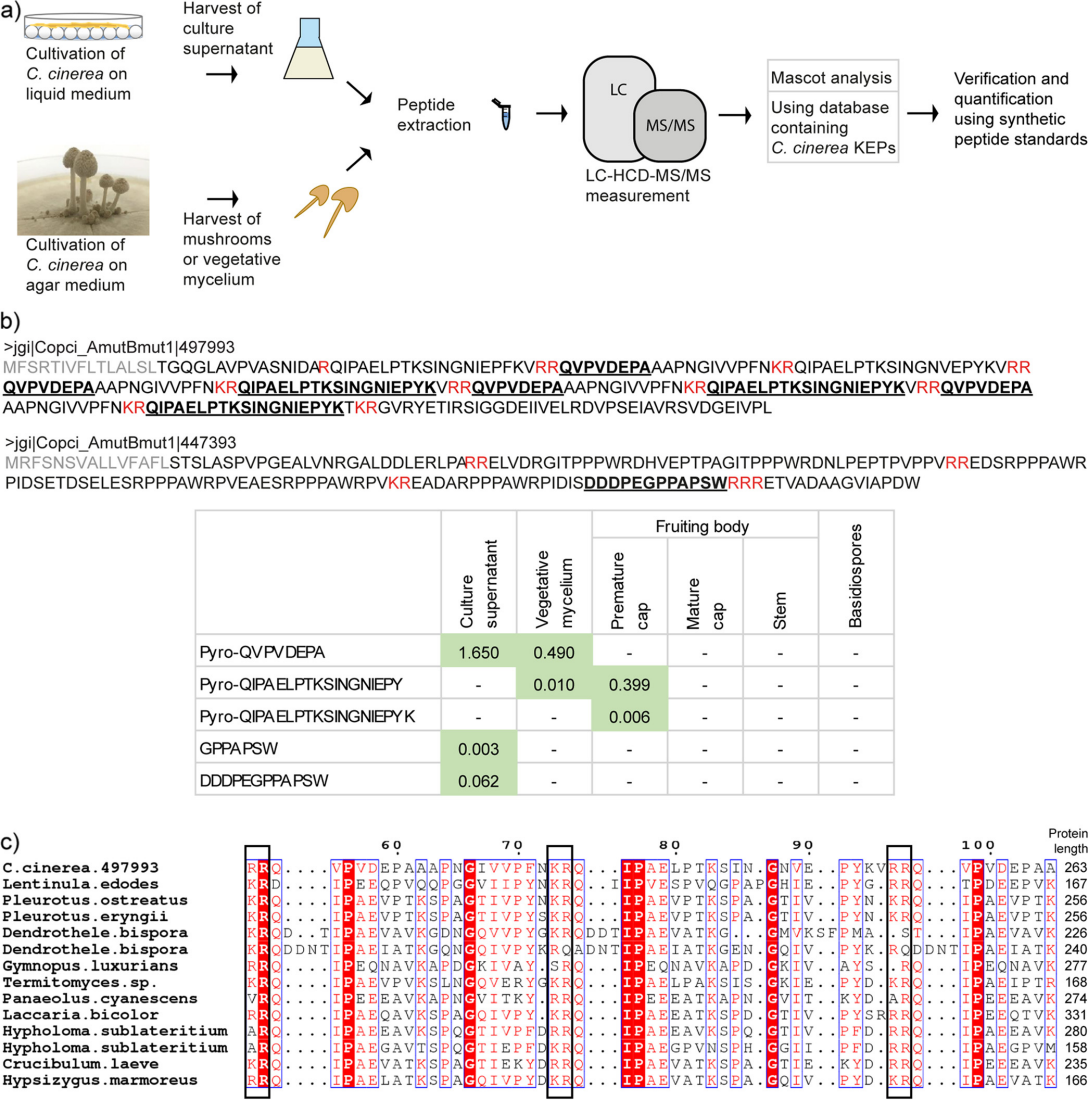
KEP-derived peptides detected in culture supernatants and tissue samples of Coprinopsis cinerea. (a) Workflow of the experimental procedure. C. cinerea was cultivated on glass beads in liquid minimal medium or on agar-solified complete medium. Peptides were extracted from the culture supernatant, vegetative mycelium, fruiting bodies, and basidiospores and analyzed using LC-HCD-MS/MS and the search engine Mascot. Potential peptide hits were confirmed and quantified using synthetic peptide standards. (b) Detected KEP-derived peptides. Three peptides, pyro-QVPVDEPA, pyro-QIPAELPTKSINGNIEPY, and pyro-QIPAELPTKSINGNIEPYK, derived from KEP 497993, were detected in the culture supernatant, vegetative mycelium, and fruiting body caps of C. cinerea. The 2 peptides GPPAPSW and DDDPEGPPAPSW, derived from KEP 447393, were detected in the culture supernatant. In the sequence of the precursor protein, the detected peptide sequences are shown in bold and underligned, the KEX2 cleavage sites in red and the signal peptide in gray. In the table below, the abundance of each peptide in the respective fungal tissues is given as peptide weight (ng) per fungal dry weight (mg). (c) Conservation of C. cinerea KEP 497993 among agaricomycetes. The alignment displays core peptides of the homologs, starting with the first KEX2 cleavage site (indicated by black boxes). The full protein length is indicated on the right.

We tested our established KEP-peptide detection protocol on culture supernatants and tissues of the agaricomycete C. cinerea. For this purpose, we cultivated C. cinerea on glass beads immersed in minimal medium, a setup that allows the fungus to form a mycelial lawn on the solid surface of the beads while secreting products into the minimal medium (36, 39). We harvested the culture supernatant and extracted the peptides. In parallel, we prepared peptide extracts from vegetative mycelium, premature and mature fruiting body caps, fruiting body stems, and basidiospores from C. cinerea cultures on agar-solidified complete medium (YMG). All samples were measured using LC-HCD-MS/MS and the resulting spectra were analyzed using the MS/MS database search tool Mascot on the basis of a database containing C. cinerea KEPs. The predicted peptides were confirmed and quantified using synthetic peptide standards (Fig. 3a).

In C. cinerea, we confirmed the presence of 3 peptides derived from KEP 497993 (pyro-QVPVDEPA, pyro-QIPAELPTKSINGNIEPY, and pyro-QIPAELPTKSINGNIEPYK) (Fig. 3b). We analyzed the presence of the KEP-derived peptides across all culture supernatant and tissue samples to obtain an overview of their expression profile. We found that the presence of each peptide was tissue-specific. The peptide pyro-QVPVDEPA was present in the supernatant of bead assay cultures and in vegetative mycelium, while being absent from fruiting body samples (Fig. 3b). In the culture supernatant, the peptide accumulated even after the fungus had fully covered the plate (4 days) and reached a maximum concentration after 14 days (Fig. S5). The peptides pyro-QIPAELPTKSINGNIEPY and pyro-QIPAELPTKSINGNIEPYK were primarily detected in the premature fruiting body cap, but were not detectable in the culture supernatant or other fruiting body samples. The precursor KEP 497993, from which all these peptides are derived, showed high transcription in the vegetative mycelium, the fruiting body, and the stem (Fig. S1). This KEP is of special interest due to its high conservation among agaricomycetes. A BLAST search revealed at least 13 homologs in other species, including the edible mushrooms Lentinula edodes, P. ostreatus, P. eryngii, and Hypsizygus marmoreus, as well as the hallucinogenic mushroom Panaeolus cyanescens (Fig. 3c and S10b), indicating a conserved function.

In addition to the peptides derived from KEP 497993, the 2 peptides GPPAPSW and DDDPEGPPAPSW from the non-repetitive C-terminus of KEP 447393 were detected in the supernatant of C. cinerea cultures (Fig. 3b). This precursor is transcribed at very low levels (Fig. S1) and the peptides were detected only in extractions of large volumes of supernatant. A BLAST search in fungal genomes for this KEP reveals 2 similar KEPs in Crassisporium funariophilum and Coprinellus micaceus (Fig. S10d).

**FIG 4:**
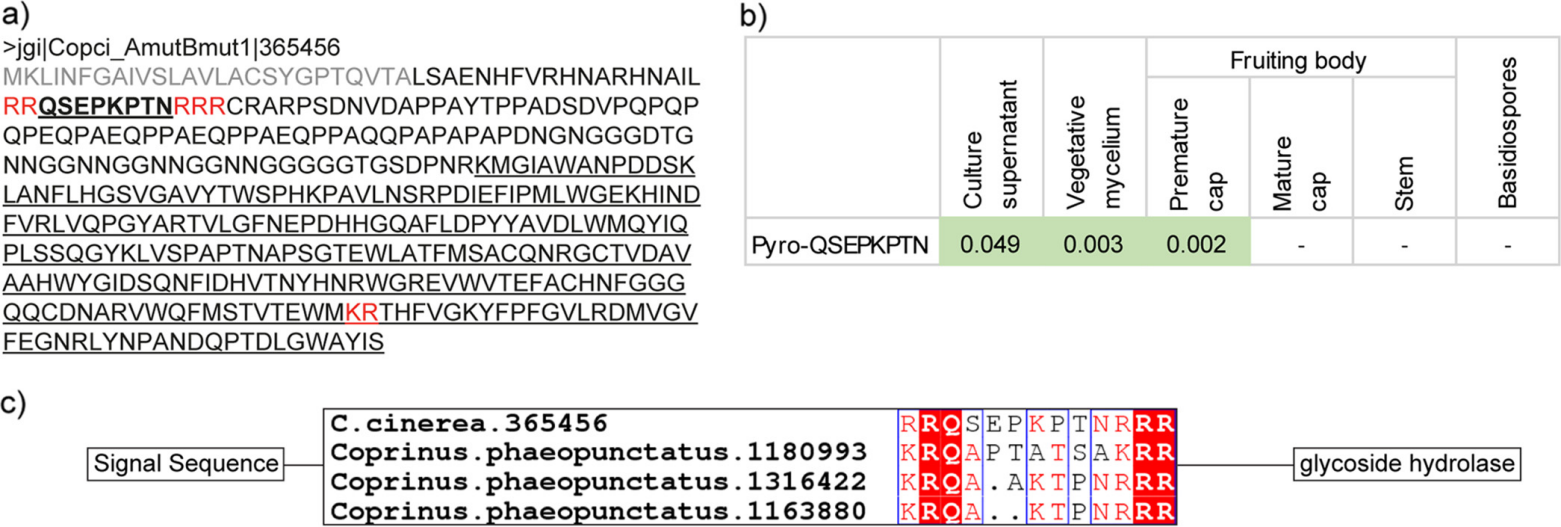
The KEX2-processed peptide pyro-QSEPKPTN is detected in culture supernatants and tissue samples of C. cinerea The peptide is produced by KEX2-cleavage of the glycoside hydrolase 128 (GH128) domain-containing protein 365456. (a) Sequence of the precursor protein. The detected core peptide is shown in bold and underlined, KEX2 cleavage sites in red, the signal peptide in gray and the glycoside hydrolase domain in underlined. (b) Detection pattern of pyro-QSEPKPTN. The peptide abundance in the respective fungal tissues is given as peptide weight (ng) per fungal dry weight (mg). The peptide was confirmed and quantified using synthetic peptide standards. (c) Schematic illustration of C. cinerea 365456 and homolog proteins in *Coprinus phaeopunctatus.* Each protein contains a signal sequence, KEX2 cleavage sites flanking a core peptide and a predicted glycoside hydrolase domain.

We performed additional Mascot data analyses by expanding our peptide search to the entire C. cinerea proteome instead of just the KEPs and then screened the results for peptides derived from potential KEX2 cleavage sites. Using this approach, we detected the peptide pyro-QSEPKPTN, derived from the KEX2 cleavage of a protein with a predicted C-terminal glycoside hydrolase 128 (GH128) domain (Fig. 4), which belongs to the same protein family as a β-1,3-glucanase in L. edodes and could have a cell-wall remodeling activity (40). The peptide was detected in supernatants of bead assay cultures, vegetative mycelium, and fruiting bodies of C. cinerea cultivated on agar-solidified medium, in agreement with the protein transcription profile (Fig. S1). Although this protein cannot be classified as a KEP due to the lack of peptide repeats, the unique protein organization with a signal sequence, a single short peptide flanked by KEX2 cleavage sites, and a C-terminal glycoside hydrolase makes this protein and its derived peptide an interesting candidate for further investigation. A BLAST search with the protein reveals 3 similar proteins in Coprinus phaeopunctatus that also contain a signal peptide for secretion, a glycosyl hydrolase domain, and KEX2 cleavage sites flanking peptides with the sequences QAPTATSA, QAAKTPN, and QAKTPN (Fig. 4c). Five other potential homologs are found in Cyathus striatus, Crucibulum laeve, Laccaria amethystina, Lyophyllum atratum, and Asterophora parasitica with the sequences ASTS, ASTS, GTTA, AANP, and ASTG, respectively, flanked by KEX2 cleavage sites (Fig. S10c).

Many of the confirmed peptides in C. cinerea are pyroglutamated. While we detected both the unmodified and pyroglutamated peptide species in the culture supernatant of KEP expressions in P. pastoris (Fig. S3e and f), this does not seem to be the case in C. cinerea where no unmodified peptide species could be confirmed (Fig. S4g). Since the genome of C. cinerea, unlike the one of P. pastoris, encodes a putative glutaminyl cyclase (Fig. S13), it seems likely that the formation of these pyroglutamated peptides in C. cinerea is an enzyme-mediated process.

### KEP-derived peptides are detected in the fruiting bodies of shiitake, oyster mushroom, and king oyster mushroom.

**FIG 5:**
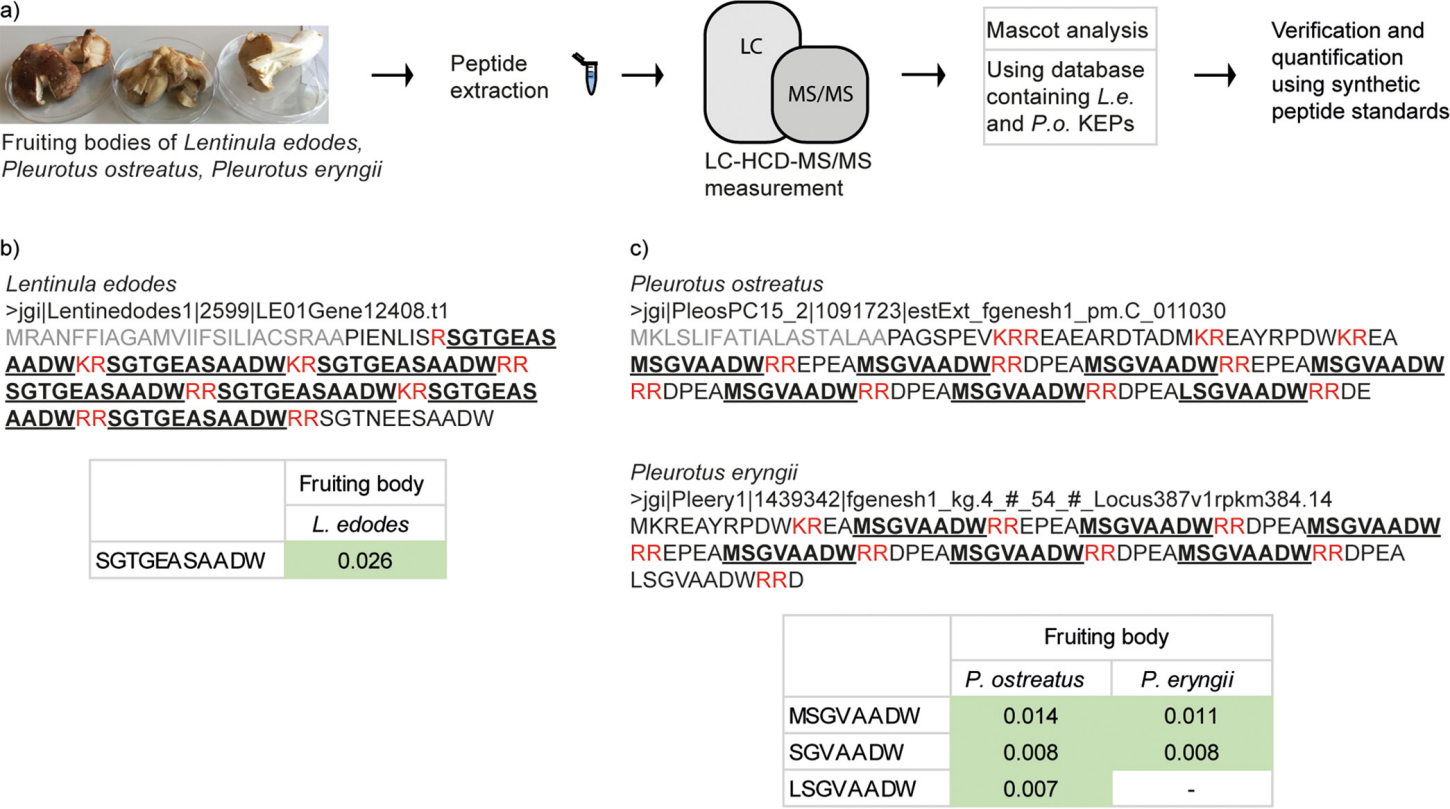
KEP-derived peptides detected in fruiting bodies of Lentinula edodes (shiitake), Pleurotus ostreatus (oyster mushroom), and Pleurotus eryngii (king oyster mushroom). (a) Workflow of the experimental procedure. Peptides were extracted from purchased fruiting bodies followed by LC-HCD-MS/MS measurements and analysis using Mascot. Peptides were confirmed and quantified using synthetic peptide standards. (b) Detected KEP-derived peptide from L. edodes and from (c) P. ostreatus and P. eryngii. The respective Pleurotus precursor proteins contain putative STE13 recognition motifs, repetitive units of XP/XA dipeptides following dibasic residues. The absence of a signal peptide in KEP 1439342 of P. eryngii is likely due to misannotation of the protein at the N-terminus (Fig. S8). The peptide abundance in the respective strains is given as peptide weight (ng) per fungal dry weight (mg).

Having demonstrated the presence of KEP-derived peptides in C. cinerea, we applied our KEP-peptide detection protocol to other agaricomycetes. Fruiting bodies of L. edodes, P. ostreatus, and P. eryngii were purchased from a local mushroom farm and peptides were extracted and analyzed by LC-HCD-MS/MS. For the identification of the KEP-derived peptides, the Mascot search engine in conjunction with the list of KEPs from L. edodes and P. ostreatus according to Le Marquer et al. 2019 and Umemura 2020 was used (Fig. 5a).

The KEP 2599 from L. edodes encodes a 7-fold repeat of the peptide SGTGEASAADW, the presence of which was confirmed in fruiting body extracts (Fig. 5b). This KEP has 4 homologs encoding nearly identical peptides in Auriculariales sp., Gymnopus luxurians, Gymnopus confluens, and Rhodocollybia butyracea (Fig. S10e). In P. ostreatus, we confirmed the presence of 3 peptide products, MSGVAADW, SGVAADW, and LSGVAADW, derived from KEP 1091723. In P. eryngii, we detected the peptides MSGVAADW and SGVAADW from the homolog KEP 1439342 (Fig. 5c). Remarkably, the latter KEPs contain not only the classical KEX2 cleavage sites flanking repetitive peptide sequences, but STE13 recognition motifs, repetitive units of dipeptides XA or XP, which are removed by dipeptidyl aminopeptidases STE13 in ascomycetes (20). A BLAST search demonstrates that STE13 homologs are present not only in C. cinerea, L. edodes, P. ostreatus, and P. eryngii, but in many more basidiomycetes (Fig. S12). Interestingly, all detected KEP-derived peptides from L. edodes and P. ostreatus/eryngii contain an identical “-AADW” sequence at their C termini. A BLAST search of these KEPs reveals some interesting homologs with STE13 recognition motifs and peptide cores with a similar C-terminus (Fig. S10f).

### Knockout of *kex2* and *kex1* genes in C. cinerea affects mycelial growth and fruiting body formation.

We established a protocol to increase the efficiency of gene knockouts in C. cinerea using cotransformation of preassembled gRNA/Cas9 ribonucleoprotein complexes, a strategy previously established in the fruiting body forming basidiomycete Schizophyllum commune (41). While establishing the protocol, we compared the knockout efficiency between the original knockout procedure, in which protoplasts derived from the vegetative mycelium of a C. cinerea AmutBmut *Δku70* strain are transformed with a plasmid carrying a gene-specific knockout cassette (42–44), and the newly established protocol in which protoplasts are transformed with both the plasmid and preassembled gRNA/Cas9 complexes. The use of CRISPR increased knockout efficiency in 3 different genes severalfold (Fig. S7a).

**FIG 6:**
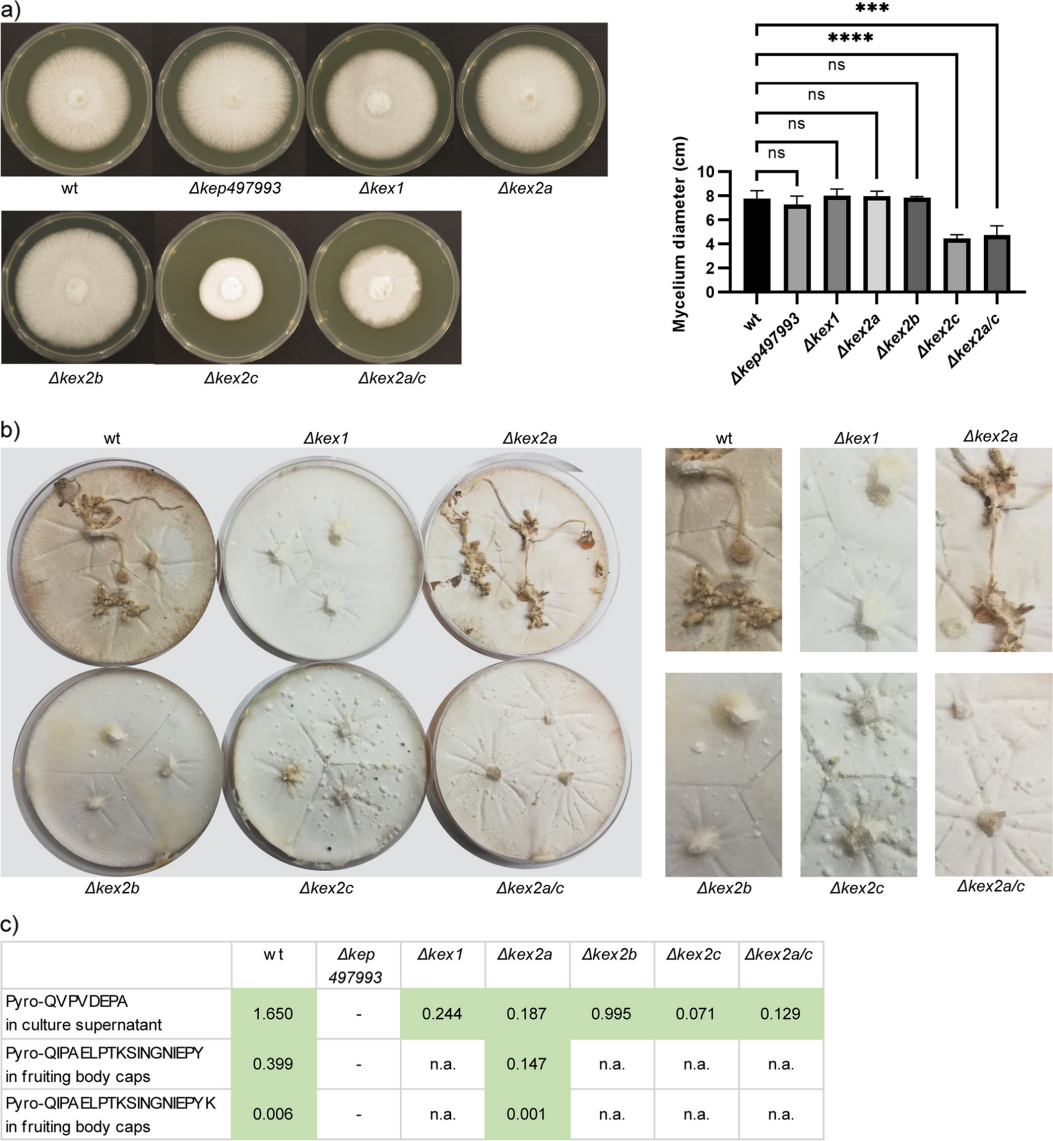
Phenotypic analysis of C. cinerea *kep* and *kex* knockout strains. (a) Mycelial growth of knockout strains on solid agar medium after 4 days with statistical analysis. *Denotes a significant difference of the mean of a group compared to the mean of a control condition (Dunnett's multiple-comparison test); bars represent the median value of 3 biological replicates, error bars represent the 95% confidence interval; ns, not significant; ****, *P* < 0.0001 (ANOVA Table S11). (b) Fruiting body formation by *kex* knockout strains. The mycelia were exposed to a night-day cycle for 40 days. During this period, the wt and *Δkex2a* strains formed multiple fruiting bodies, whereas the other strains did not. The cropped pictures on the right show the mycelium in double magnification. (c) Detection pattern of KEP 497993-derived peptides. The peptide abundance in the respective strains is given as peptide weight (in ng) per fungal dry weight (mg). The presence of the peptide pyro-QVPVDEPA was analyzed in the bead assay supernatant of wildtype (wt) and knockout strains. The same analysis was performed for the peptides, pyro-QIPAELPTKSINGNIEPY and pyro-QIPAELPTKSINGNIEPYK, in premature fruiting body caps of wildtype, *Δkex2a*, and *Δkep 497993* strains. The other *kex* knockout strains were deficient in fruiting body formation and, thus, this analysis was not applicable (n.a.).

Using the CRISPR-assisted knockout protocol, we generated C. cinerea knockout strains for the 6 KEPs 405832, 434504, 490115, 497993, 503649, and 426342. We confirmed that the peptides pyro-QVPVDEPA, pyro-QIPAELPTKSINGNIEPY, and pyro-QIPAELPTKSINGNIEPYK, which were detected in samples from the C. cinerea wildtype strain, were no longer detectable in the *Δkep 497993* mutant strain (Fig. 6c and S9b), confirming that the detected peptides were derived from this protein. All *kep* knockout strains showed normal mycelial growth when compared with the original wildtype strain macroscopically (Fig. S7n). No differences in quantity or morphology of formed fruiting bodies could be detected. In order to test a possible function of the peptides in the defense of C. cinerea against bacterial competitors, we performed bacterial growth inhibition tests using synthetic peptides and Bacillus subtilis 168, B. subtilis NCBI 3610, Micrococcus luteus, Staphylococcus aureus, and Escherichia coli BL21. No antibacterial activity was observed (Fig. S14).

Since the *kep* knockouts showed no apparent phenotype, we generated knockout strains for the 3 KEX2 homologs of C. cinerea: KEX2a (502579), KEX2b (406374), and KEX2c (448165). A sequence alignment between the KEX2 homologs of C. cinerea, P. pastoris, and S. cerevisiae demonstrates that of the 3 C. cinerea KEX2 paralogs, KEX2c has the highest sequence similarity to S. cerevisiae and P. pastoris KEX2 (Fig. S11b). There are at least 2 potential homologs for KEX1 in the C. cinerea genome, and we knocked out the one with higher sequence similarity to S. cerevisiae KEX1 (437675) (Fig. S11c). We also generated a double-knockout strain for KEX2a and KEX2c. These *kex* knockout strains were analyzed for the presence of previously confirmed KEP-derived peptides and for growth and developmental phenotypes.

The KEP-derived peptides pyro-QVPVDEPA, pyro-QIPAELPTKSINGNIEPY, and pyro-QIPAELPTKSINGNIEPYK, as well as the KEX-processed pyro-QSEPKPTN, were still present in all the *kex* knockout strains including the *Δkex2a/c* double-knockout strain (Fig. 6c and S9). The strain *Δkex2c* and the double-knockout strain *Δkex2a/c* grew more slowly on agar-solidified complete medium compared to the wildtype strain, while the other *kex* knockout strains grew normally (Fig. 6a). These results suggest that the function of the 3 C. cinerea KEX2 proteases is redundant for some substrates, e.g., the KEPs, but not for all substrates. Consistent with this conclusion, fruiting body formation was abolished in the *Δkex1*, *Δkex2b*, *Δkex2c*, and *Δkex2a/c* strains, whereas the *Δkex2a* strain and all *kep* knockout strains fruited normally (Fig. 6b). The fruiting-deficient mutant strains formed many hyphal knots with a diameter of 1–3 mm, but these did not develop further even after weeks of incubation. Taken together, these results suggest a role for KEX2c in mycelial growth and for KEX1, KEX2b, and KEX2c in fruiting body formation. KEX2a and the 6 investigated KEPs do not appear to be involved in these processes.

## DISCUSSION

In this work, we have established a protocol for the detection of KEP-derived peptides in a variety of fungal tissues. These secreted, ribosomally synthesized fungal peptides represent a very interesting class of natural products, although they are only minimally modified compared to more complex fungal RiPPs. KEPs have been shown to be widely distributed in fungi (15, 16), and the sequence diversity of predicted KEP-derived peptides is huge. Therefore, the analysis of KEPs has a high potential for the discovery of novel peptides with interesting bioactivities. The already characterized examples function as mating factors (21), virulence factors (17), and mutualism modulators (45) and are, thus, mostly involved in biotic interactions of the respective fungi.

Using our detection protocol, we confirmed the presence of several KEP-derived peptides in C. cinerea, L. edodes, and P. ostreatus*/*eryngii (Fig. 3 and 5). Interestingly, we observed that certain peptides, although derived from the same precursor protein, were exclusively detected in specific tissues, indicating that there is a tissue-specific processing of the precursor protein or of the derived peptides. This tissue-specificity seems unrelated to KEX protease expression levels, as all these processing enzymes are expressed at approximately the same levels in the respective tissues (Fig. S1). Some of the detected KEP-derived peptides likely originate from STE13-mediated processing, just as the α-pheromones of ascomycetes (Fig. 5). To our knowledge, this is the first indication of STE13 dipeptidyl aminopeptidase activity in a basidiomycete, and the first demonstration that these α-pheromone-like peptide precursor proteins are not only present in the predicted proteome of basidiomycetes but are also actively processed to peptides. Further studies are needed to elucidate the function of these peptides in basidiomycetes.

The established knockout strains for 6 different KEPs in C. cinerea did not show any apparent phenotype, indicating that these peptides are not critical for axenic growth and development under the used growth conditions but may rather be involved in the communication with the biotic environment. KEP-derived peptides were present in all *kex* knockouts (Fig. 6), suggesting that there is redundancy among the 3 proteases regarding KEP-processing. The apparent redundancy regarding KEPs does not seem to apply to all targets of KEX proteases, as the individual *kex2b*, *kex2c*, and *kex1* knockout strains displayed aberrant growth and development phenotypes.

We observed defects in mycelial growth in C. cinerea *kex2c* knockout strains. Mycelial growth defects or abnormal hyphal morphology are typical phenotypes of *kex2* knockouts in ascomycetes. Examples are hyperbranching, thickened cell walls, and upregulation of cell wall stress response genes in Aspergillus niger (46, 47), hyperbranching and disordered cell integrity signaling in A. oryzae (48), hypersensitivity to fungicides targeting the cell membrane in C. glabrata (49), and lack of hyphae formation in C. albicans (50). The ascomycetous strains in which *kex2* knockouts were established possess only one *kex2* gene in contrast to the three *kex2* genes of C. cinerea and other agaricomycetes (51), implying a diversification of KEX2 function in agaricomycetes. Consistent with the high similarity of C. cinerea KEX2c with S. cerevisiae and P. pastoris KEX2 (Fig. S11b), only knockouts of the *kex2c* gene reduced mycelial growth in C. cinerea. Inhibition of fruiting body formation was observed in knockout strains of *kex1*, *kex2b*, and *kex2c* (Fig. 6c). Since no *kex* knockout strains have been established in mushroom-forming fungi before, this is a previously undescribed phenotype. Both *Δkex2b* and *Δkex2c* exhibited fruiting deficiencies, implying that they cannot substitute for each other's function and are both required for efficient fruiting body formation via processing of one or more targets. The fruiting deficiency of *Δkex1* suggests that the same target(s) may be involved and that they require processing by KEX2b, KEX2c, and KEX1. In contrast, defects in mycelial growth were observed only in the *Δkex2c* strain, suggesting that a different target is affected that does not require processing by KEX1.

KEX2 and KEX1 proteases process not only KEPs but also several cell wall modulating enzymes and structural proteins that pass the secretory pathway, e.g., by removing N-terminal or C-terminal propeptides (52–54). In previous experiments in ascomycetes, knockout of *kex2* resulted in a phenotype similar to knockouts of KEX2-processed cell wall remodeling enzymes or components, e.g., increased sensitivity to fungicides targeting the cell wall (55, 56). Therefore, we hypothesize that the observed phenotypes of C. cinerea *kex* knockout strains are due to an impairment in protein processing rather than to an impairment in KEP-processing. This hypothesis is also supported by the observation that KEP-derived peptides are still present in *kex* knockouts.

Further work is needed to decipher the function of the KEP-derived peptides and the molecular basis of the phenotypes of *kex* knockout mutations in C. cinerea. The specificity of basidiomycetous KEX proteases should be further investigated, and a bioinformatic screen could be performed to find potential KEX2 substrates with putative carbohydrate-active enzymatic domains. According to reports in ascomycetes, such proteins could be responsible for the observed *Δkex2* phenotypes. Protein 365456 that contains KEX2 cleavage sites and a C-terminal glycoside hydrolase domain may fall in this class.

### Conclusion.

Our results suggest that fungi, including the agaricomycetes C. cinerea, P. ostreatus, P.eryngii, and L. edodes, secrete a variety of linear and minimally modified ribosomal peptides. These peptides derive from secreted precursor proteins (KEPs) that are processed in the Golgi apparatus by the endoproteinase KEX2 and the carboxypeptidase KEX1. While knockouts of C. cinerea *kex* genes resulted in defects in mycelial growth and differentiation, knockouts of *kep* genes showed no phenotype under axenic conditions. These results suggest that the KEP-processing enzymes target a wide variety of substrates, some of which play a role in mycelial growth and differentiation, but that the investigated KEP-derived peptides are likely to rather play a role in the interaction of the mushroom with the biotic environment.

## MATERIALS AND METHODS

### Strains.

A full list of all used strains can be found in the Table S3. C. cinerea samples were extracted from strains AmutBmut or AmutBmut *Δku70*. The same *Δku70* strain was used to establish all knockout cell lines and is therefore labeled as wildtype (wt) throughout the paper. Peptides derived from the KEP 497993 were confirmed in the strain AmutBmut *Δku70* for comparison to the *Δku70*-based knockout strains, all other peptides were confirmed in AmutBmut. Fresh fruiting bodies of L. edodes (strain 4312, Sylvan), P. ostreatus (strain P24/HK35, Sylvan), and P. eryngii (strain 3066, Sylvan) were purchased from a grocery store and processed immediately. P. pastoris strain GS115 was ordered from Invitrogen. Molecular cloning was performed using E. coli DH5α, protein expression of Cas9 was performed using E. coli BL21.

### Genomic screening for KEX2-processed repeat proteins in C. cinerea.

The predicted proteome of C. cinerea AmutBmut pab1.2 was downloaded from JGI in early 2018 («*Copci_AmutBmut1_GeneModels_FrozenGeneCatalog_20160912_aa.fasta*») (25, 26) (https://mycocosm.jgi.doe.gov). Two different methods were tested to screen for KEPs. For the first method, a Java script was written that cleaved the proteome *in silico* at the dibasic residues KR, RR, KK, and RK. These 4 dibasic cleavage sites were chosen based on the processing of KEP-homolog neuropeptide precursors. After *in silico* cleavage, the cleavage products of individual proteins were compared using the protein BLAST+ command line application (version 2.7.1) (27, 28). In a first screen, sequences were considered to be similar if they reached a percentage identity of 70%. Similarity was assessed only for cleaved fragments with a length difference of fewer than 30 amino acid residues. To account for short repetitive sequences for which a percentage identity threshold of 70% was too strict, a second screen was performed in which short cleaved fragments (<20 residues) with a length difference of fewer than 8 residues were assessed for their percentage identity and considered similar if they scored at least 40%. Results from both screens were combined and filtered: Excluded were proteins with no signal sequence (assessed using SignalP 4.0) (57), fewer than 2 cleavage sites and protein lengths greater than 800 amino acids, as 96% of known neuropeptide precursors were below that threshold (58). KEPs had to contain at least one repetitive partial sequence that was similar to at least 2 other partial sequences. The java script is deposited on zenodo (https://doi.org/10.5281/zenodo.7034114). In the second screening method, only proteins with a signal sequence and shorter than 300 amino acids were analyzed. Repetitive sequences in these proteins were highlighted using the rapid automated detection and alignment of repeats tool (RADAR) (29). The output was visually inspected and proteins with a KEP-like architecture were manually picked.

### Heterologous expression of KEX2-processed repeat proteins in P. pastoris.

All gene and primer sequences are listed in the Table S4 and S5. RNA was isolated from C. cinerea mycelium using the Norgen RNA extraction kit (Norgen Biotek Corporation) according to the manufacturer’s protocol. cDNA was prepared using the Transcriptor first strand cDNA synthesis kit (Roche) using oligo-dT primers. The cDNA was used for amplifying intron-less KEP gene sequences via PCRs, which were then ligated into pGEM T-easy vectors (Promega). This was done for the KEPs 405832, 434504, 497993, 503649, and 426342. The coding region of KEP 490115 could not be amplified from cDNA, possibly due to highly repetitive nucleotide regions in the peptide cores and was instead ordered from Twist Bioscience in a codon optimized form. All plasmids were transformed into E. coli DH5α and isolated using miniprep kits (Qiagen). The coding regions were subsequently amplified from the plasmids using primers with a 5′ restriction site overhang. These amplified products were digested and ligated into pPICZA vectors (ThermoFisher Scientific). The stop codons of the *kep* genes were removed in the PCR primers to allow for C-terminal fusion of the proteins to the myc and 6×His tags on the PICZA vector. The PICZA vectors were amplified in E. coli DH5α, purified using miniprep, the sequence confirmed via Sanger sequencing, the plasmids linearized with the restriction enzyme PmeI and then used for transformation of Pichia pastoris GS115 by electroporation with 1.2 kV of charging voltage using a Bio-Rad MicroPulser electroporator. Positive clones were selected for on YPD plates (1% (wt/vol) yeast extract, 2% (wt/vol), peptone, 2% (wt/vol) glucose, 2% (wt/vol) agar containing 0.2 mg/mL zeocin. Successful integration of pPICZA at the AOX1-locus was tested via colony PCR. For heterologous expression of C. cinerea KEP genes, P. pastoris transformants were cultivated until they reached OD 2 in buffered minimal glycerol medium (BMGH) (100 mM potassium phosphate [pH 6.0], 1.34% yeast nitrogen base [YNB, with ammonium sulfate and without amino acids] [wt/vol], 4 × 10^−5^% biotin [wt/vol], 0.004% histidine [wt/vol], 1% glycerol [vol/vol]). The culture was spun down at 2000 rcf for 10 min, the pellet resuspended in buffered minimal methanol medium (BMMH) (100 mM potassium phosphate [pH 6.0], 1.34%, YNB [wt/vol], 4 × 10^−5^% [wt/vol], biotin [wt/vol], 0.004% histidine [wt/vol], 0.5% methanol [vol/vol]), spun down again, and then resuspended to an OD of 1. The culture was then incubated for 3 days at 30°C under constant shaking, with an addition of 0.5% (vol/vol) methanol to the medium every 24 h. Afterwards, the culture was spun down with 3000 rcf for 10 min. The cell pellets were washed with 2x PBS, lysed using glass beads using a Thermo FastPrep FP120 cell disruptor and used for an immunoblot to confirm successful expression of the tagged KEP. His-tagged proteins on the blots were detected using anti-His antibodies (Qiagen). The supernatant was harvested, filtered through a 10 kDa Amicon filter, and 10 mL were subsequently used for extraction of peptides.

### Cultivation of C. cinerea and harvest of fungal samples.

Cryostocks (oidia) of C. cinerea were revived on YMG 1.5% agar plates (0.4% [wt/vol] yeast extract, 1% [wt/vol] malt extract, 0.4% [wt/vol] glucose) and allowed to grow for 4 days at 37°C in a dark aerated box with wet tissue paper. A mycelial plug was cut from the edge of the mycelium and transferred to a YMG plate to be incubated again for 4 days. Bead assays with liquid minimal medium (per L of medium: 5 g glucose, 2 g asparagine, 50 mg adenine sulfate, 1 g KH_2_PO_4_, 2.3 g Na_2_HPO_4_ [anhydrous], 0.3 g Na_2_SO_4_, 0.5 g C_4_H_12_N_2_O_6_, 40 g thiamine-HCl, 0.25 g MgSO_4_, and 5 mg p-aminobenzoic acid) were then set up as previously reported (36, 39): Three agar plugs were cut from the edge of a C. cinerea mycelial colony and transferred to a petri dish filled with approximately one layer of sterile borosilicate glass beads (5 mm) and 15 mL of minimal medium. The fungus was allowed to grow for 4 days under the usual growth conditions (37°C, darkness) until harvest of the liquid medium. For the comparison between different knockout strains, 125 μL of media were harvested. For the time course samples, 125 μL of media were taken continuously over 28 days from the same culture. For the rest of the samples, 1 mL of supernatant were harvested. The liquid medium was filtered through a 10 kDa filter (Amicon), snap-frozen and then used for peptide extraction. For the harvest of dry vegetative mycelium, the fungus was cultivated by transferring three agar plugs to an YMG agar plate covered with one sheet of sterile cellophane. The fungus was then allowed to grow on top of the cellophane for 4 days at 37°C in darkness, before the mycelium was scratched from the cellophane and snap-frozen. For the harvest of fruiting bodies, thick YMG agar plates (containing 35 mL of medium) were inoculated with 3 agar plugs of C. cinerea and incubated for 4 days at 37°C in darkness (6 days for the growth deficient Δkex2c strains). The plates were then moved to a fruiting body room with 25°C, 85% humidity and a day-night cycle of 12 h of light and 12 h of darkness. After approximately 10 days (AmutBmut) or 20 days (AmutBmut *Δku70*) under these conditions, fruiting bodies started to form on the plates. The fruiting bodies were harvested when their stipes were fully expanded, and their cap was still half-closed in the middle of the “night” phase (premature caps and stipes) or 4 h later when the caps had fully opened and started to darken (mature caps). In addition, basidiospores were isolated by harvesting multiple mature dark caps and their “ink,” the black liquid within the area. The samples were resuspended in water, filtered through glass-wool funnels and the basidiospores were pelleted by centrifuging. All tissue samples were flash-frozen in liquid nitrogen and dried in a Speedvac (SPD111V, ThermoFisher Scientific) before peptide extraction.

### Peptide extraction.

For peptide extractions from liquid medium, including C. cinerea bead assay samples and P. pastoris supernatant samples, samples were either purified using Oasis HLB 1 cc vac cartridges (10 mg sorbent, 30 μm, Waters, US), Sep-Pak 1 cc cartridges (10 mg sorbent, 30 μm, Waters, US) or first dried using speedvac and then purified using SP3 (Single-pot solid-phase-enhanced sample preparation) (59). For initial peptide extractions of P. pastoris samples, extraction of KEP 497993 heterologous expression samples was tested using Sep-Pak C18 and Oasis HLB cartridges. The concentration of extracted peptides was highest with HLB cartridges, so they were used for extraction of all Pichia samples. For peptide extractions from fungal tissues, 30 mg of fruiting body caps, stipes and mycelium or 10 mg of basidiospores were soaked in 1.5 mL of methanol for 1 h at 37°C while gently shaking. The methanol was then moved to a new tube and evaporated by speedvac. Other tested parameters included different solvents like water and acetonitrile and different soaking temperatures of room temperature, 37°C and 99°C (for water). After drying, the samples were reconstituted in ddH2O and passed through a 10 kDa Amicon filter. The peptides were then either purified using HLB columns, or dried using a speedvac for SP3. Solid phase extraction using HLB and Sep-Pak cartridges were done according to the manufacturer’s protocol. In brief, the cartridges were conditioned with 1 mL of methanol, equilibrated with 1 mL of ddH2O and loaded with the aqueous samples. The cartridges were washed with 1 mL of 5% methanol and then eluted with 1 mL of 100% methanol. The samples were dried in a speedvac and resuspended in MS buffer (3% acetonitrile, 0.1% formic acid). For SP3 peptide extraction, dried samples were resuspended in 25 μL of 50 mM HEPES buffer and processed following the official guidelines with modifications (59). Magnetic hydrophilic and hydrophobic beads (Sera-Mag SpeedBead Carboxylate-Modified Magnetic Particles, GE Life) were mixed 1:1 and washed three times with ddH2O at a concentration of 10 μg/μL and then resuspended at a concentration of 5 μg/μL. The samples were then gently mixed with 15 μL of bead suspension. A total of 800 μL acetone were added and the samples carefully mixed. Samples were incubated at room temperature for 8 min, then moved to a magnetic rack for 2 min. The supernatants were removed, and the beads washed twice with 200 μL of 95% acetonitrile. Then, the beads were resuspended in 25 μL of 2% DMSO for peptide elution. The samples were sonicated for 1 min and incubated at room temperature for 5 min. The tubes were moved to the magnetic rack and the supernatant transferred to a new tube. The samples were dried by speedvac and redissolved in MS buffer. The approximate peptide concentration was determined using nanodrop (Witec) and samples were diluted to concentrations of 2 mg/mL.

### LC-HCD-MS/MS measurements and analysis by Mascot.

Samples were measured using liquid chromatography higher-energy collisional dissociation tandem mass spectrometry (LC-HCD-MS/MS) on a Q Exactive HF (Thermo Scientific) coupled to an ACQUITY UPLC M-Class system. Chromatographic separation was performed using trapped elution using a nanoEASE Symmetry C18 100 A column (5 μm, 1/PK 180 μm × 20 mm), followed by analytical elution using a nanoEase HSS C18 T3 100 A column (1.8 μm, 1 P/K 75 μm × 250 mm). Solvent A was water with 0.1% formic acid (vol/vol) and solvent B was acetonitrile with 0.1% formic acid. For the initial measurement of P. pastoris supernatant samples as well as C. cinerea supernatant and tissue samples, the samples were measured using an untargeted approach. Peptides were eluted with a linear gradient from 5% to 30% solvent B in 90 min at a flow rate of 0.3 μL/min, followed by a linear gradient from 30% to 95% in 5 min and a return to 5% in 10 min. Full MS was measured using data-dependent acquisition with a resolution of 120 000, automated gain control (AGC) target 3 × 10^6^, scan range 350 to 1500 *m/z* and maximum IT 50 ms. MS/MS spectra acquisition was performed using a resolution of 30 000, AGC target 10^5^, maximum IT 50 ms. The minimum AGC was 4.5 × 10^3^ and the dynamic exclusion time 30 s.

In the subsequent Mascot search, the mgf file containing all MS/MS spectra of the measurement was searched against databases containing the C. cinerea KEPs, the C. cinerea proteome (C. cinerea AmutBmut *pab1.2*, from https://mycocosm.jgi.doe.gov, manually annotated) or L. edodes and P. ostreatus KEPs (15, 16). The search settings were: no enzyme cleavage, error windows of 10 ppm for peptide tolerance and 0.05 Da for MS/MS tolerance, measured on a Q exactive instrument and with an enabled decoy database. Variable modifications that were tested included N-terminal pyro-glutamation, C-terminal amidation, hydroxylation, oxidation, and methylation. To avoid false positives, a false-discovery-rate was calculated, based on matches from a “decoy” database containing only randomized and inverted sequences. No true matches are expected from the decoy database, so the number of false matches is a good estimate of the number of false positives from the target database. In the analysis of P. pastoris samples, we set the false-discovery rate in Mascot to 1%, meaning that the risk of a false positive being called significant is lower than 1%. For all the potential KEP-derived peptide candidates from C. cinerea, L. edodes, P. ostreatus, and P. eryngii, the Mascot hits were manually screened for interesting peptide candidates. Peptides with fewer than 6 residues were excluded, while modified peptides or peptides with a promising sequence (e.g., a repetitive core region flanked by KEX2 cleavage sites) were chosen for a verification using synthetic peptides. We ordered synthetic peptides at Genscript Biotech (pyro-QVPVDEPA) and EZBiolab (all other peptides) at 85% purity for verification. We measured the synthetic peptides with a concentration of 100 ng/mL. An initial comparison of the spectra of the synthetic peptides to the fungal samples was done using the software Skyline 20.2.0.343 (MacCoss Lab Software) on the basis of the (isotope) dot product ([i]dotp). For a final confirmation of peptide presence and for figures, the retention times and MS/MS spectra were compared manually in the software Xcalibur (Thermo Scientific). Once the peptides were confirmed, samples were remeasured using a targeted approach with an inclusion list containing the masses of the peptides of interest. For the time course samples (Fig. S5) and the knockout comparison samples (Fig. 6), a shorter linear gradient was used from 5% to 30% acetonitrile in 50 min, followed by a linear gradient from 30% to 95% in 5 min and a return to 5% in 10 min. We performed BLAST searches in the respective fungal species using the confirmed peptide sequences to ensure that they do not arise from cleavage of another protein. The abundance of peptides in fungal samples was quantified by comparing the peak area values of the extracted ion chromatogram to the respective synthetic peptides.

### Generation of C. cinerea knockout strains.

For the establishment of knockout strains in C. cinerea, we used the para-aminobenzoic acid synthase-encoding gene (*pab1*) that allows growth of *pab1*-mutant strains AmutBmut and AmutBmut *Δku70* on para-aminobenzoic acid-deficient medium as selection marker. In order to avoid recombination of the marker with the C. cinerea *pab1* locus, we used a previously constructed, heterologous *pab1* cassette (Pcpab1) consisting of the Phanerochaete chrysosporium *pab1* gene under the control of the Agaricus bisporus *gpdII* promoter and P. chrysosporium *mnp* terminator regions (44). For each knockout, we cloned a template plasmid with the Pcpab1 cassette flanked by 750–1200 nt homology arms of the respective gene, as described by Stöckli et al., 2017. The homology arms were first amplified from C. cinerea gDNA using primer pairs containing a short *Pcpab1* overhang, while the *Pcpab1* cassette was amplified from the template plasmid using primer pairs containing a short overhang of the homology arms. All 3 PCR products, meaning the 5′ homology arm, the Pcpab1 cassette, and the 3′ homology arm, were then assembled in a one-step overlap extension PCR and ligated into a pGEM T-easy vector (Promega). crRNAs were designed to cut proximal to the 5′ and 3′ regions of the genes. The design tool CRISPR RGEN Tools (http://www.rgenome.net/cas-designer) was used to design and test the crRNAs for potential off-target effects and the crRNAs were ordered at Integrated DNA Technologies (IDT) and hybridized with the tracrRNA (IDT) to form the gRNA. The S. pyogenes Cas9 enzyme (60) was heterologously produced in E. coli BL21 via the plasmid pET-NLS-Cas9-6×His from Addgene and the enzyme was isolated and purified using Ni-NTA beads. For all C. cinerea transformations we used the strain AmutBmut pab1.2 *Δku70* that contains a knockout of the ku70 gene which reduces the frequency of non-homologous end-joining (41, 61). The protoplasting of C. cinerea mycelia was done as described in Nakazawa et al. 2010 [62]). In short, C. cinerea was grown on cellophane-covered YMG agar for 3 days at 37°C in darkness, until the mycelium was scraped off, mixed with YMG medium and blended using a Waring laboratory blender (US). The blended mycelium was then grown in shaking flasks in liquid YMG medium for 2 days. The mycelium was harvested and incubated for 1 h with protoplasting enzyme mix containing cellulase (“Onozuka R-10,” Serva) and chitinase (“C-6137,” Sigma). The protoplasts were then filtered through funnels with Miracloth, mixed with the template DNA and pre-assembled gRNA/Cas9 complexes as described by Jan Vonk et al. 2019 (41) and transformed in a heat shock incubation at room temperature. The transformation mixtures were then plated on para-aminobenzoic acid-deficient agar plates and growing colonies were picked and transferred to new plates. These colonies were then screened in PCRs and a final confirmation of knockout was done via southern blotting. The KO strains of *kex2b* and *kex1* were produced using oidial protoplasts, meaning that plates covered with C. cinerea mycelia were moved to constant light for 3 days and the oidia were rinsed and filtered from the plates through glass-wool funnels. They were treated with the same enzyme mix as the mycelium for generation of protoplasts. The *kex2c* KO and the double KO strain of *kex2a* and *kex2c* (labeled *Δkex2a/c*) were both produced in a transformation reaction where the gRNAs and template plasmids of KEX2a, KEX2b and KEX2c were all added to the same reaction and the subsequent colonies were tested for KO of all 3 KEX proteases.

### Southern blots.

As a final confirmation that the constructed knockout strains no longer contained the wildtype gene, we performed southern blots on all knockout strains according to the protocol by Wälti et al., 2006 (63). In short, DIG-labeled hybridization probes (labeled dNTPs from Roche) for one homology arm of the respective genes were amplified in a PCR from the respective template plasmid that was used for transformation of the wildtype strain. These PCR samples were run on an agarose gel and the band of the correct size was purified. Five micgrogram of gDNA isolated from the respective knockout strains were digested with the chosen restriction enzyme and the samples were run on an agarose gel, from which they were transferred to a Hybond-N Nylon membrane (Cytiva). The membranes were hybridized with the DIG probes, stained with an anti-DIG-antibody (Roche), and imaged using CDP-Star substrate (Roche).

### Phenotypic comparisons of knockout strains.

For a comparison of mycelial growth speed, knockout strains were cultivated on YMG plates with a single inoculum and imaged after 4 days of incubation at 37°C in darkness. For a comparison of peptide presence between *kex and kep* knockout strains, the strains were cultivated in bead assays as described earlier. Samples were taken after 4 days. For the strains *Δkex2c* and *Δkex2a/c*, an additional set of plates was prepared whose supernatant was harvested after 6 days to account for the slower growth speed of these strains. For a comparison of fruiting body formation, thick YMG agar plates were inoculated with three agar plugs. After 4 days of growth, plates were almost covered with mycelium (with the exception of strains *Δkex2c* and *Δkex2a/c* which were left to grow for total 6 days), were sliced with a scalpel to induce the formation of more fruiting bodies and moved to a fruiting room with 25°C, 85% humidity and a day-night cycle of 12 h of light and 12 h of darkness. Six replicates of plates were left in the fruiting room for continued observation for several weeks, without any fruiting bodies forming for *Δkex1*, *Δkex2b*, *Δkex2c*, and *Δkex2a/c*.

### Data availability.

The mass spectrometry data have been deposited to the ProteomeXchange Consortium via the PRIDE (64) partner repository with the data set identifier PXD036934. A list of the individual data sets is provided in Table S11.
