# Supplementary material for: Structural and Functional Analysis of Peptides Derived from KEX2-Processed Repeat Proteins in Agaricomycetes Using Reverse Genetics and Peptidomics
Source: Microbiol Spectr. 2022 Oct 31;10(6):e02021-22. doi: 10.1128/spectrum.02021-22 (PMC9769878; doi:10.1128/spectrum.02021-22)
Supplement: Supplemental file 1 — Supplemental material. Download spectrum.02021-22-s0001.pdf, PDF file, 7.4 MB [file spectrum.02021-22-s0001.pdf]

Supplemental Material

Structural and functional analysis of peptides derived from KEX2-processed repeat proteins in agaricomycetes using reverse genetics and peptidomics

Eva Vogt[1], Lukas Sonderegger[1], Ying-Yu Chen[1], Tina Segessemann[1], Markus Künzler[1]*

[1] ETH Zürich, Department of Biology, Institute of Microbiology, Vladimir-Prelog-Weg 4, CH-8093 Zürich, Switzerland

*Corresponding author

Contents

Supplemental Materials and Methods: Antimicrobial assays using synthetic peptides. 3

Supplemental Figures 4

Figure S1: Gene transcription profiles of the *C. cinerea kep* genes, the gene 365456, *kex* genes, *ste13,* and a glutaminyl cyclase gene. 4

Figure S2: Verification of expression of *C. cinerea* KEPs in *P. pastoris*. 5

Figure S3: KEP-derived peptides can be detected in the culture supernatant of *Pichia pastoris* upon expression of *C. cinerea* KEPs. 7

Figure S4: Extracted ion chromatograms and MS/MS spectra of peptides in *C. cinerea* tissues...... 14

Figure S5: Presence of the KEP-derived peptide pyro-QVPVDEPA in time-course sampling of *C. cinerea* culture supernatant. 15

Figure S6: Extracted ion chromatograms and MS/MS spectra of peptides in *L. edodes, P. ostreatus,* and *P. eryngii* fruiting bodies. 18

Figure S7: Establishment of *kex* and *kep* knockout strains in *C. cinerea*. 19

Figure S8: Analyis of gene annotation for the gene 439342 from *P. eryngii.* 21

Figure S9: Extracted ion chromatograms and MS/MS spectra of peptides of all constructed *C. cinerea* knockout strains. 26

Figure S10: Alignments of analyzed KEX2-cleaved proteins with homologs in other fungal species. 31

Figure S11: Alignments of *C. cinerea* KEX proteases with homologs in other fungal species........... 35

Figure S12: Homologs of dipeptidyl aminopeptidases STE13 in different fungal species. 38

Figure S13: Alignments of glutaminyl cyclase (QC) homologs across different species. 40

Figure S14: Bacterial growth inhibition assays of synthetic KEP-derived peptides. 41

Supplemental Tables 43

Table S1: KEX2-processed repeat proteins (KEPs) of *C. cinerea*. 45

Table S2: KEX2-processed repeat proteins (KEPs) in *L. edodes* and *P. ostreatus* detected by Le Marquer et al., 2019 and Umemura 2020. 46

Table S3: Strains used in this study. 48

Table S4: Primers used in this study. 50

Table S5: Coding sequences of heterologously expressed *kep* genes. 52

Table S6: Plasmids used in this study. 52

Table S7: crRNAs used in this study. 53

Table S8: Reference genomes used in this study. 55

Table S9: JGI protein IDs of proteins used in this study. 56

Table S10: ANOVA results of mycelial diameter comparison between *C. cinerea* knockout strains.56

Table S11: List of proteomics data deposited to the ProteomeXchange Consortium. 57

Supplemental references 58

Supplemental Materials and Methods: Antimicrobial assays using synthetic peptides.

For the disk diffusion assay, the bacterial strains *B. subtilis* 168*, B. subtilis* NCBI 3610*, Micrococcus luteus, Staphylococcus aureus,* and *E. coli* BL21 were streaked on agar-solidified LB plates and incubated overnight at 37°C. Freshly grown colonies were resuspended in 1 ml liquid LB medium to an OD_{595} of 0.1 and 150 µl of culture suspensions were spread evenly on agar-solidified LB plates. Peptides were not tested individually. Instead, three or four peptides were combined in peptide mixtures and tested together. Filter paper disks were loaded with 70 nmol of each individual peptide or 5 µg of vancomycin hydrochloride (from *Streptomyces orientalis*, Merck, Germany), or ampicillin sodium salt (BioChemica, AppliChem GmbH, Germany) as positive controls. Bacterial growth was checked after incubation at 37°C for 24 h. For bacterial assays using an optical density (OD) readout, resuspended bacteria were allowed to grow to an OD_{595} of 0.5 in liquid LB medium at 37°C while shaking, were then diluted to an OD_{595} of 0.1, and incubated in triplicates in 96-well microtiter plates at a concentration of 0.5 mM for each peptide mixture and 50 µg/ml of vancomycin or ampicillin. The OD_{595} was measured after 24 hours and the change of optical density was calculated for each microtiter well.

Supplemental Figures

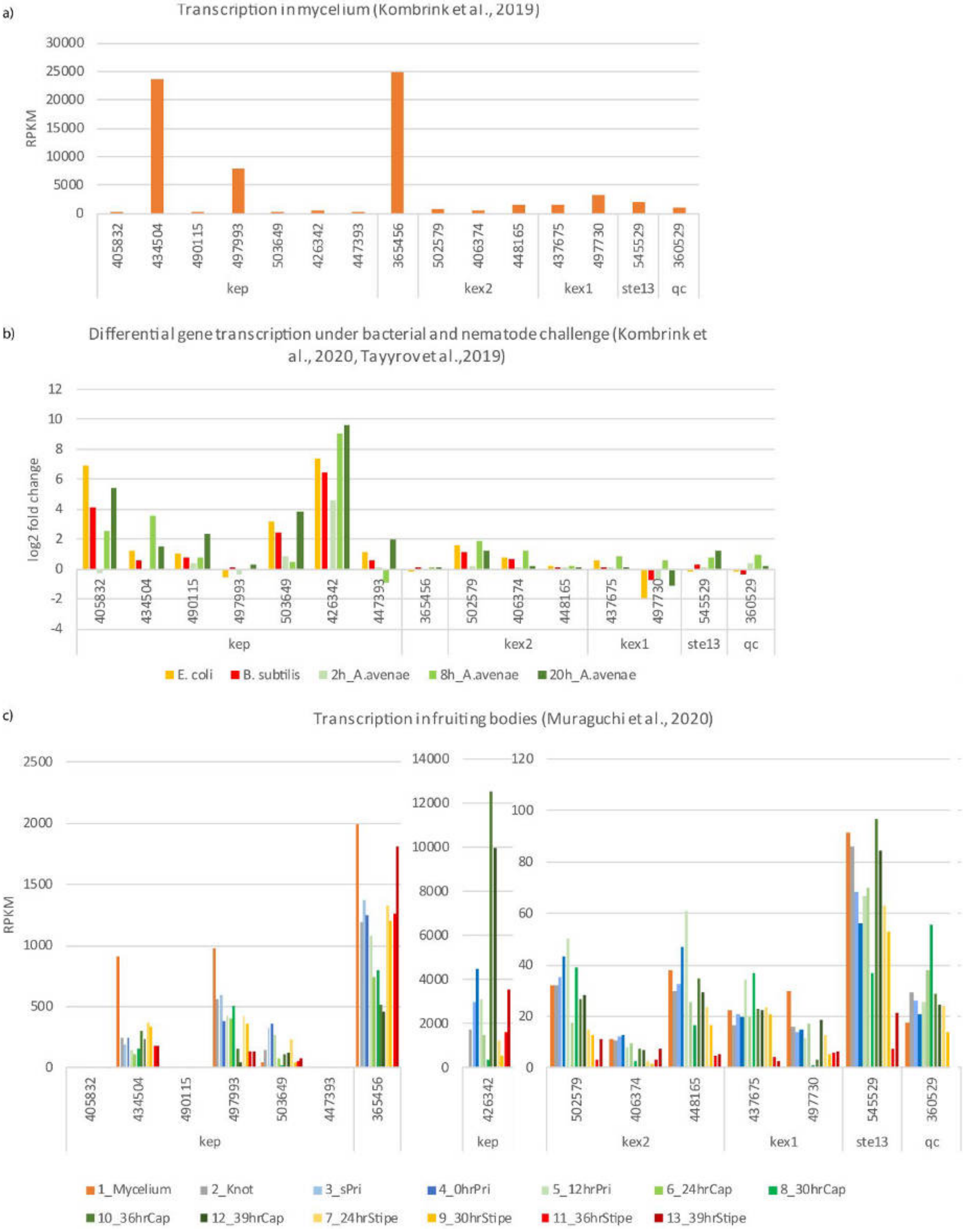

Figure S1: Gene transcription profiles of the *C. cinerea kep* genes, the gene 365456, *kex* genes, *ste13,* and a glutaminyl cyclase gene.

a) Gene transcription in the vegetative mycelium. The y-axis describes reads per kilobase of transcript per million reads mapped (RPKM). b) Differential gene transcription of *C. cinerea* challenged with the bacteria *E. coli* or *B. subtilis* or the nematode *A. avenae*. c) Gene transcription in fruiting body development. For visual clarity, *kep426342* and *kex2, kex1, ste13,* and glutaminyl cyclase *(qc)* genes are shown on different scales than the other genes. Samples harvested at 39 h are premature fruiting bodies. Raw data from Muraguchi et al. 2015; Kombrink et al. 2019; Tayyrov et al. 2019.

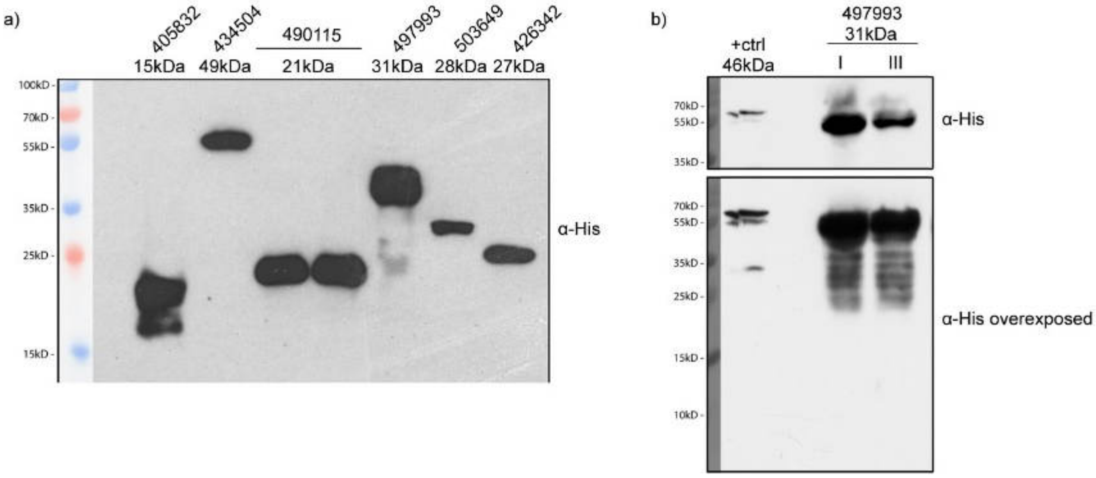

Figure S2: Verification of expression of *C. cinerea* KEPs in *P. pastoris*.

a) Immunoblot of whole cell extracts of *P. pastoris* expressing *C. cinerea* KEPs 405832, 434504, 490115, 497993, 503649, and 426342. Samples were run on a 12% SDS-PAGE, blotted on a nitrocellulose membrane and hybridized with anti-His antibodies. Detection of bands of the correct size confirmed the successful expression of the six proteins. b) Immunoblot of whole cell extracts of *P. pastoris* expressing *C. cinerea* KEP497993. Solubility of the protein was tested by preparing samples before (pellet I) and after (pellet III) high speed centrifugation at 16000 rcf for 30 min at 4°C.

S3

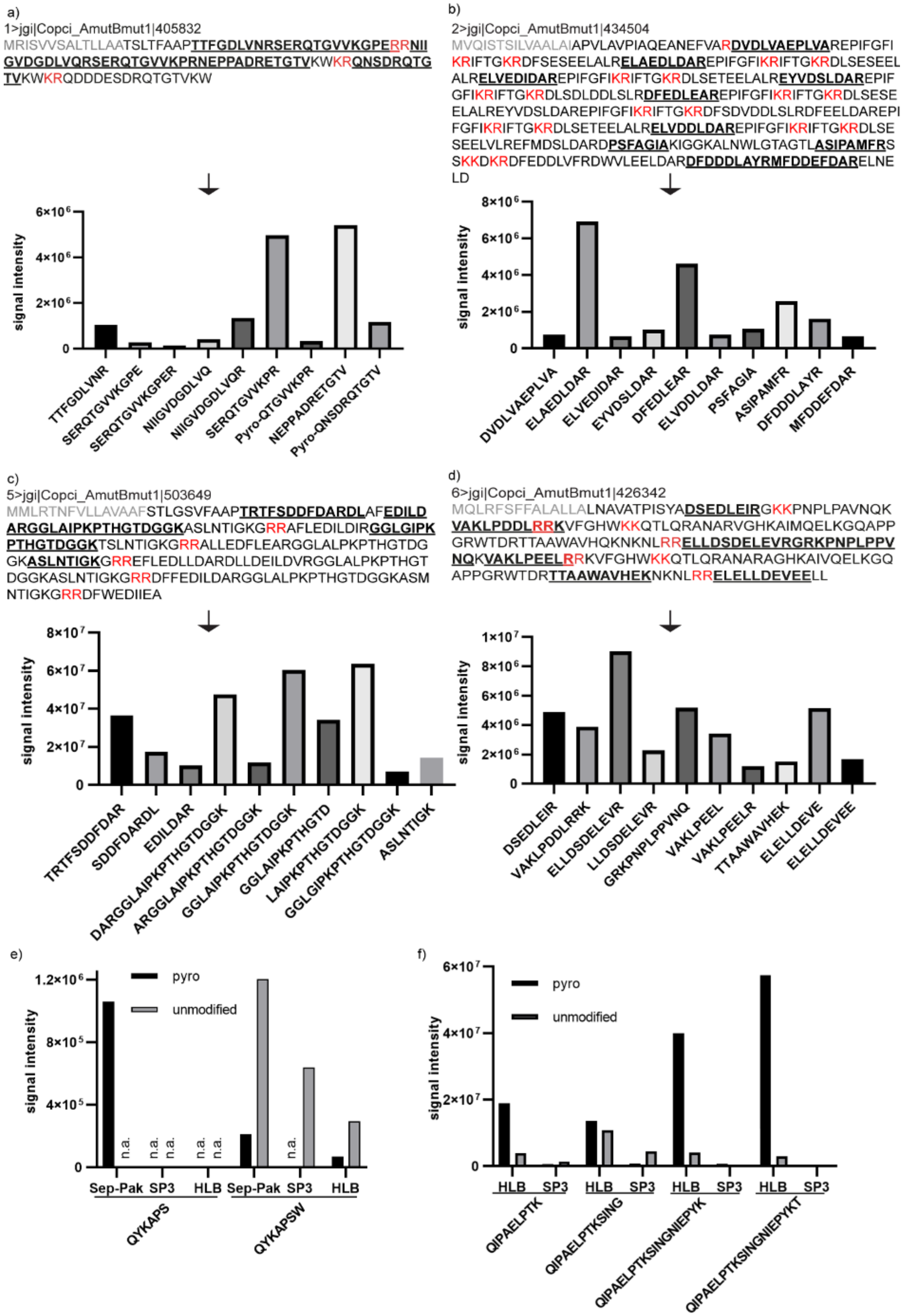
a)
1>jgi|Copci_AmutBmut1|405832
MRISVVSALTLLAATSLTFAAPTTFGDLVNRSERQTGVVKGPERRNII
GVDGDLVQRSERQTGVVKPRNEPPADRETGTVKWKRQNSDRQTG
TVKWKRQDDDESDRQTGTVKW
signal intensity
6×10^6
4×10^6
2×10^6
0
TTFGDLVNR
SERQTGVVKGPE
SERQTGVVKGPER
NIIGVDGDLVQ
NIIGVDGDLVQR
SERQTGVVKPR
Pyro-QTGVVKPR
NEPPADRETGTV
Pyro-QNSDRQTGTV
b)
2>jgi|Copci_AmutBmut1|434504
MVQISTSILVAALAIAPVLAVPIAQEANEFVARDVDLVAEPLVAREPIFGFI
KRIFTGKRDFSESEELALRELAEDLDAREPIFGFIKRIFTGKRDLSESEEL
ALRELVEDIDAREPIFGFIKRIFTGKRDLSETEELALREYVDSLDAREPIF
GFIKRIFTGKRDLSDLDDLSLRDFEDLEAREPIFGFIKRIFTGKRDLSESE
ELALREYVDSLDAREPIFGFIKRIFTGKRDFSDVDDLSLRDFEELDAREPI
FGFIKRIFTGKRDLSETEELALRELVDDLDAREPIFGFIKRIFTGKRDLSE
SEELVLREFMDSLDARDPSFAGIAKIGGKALNWLGTAGTLASIPAMFRS
SKKDKRDFEDDLVFRDWVLEELDARDFDDDLAYRMFDDEFDARELNE
LD
signal intensity
8×10^6
6×10^6
4×10^6
2×10^6
0
DVDLVAEPLVA
ELAEDLDAR
ELVEDIDAR
EYVDSLDAR
DFEDLEAR
ELVDDLDAR
PSFAGIA
ASIPAMFR
DFDDDLAYR
MFDDEFDAR
c)
5>jgi|Copci_AmutBmut1|503649
MMLRTNFVLLAVAAFSTLGSVFAAPTRTFSDDFDARDLAFEDILD
ARGGLAIPKPTHGTDGGKASLNTIGKGRRAFLEDILDIRGGLGIPK
PTHGTDGGKTSLNTIGKGRRALLEDFLEARGGLALPKPTHGTDG
GKASLNTIGKGRREFLEDLLDARDLLDEILDVRGGLALPKPTHGT
DGGKASLNTIGKGRRDFFEDILDARGGLALPKPTHGTDGGKASM
NTIGKGRRDFWEDIIEA
signal intensity
8×10^7
6×10^7
4×10^7
2×10^7
0
TRTFSDDFDAR
SDDFDARDL
EDILDAR
DARGGLAIPKPTHGTDGGK
ARGGLAIPKPTHGTDGGK
GGLAIPKPTHGTDGGK
GGLAIPKPTHGTD
LAIPKPTHGTDGGK
GGLGIPKPTHGTDGGK
ASLNTIGK
d)
6>jgi|Copci_AmutBmut1|426342
MQLRFSFFALALLALNAVATPISYADSEDLEIRGKKPNPLPAVNQK
VAKLPDDLRRKVFGHWKKQTLQRANARVGHKAIMQELKGQAPP
GRWTDRTTAAWAVHQKNKNLRRELLDSDELEVRGRKPNPLPPV
NQKVAKLPEELRRKVFGHWKKQTLQRANARAGHKAIVQELKGQ
APPGRWTDRTTAAWAVHEKNKNLRRELELLDEVEELL
signal intensity
1×10^7
8×10^6
6×10^6
4×10^6
2×10^6
0
DSEDLEIR
VAKLPDDLRRK
ELLDSDELEVR
LLDSDELEVR
GRKPNPLPPVNQ
VAKLPEEL
VAKLPEELR
TTAAWAVHEK
ELELLDEVE
ELELLDEVEE
e)
pyro
unmodified
signal intensity
1.2×10^6
8×10^5
4×10^5
0
n.a.
n.a.
n.a.
n.a.
n.a.
n.a.
Sep-Pak
SP3
HLB
Sep-Pak
SP3
HLB
QYKAPS
QYKAPSW
f)
pyro
unmodified
signal intensity
6×10^7
4×10^7
2×10^7
0
HLB
SP3
HLB
SP3
HLB
SP3
HLB
SP3
QIPAELPTK
QIPAELPTKSING
QIPAELPTKSINGNIEPYK
QIPAELPTKSINGNIEPYKT

Figure S3: KEP-derived peptides can be detected in the culture supernatant of *Pichia pastoris* upon expression of *C. cinerea* KEPs.

a-d) Peptides derived from the expression of *C. cinerea* KEPs 405832, 434504, 503649, and 426342 in *P. pastoris.* Given are the protein sequences with KEX2 cleavage sites labeled red and signal sequences labeled grey. The 10 peptides that were measured with the highest mass spectrometry signal intensities in the supernatant are indicated in the graphs. Their sequences are indicated in bold and underlined in the protein sequence. e-f) Comparison of pyroglutamated and unmodified KEP-derived peptides across different extraction methods in *P. pastoris* heterologous expressions**.** Signal intensities of a selection of peptides that were extracted using the solid phase extraction methods Sep-Pak, HLB, and SP3 from the *P. pastoris* expression of *C. cinerea* KEP 490115 (left) and KEP 497993 (right) are compared.

S4a)

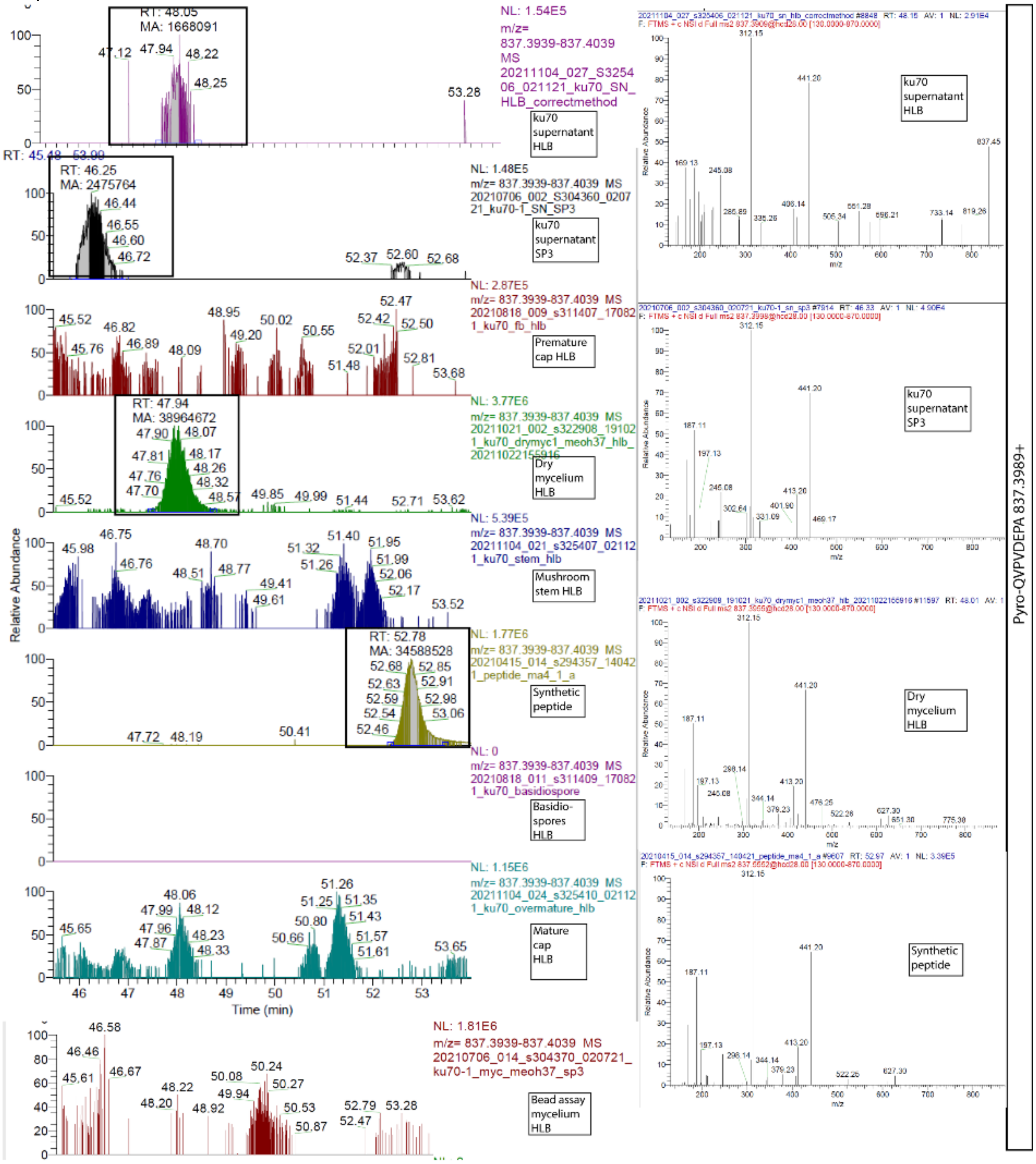

Pyro-QVPVDEPA	idotp	dotp
Supernatant	0.99	0.79
Dry mycelium	0.92	0.83

S4b)

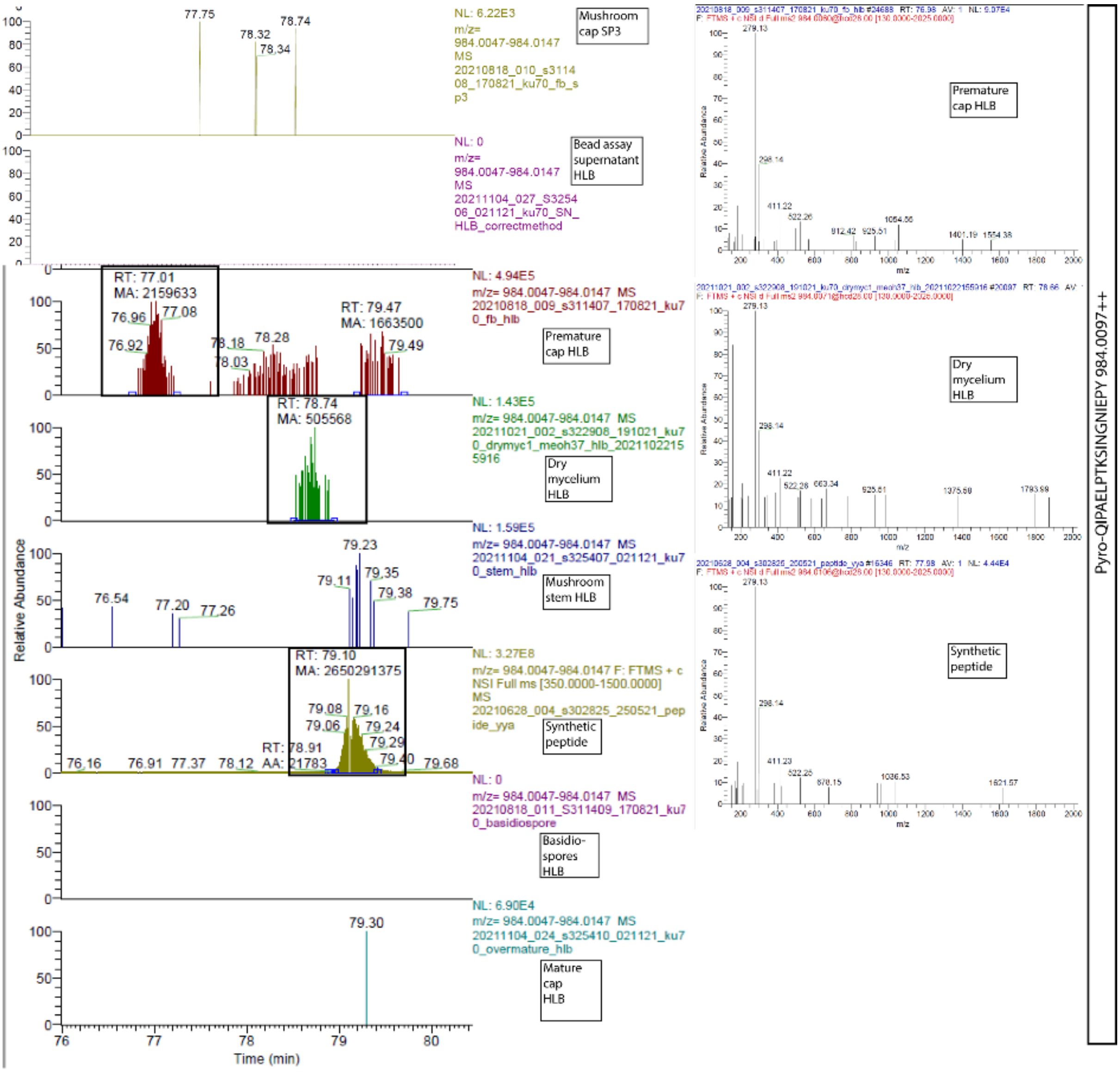

Pyro-QIPAELPTKSINGNIEPY	idotp	dotp
Dry mycelium	0.9	0.82
Mushroom cap	0.94	0.91

S4c)

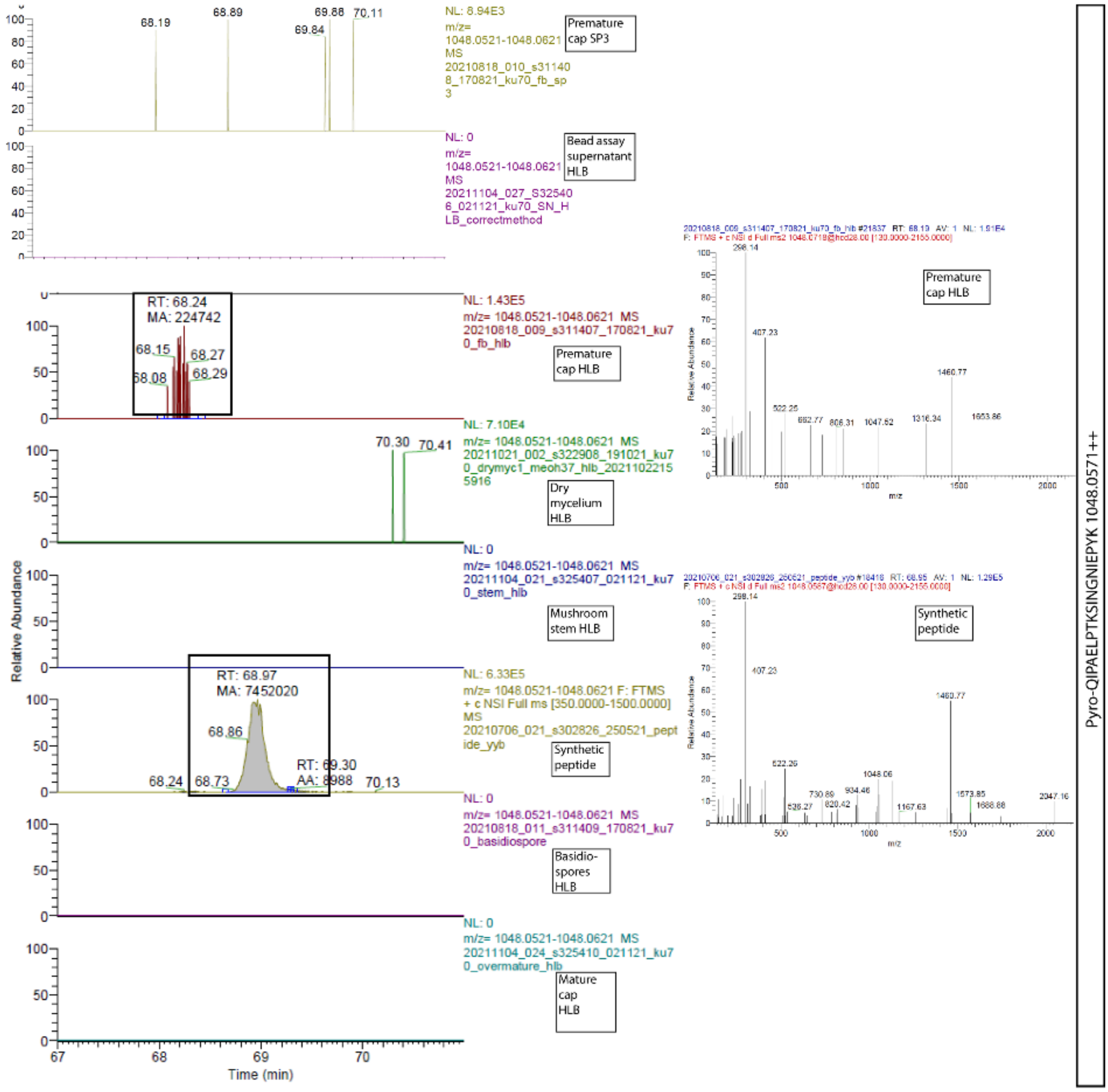

Pyro-QIPAELPTKSINGNIEPYK	idotp	dotp
Mushroom cap	0.86	0.91

S4d)

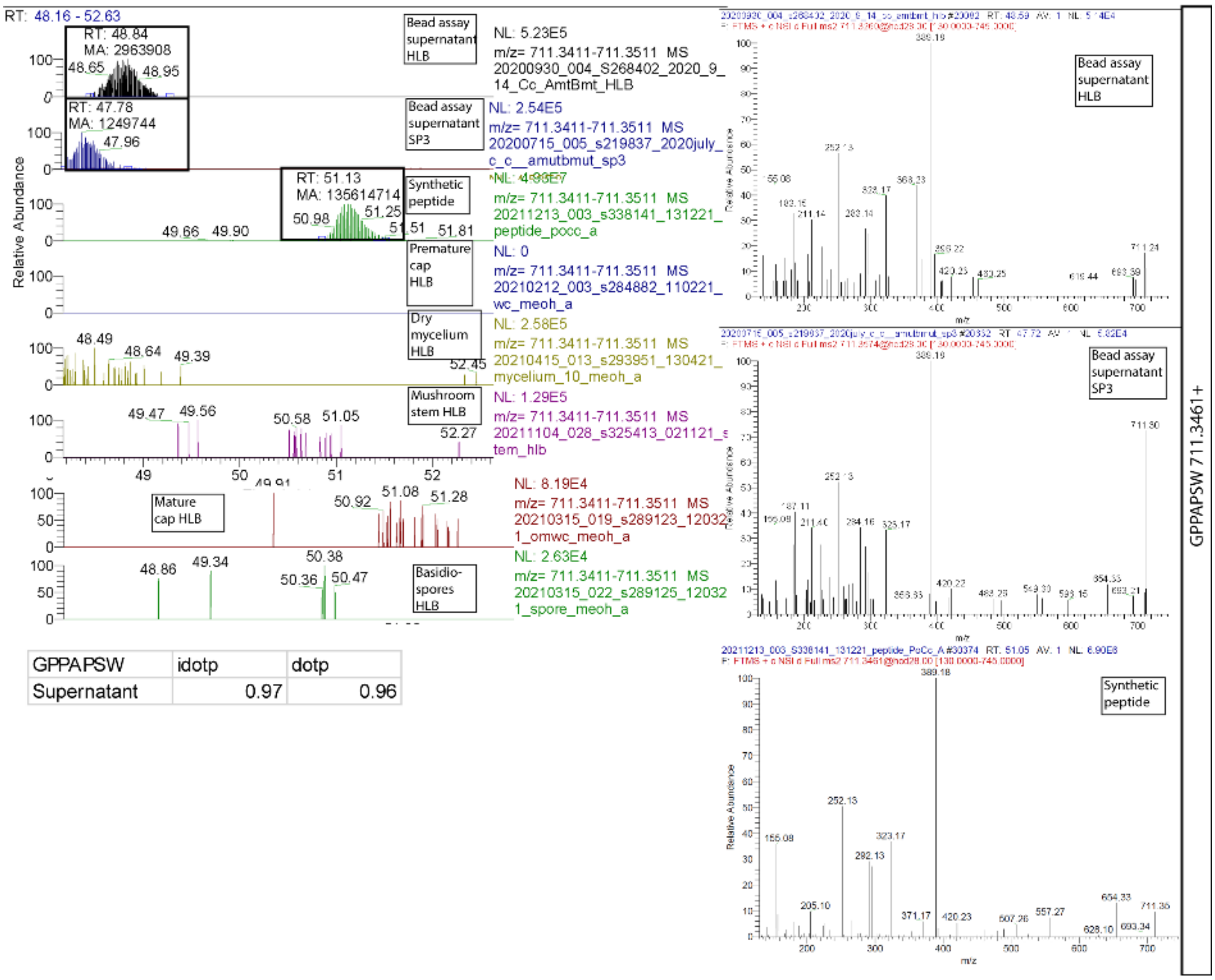

GPPAPSW	idotp	dotp
Supernatant	0.97	0.96

S4e)

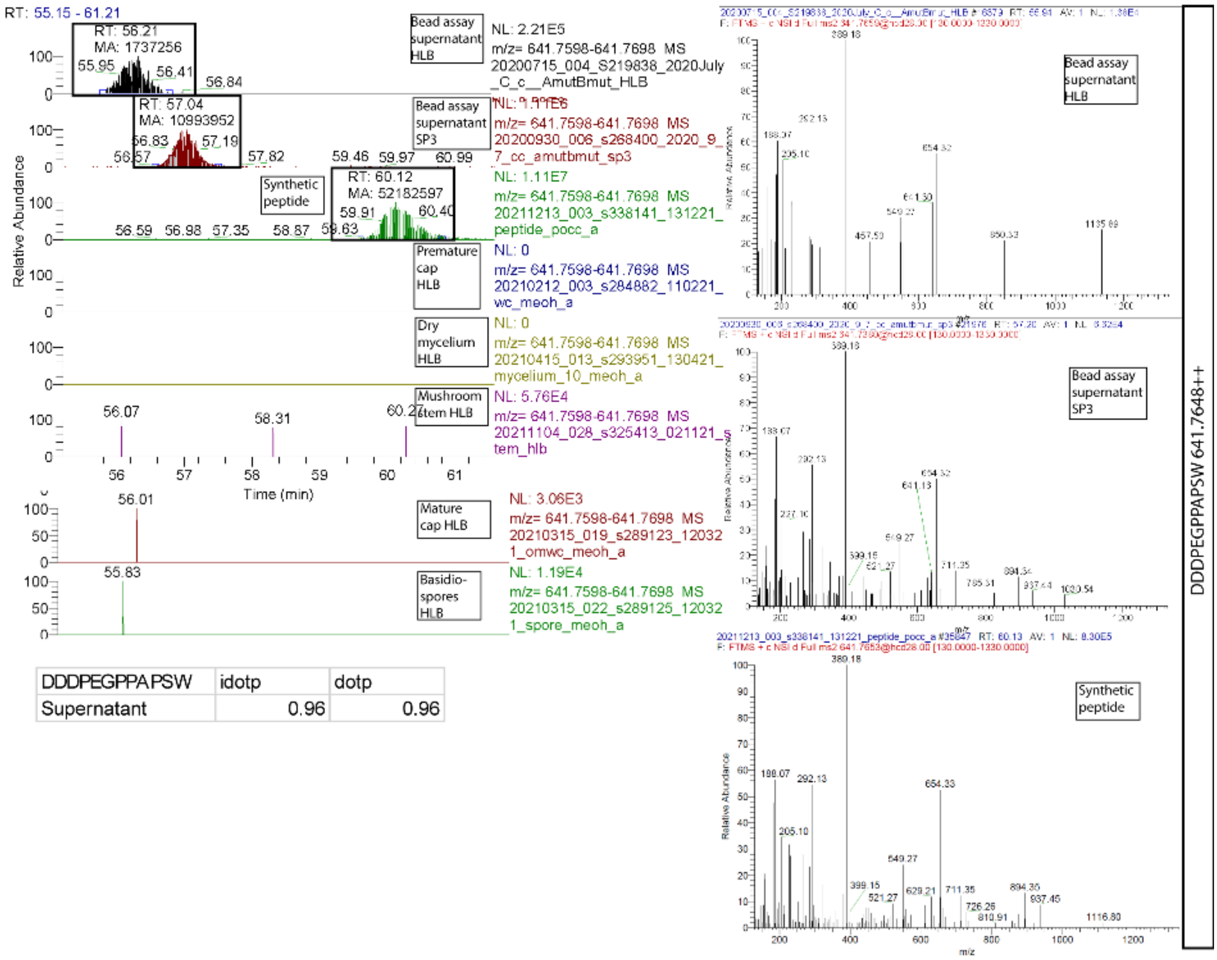

DDDPEGPPAPSW	idotp	dotp
Supernatant	0.96	0.96

S4f)

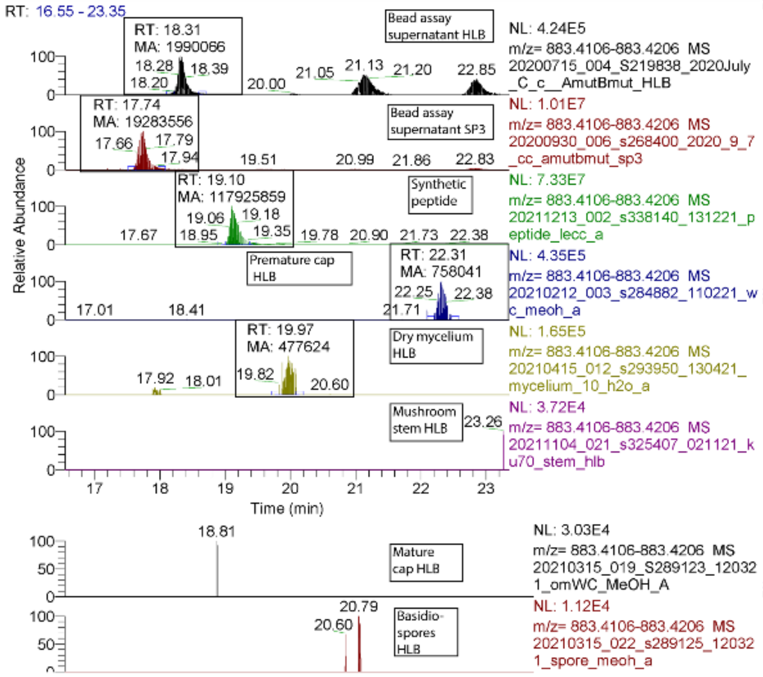

Pyro-QSEPKPTN	idotp	dotp
Supernatant	0.96	0.94
Dry mycelium	0.88	0.84
Mushroom cap	0.86	0.78

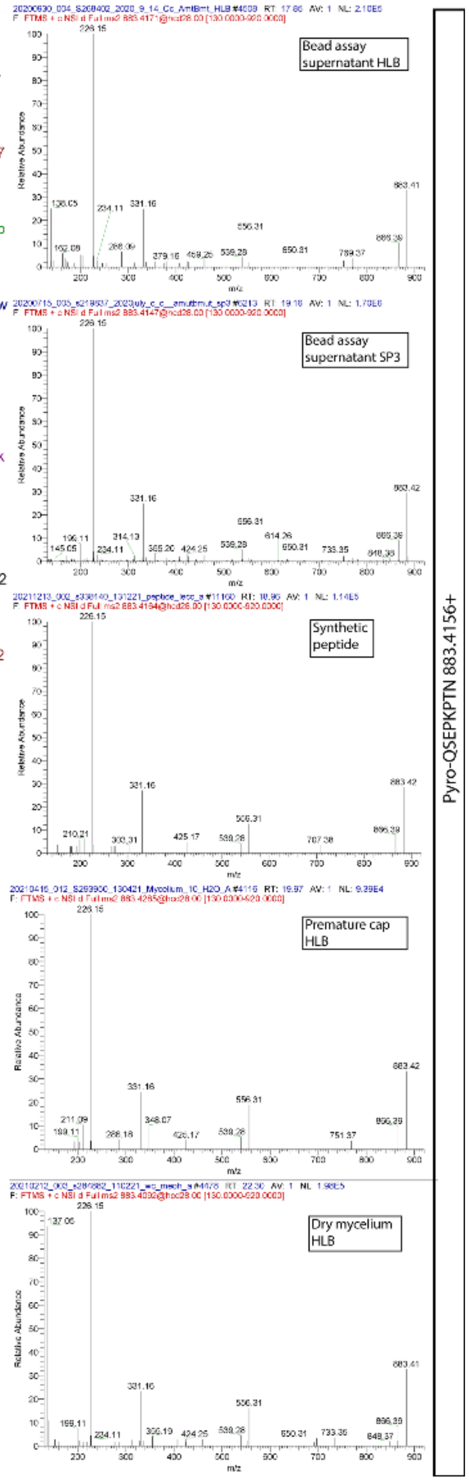

S4g)

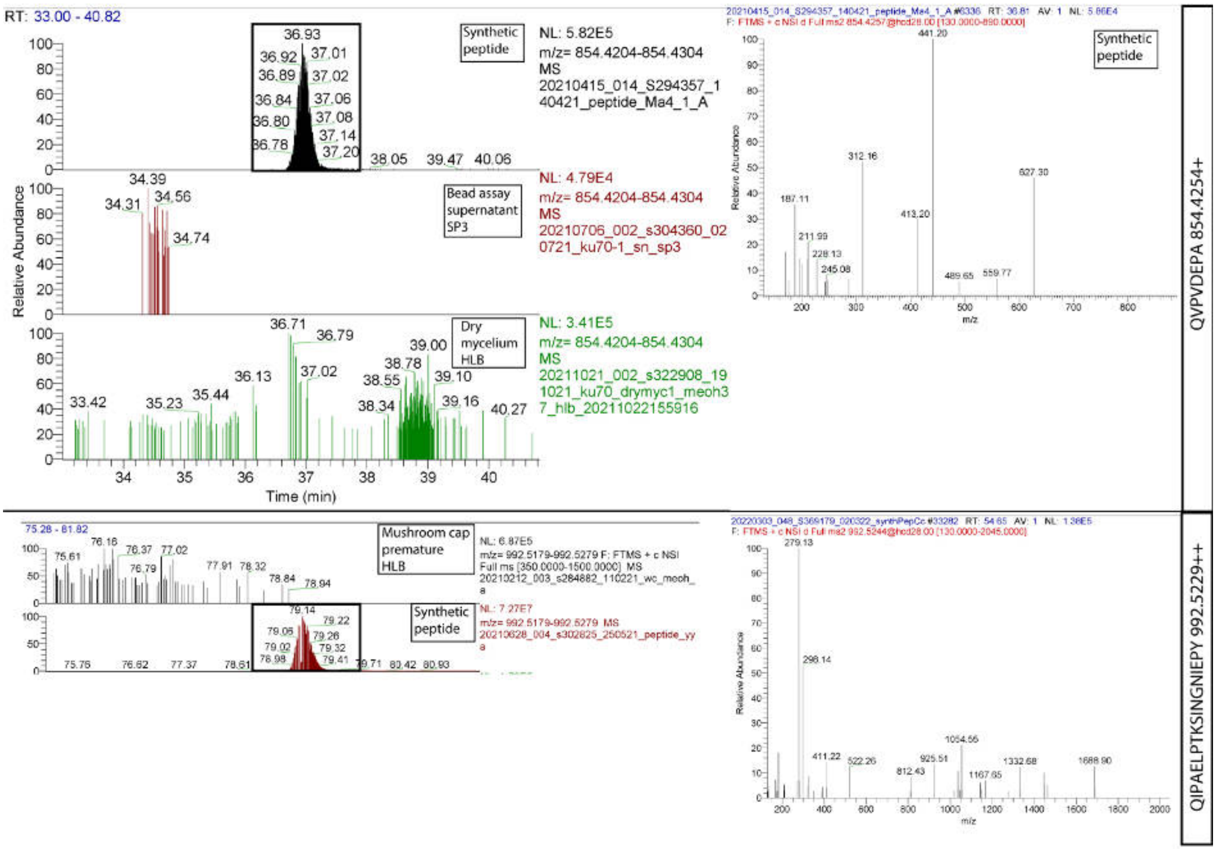

Figure S4: Extracted ion chromatograms and MS/MS spectra of peptides in C. cinerea tissues.
Panels a) to f) depict the extracted ion chromatograms (EIC, left) and the MS/MS spectra (right) with the retention times (RT) and the peak areas (MA) of endogenous peptides from *C. cinerea* samples or synthetic peptides that are used as gold standards. The isotope dot product (idot) and dot product (dotp) values, determined using the software Skyline 20.2.0.343 (MacCoss Lab Software, USA), are indicated as an additional means to compare similarity between the spectra. Samples were extracted using the solid phase extraction cartridge HLB or SP3 magnetic beads. Panels a) to f) each show the presence of one peptide in a selection of *C. cinerea* tissue samples. The peptides are a) pyro-QVPVDEPA, b) pyro-QIPAELPTKSINGNIEPY, c) pyro-QIPAELPTKSINGNIEPYK, d) GPPAPSW, e) DDDPEGPPAPSW, f) pyro-QSEPKPTN, and g) unmodified QVPVDEPA and QIPAELPTKSINGNIEPY.

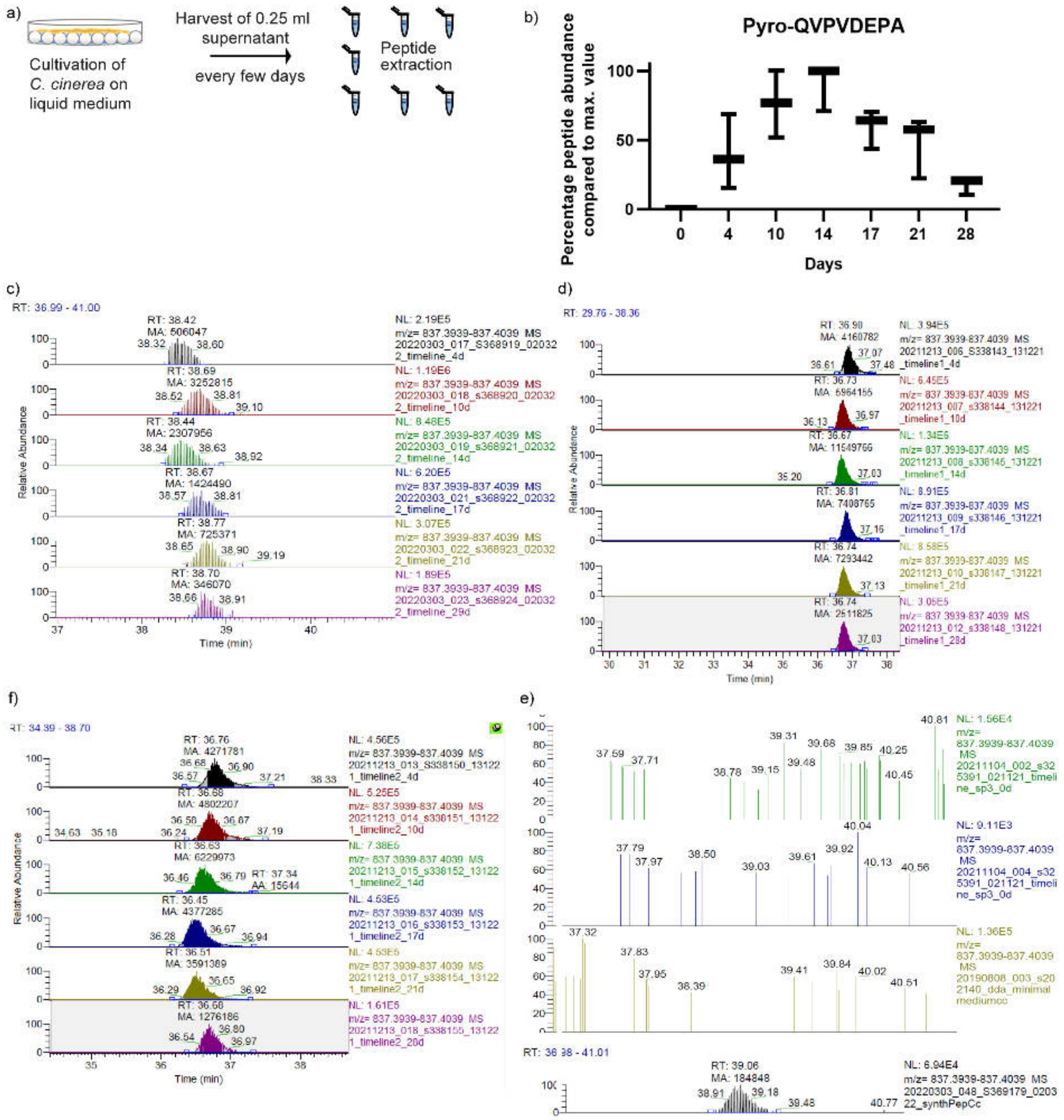

Figure S5: Presence of the KEP-derived peptide pyro-QVPVDEPA in time-course sampling of *C. cinerea* culture supernatant.

a) Workflow of the experiment. A triplicate of *C. cinerea Δku70* bead assays were prepared where the fungus grows on glass beads covered with liquid minimal medium. The supernatant was sampled every few days. b) Time-course of peptide abundance over 28 days. The peptide peak areas of the extracted ion chromatograms of each time point were determined, and the maximal value of each of three data sets was set as 100%. The rest of the values were then compared to this maximum. The single bands represent the median value of three biological replicates, the whiskers represent the 10-90% percentile range. The subpanels c) to f) show the extracted ion chromatograms of three independent data sets, e) shows the extracted ion chromatograms of the 0 d minimal medium samples and the synthetic peptide.

S6a)

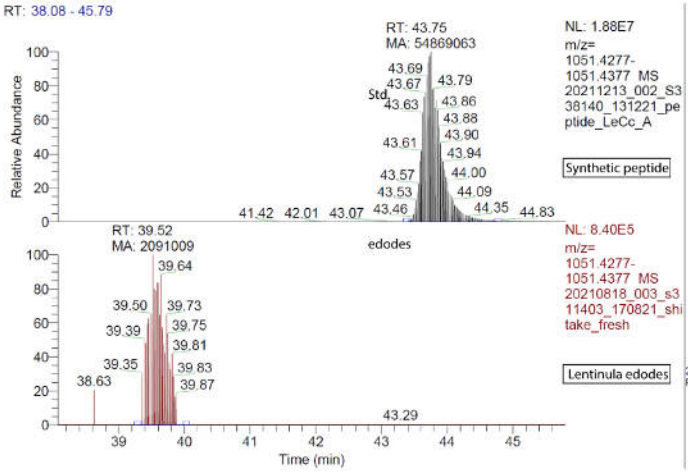

SGTGEASAADW	idotp	dotp
L. edodes	0.96	0.92

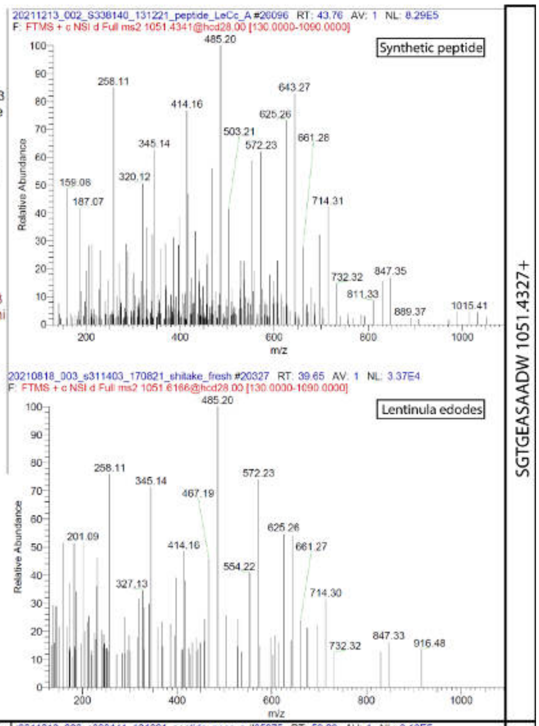

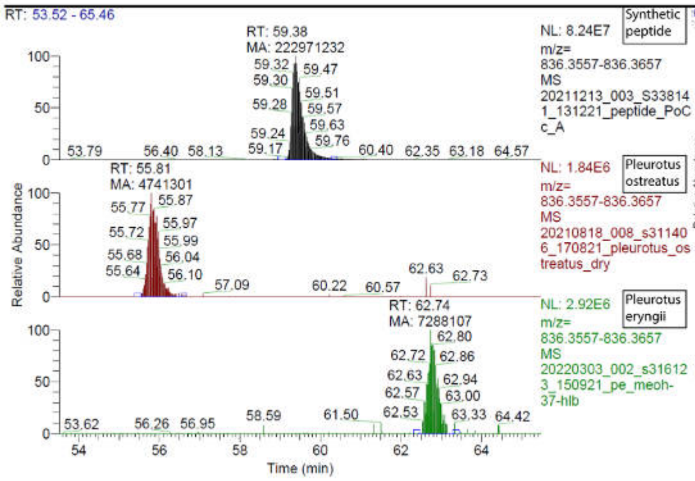

MSGVAADW	idotp	dotp
P. ostreatus	0.97	0.97
P. eryngii	0.91	0.97

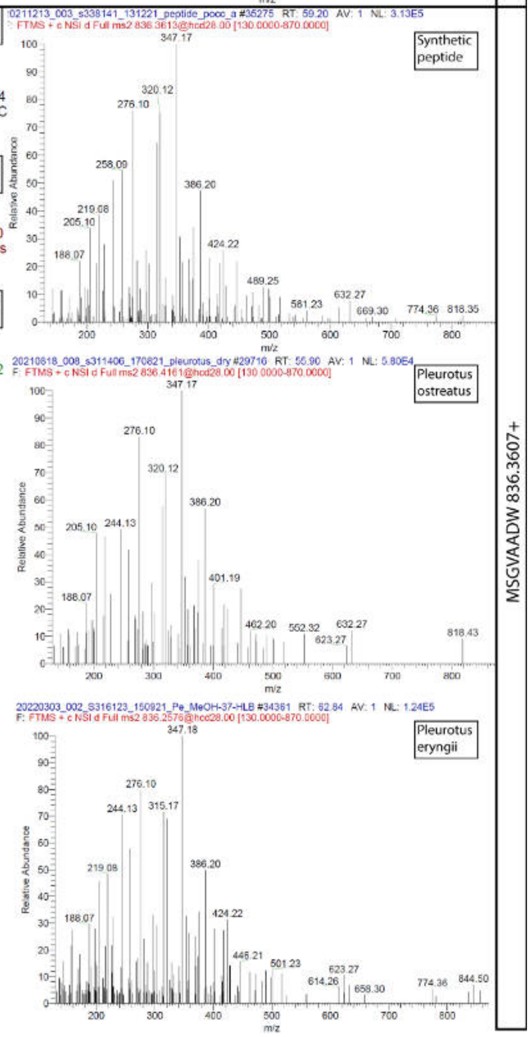

S6b)

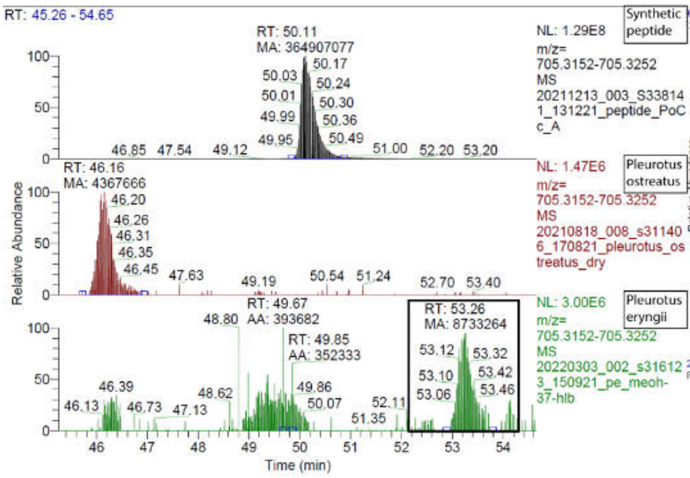

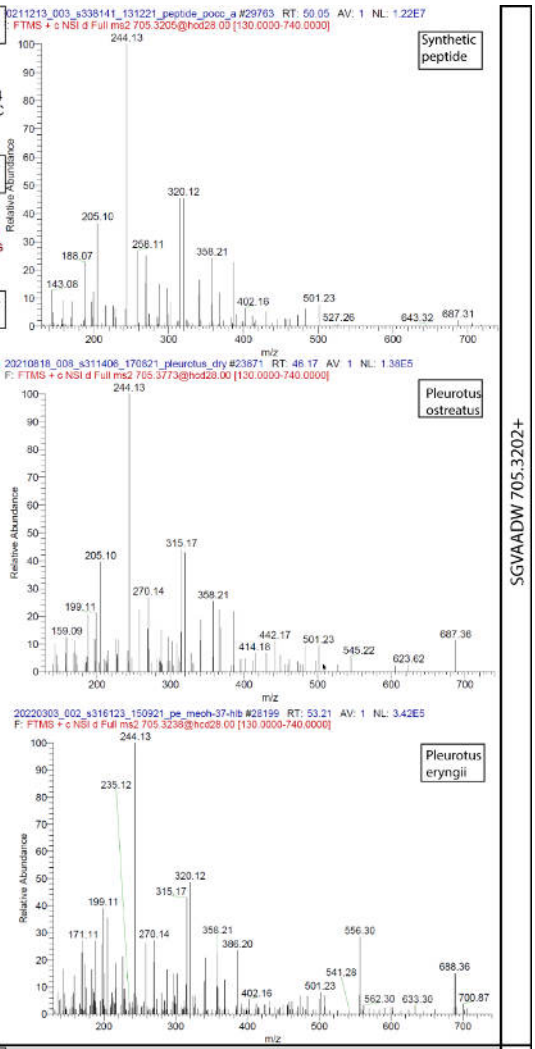

SGVAADW	idotp	dotp
P. ostreatus	0.95	0.97
P. eryngii	0.93	0.97

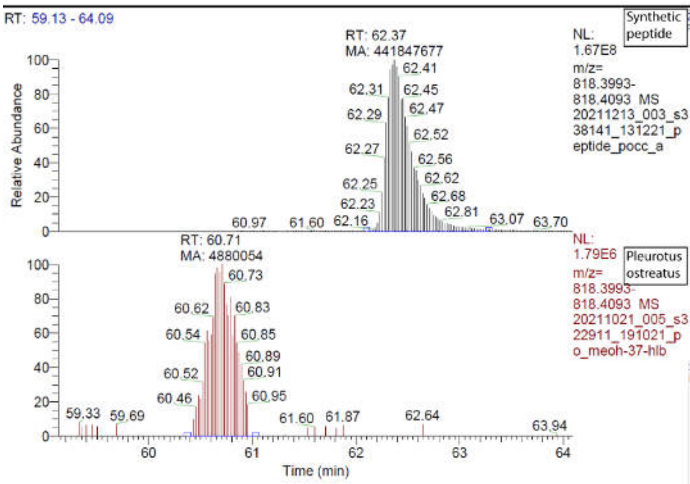

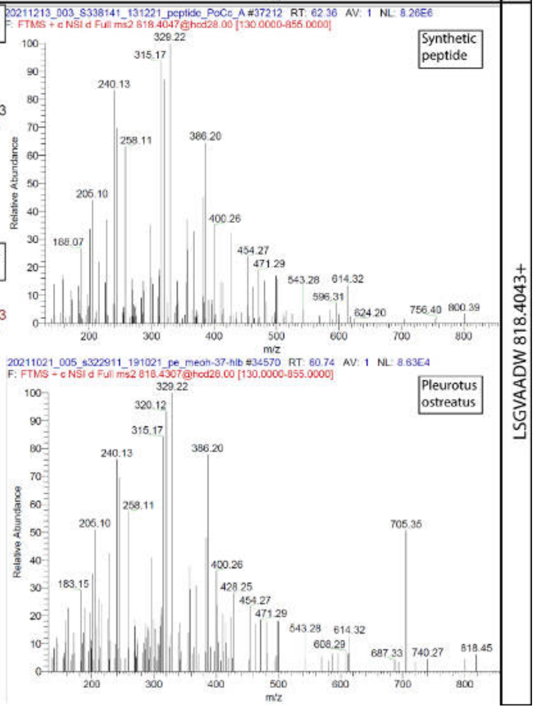

LSGVAADW	idotp	dotp
P. ostreatus	0.9	0.95

Figure S6: Extracted ion chromatograms and MS/MS spectra of peptides in *L. edodes, P. ostreatus,* and *P. eryngii* fruiting bodies.

Panels a) and b) depict the extracted ion chromatograms (left) and the MS/MS spectra (right) with the retention times and the peak areas of endogenous peptides *from L. edodes, P. ostreatus,* and *P. eryngii* fruiting body samples or synthetic peptides that are used as gold standards. The isotope dot product (idot) and dot product (dotp) values, determined using the software Skyline, are given as an additional means to compare similarity between the spectra. The peptides are a) SGTGEASAADW and MSGVAADW, and b) SGVAADW and LSGVAADW.

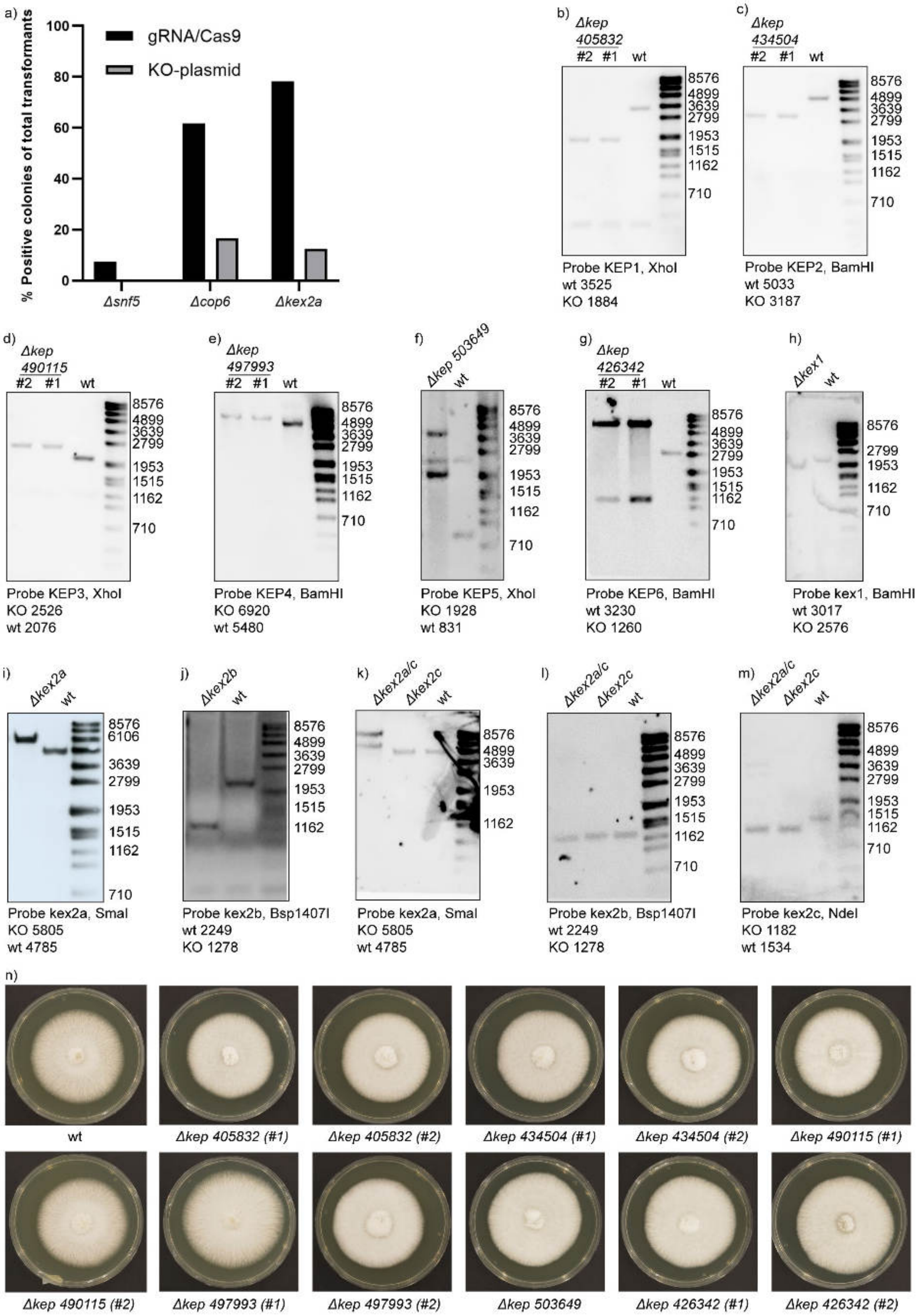

Figure S7: Establishment of *kex* and *kep* knockout strains in *C. cinerea*.

All knockouts are based on the strain AmutBmut *Δku70* that is labeled as wildtype. a) Comparison of the knockout efficiency for three genes. The efficiency of a gRNA/Cas9-guided knockout protocol was

assessed in comparison to a traditional non-CRISPR knockout protocol for the genes *snf5* (365798), *cop6* (394772), and *kex2a* (502579). After transformation, picked mycelial colonies were tested in PCR reactions for the presence of a band indicating successful genomic mutation. The numbers of tested colonies were 67 and 56 for *Δsnf5*, 60 and 24 for *Δcop6*, and 55 and 16 for *Δkex2*, for gRNA/Cas9 and KO-plasmid alone, respectively. Given is the percentage of colonies whose tests were positive. The genes for *cop6* and *kex2a* were fully replaced with a *pab1* selection marker (4) using two different gRNAs that cleaved at the 5' and 3' end of the gene, while the *snf5* gene was simply disrupted with the *pab1* gene using a single gRNA according to the knockout strategy by Ando et al. 2013. Subpanels b) to m) depict the confirmation of gene deletions by Southern blot analysis. b) to g) show blots of *kep* knockouts, h) to m) *kex* knockouts. The bands in h) displayed a mass shift due to the use of GelRed. n) Mycelial growth of *kep* knockout strains on solid YMG agar medium. Shown are plates after three days of growth at 37°C.

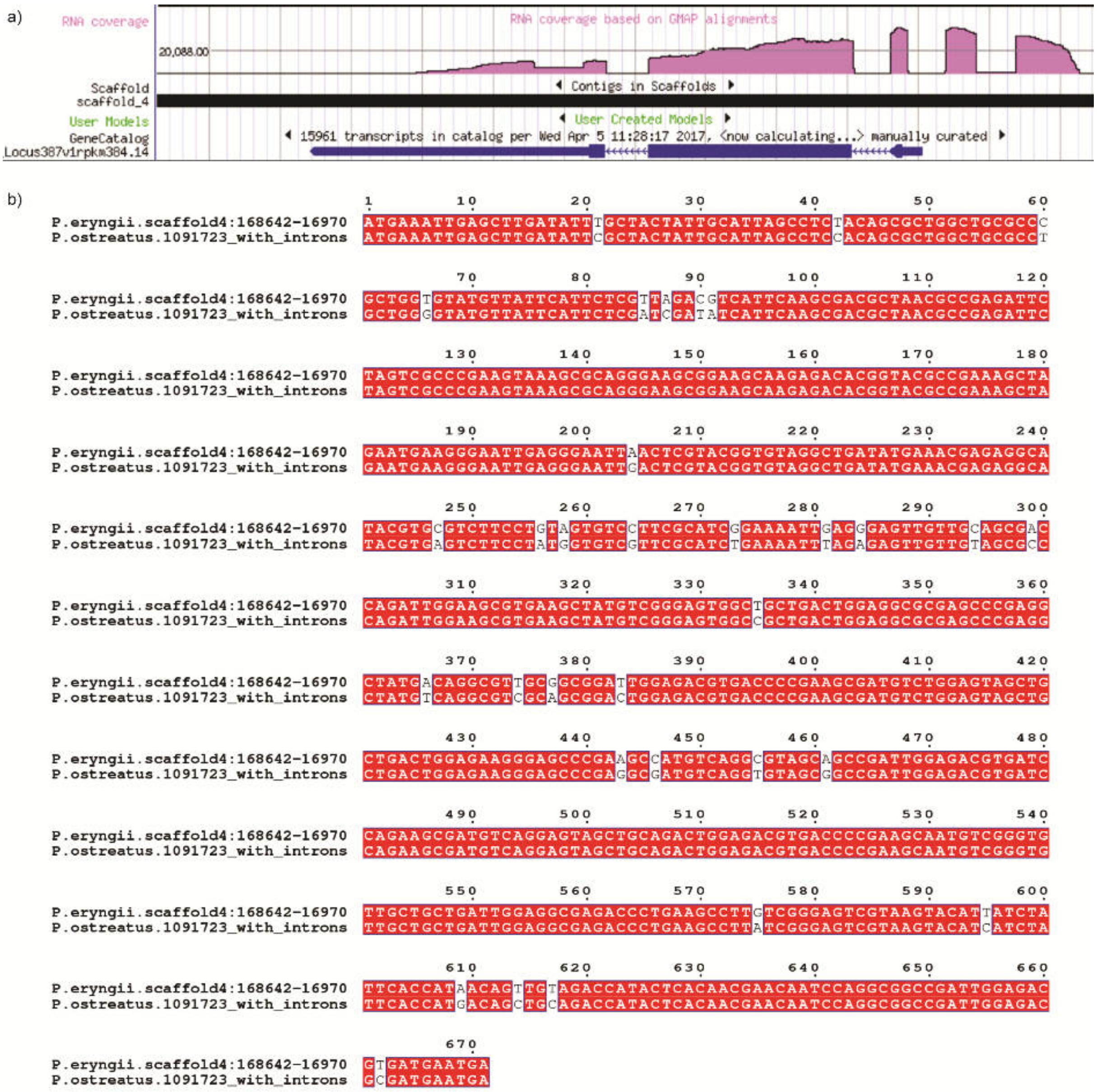

Figure S8: Analyis of gene annotation for the gene 439342 from *P. eryngii.*

a) Genomic location and RNA coverage of the gene 439342. The gene is located on scaffold_4:168642-169504 on the negative-sense strand, as shown in the JGI Mycocosm genome viewer (https://mycocosm.jgi.doe.gov). b) Alignment of the nucleotide sequence of the two KEP homologs 1091723 and 439342 from *P. ostreatus* and *P. eryngii*, respectively. Shown is the gene coding for KEP 1091723, including introns, and the genomic locus 168642-169701, scaffold 4, of *P. eryngii*. The annotated gene of *P. eryngii* starts at the Start codon ATG at residue 226.

S9a)

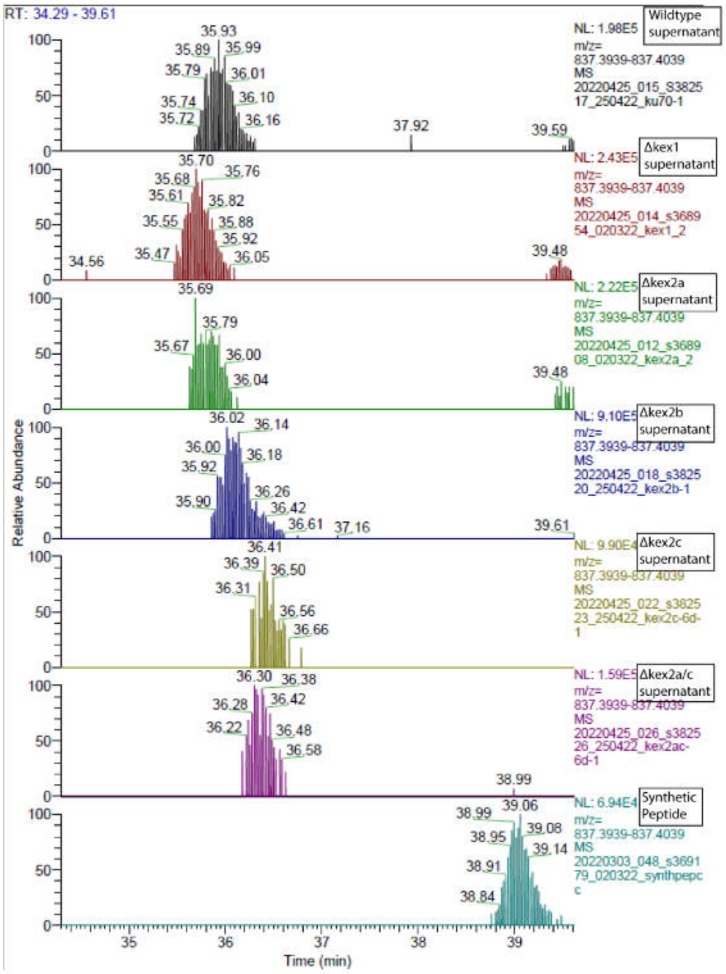

Pyro-QVPVDEPA	idotp	dotp
Wildtype	0.99	0.79
Δkex1	0.93	0.78
Δkex2a	0.99	0.85
Δkex2b	0.94	0.87
Δkex2c	0.95	0.78
Δkex2a/c	0.82	0.85

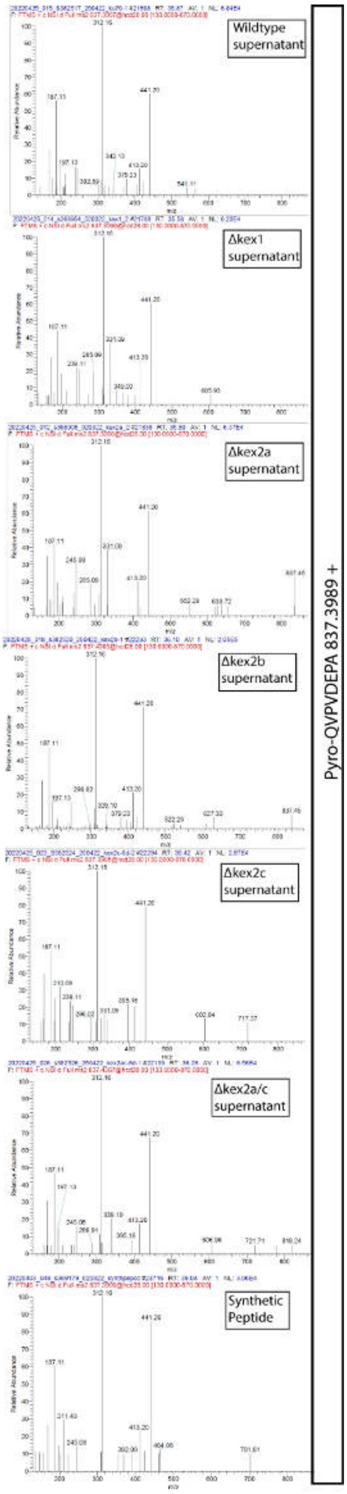

S9b)

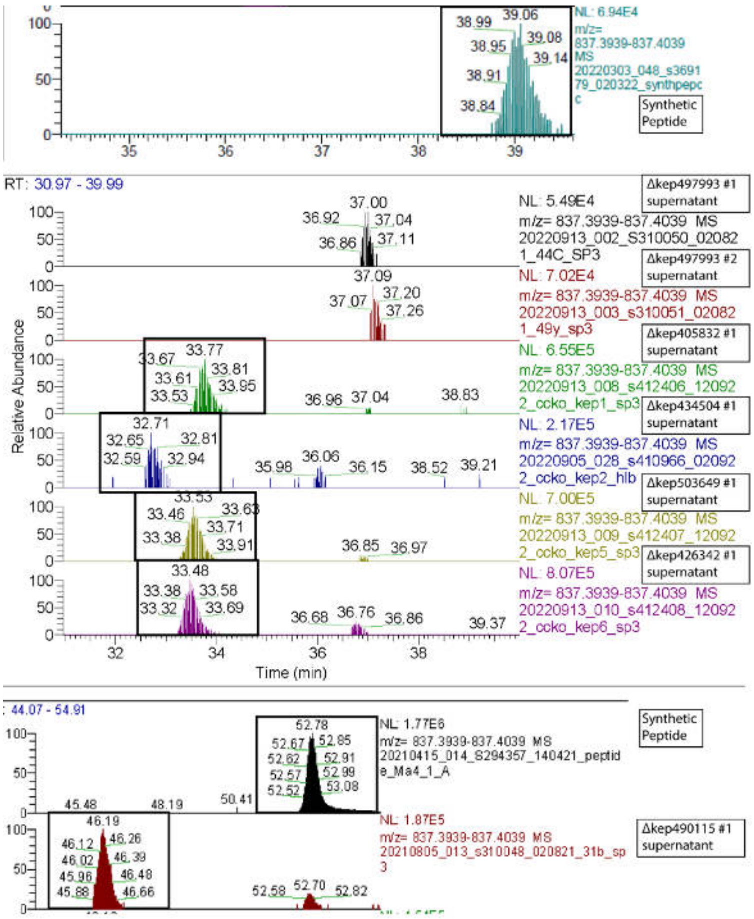

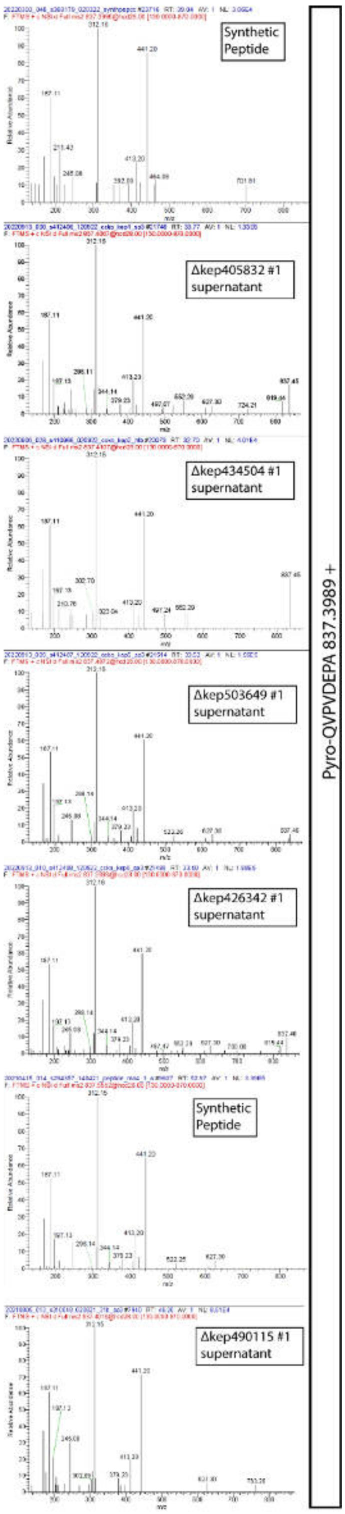

Pyro-QVPVDEPA	idotp	dotp
Δkep405832	0.95	0.85
Δkep434504	0.8	0.72
Δkep490115	0.96	0.83
Δkep503649	0.94	0.84
Δkep426342	0.97	0.84

S9c)

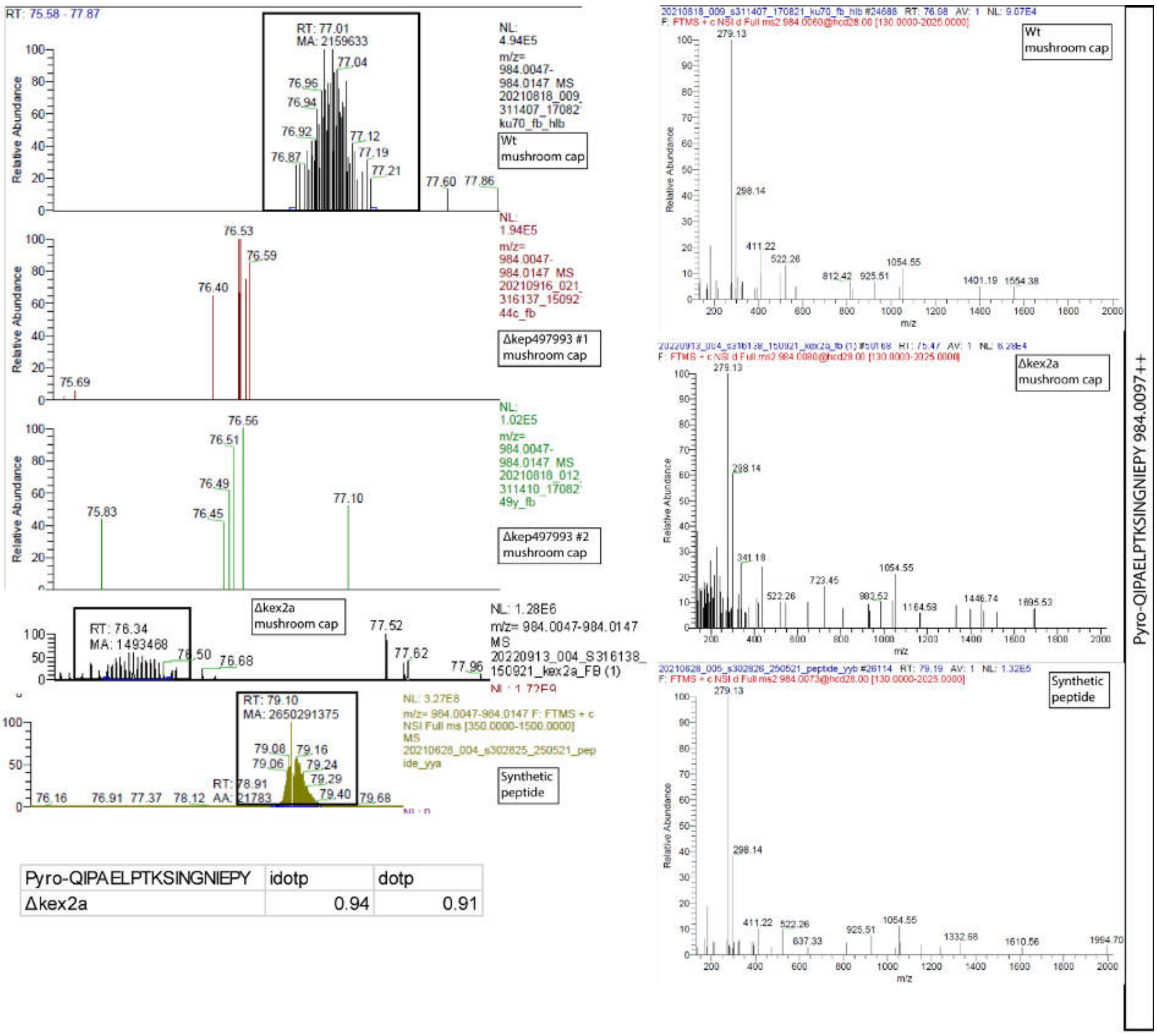

Pyro-QIPAELPTKSINGNIEPY	idotp	dotp
Δkex2a	0.94	0.91

S9d)

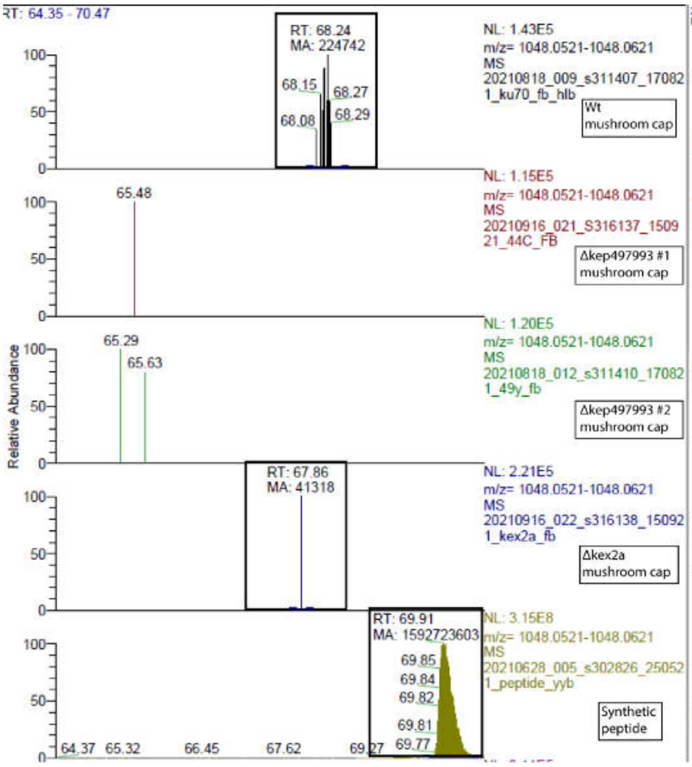

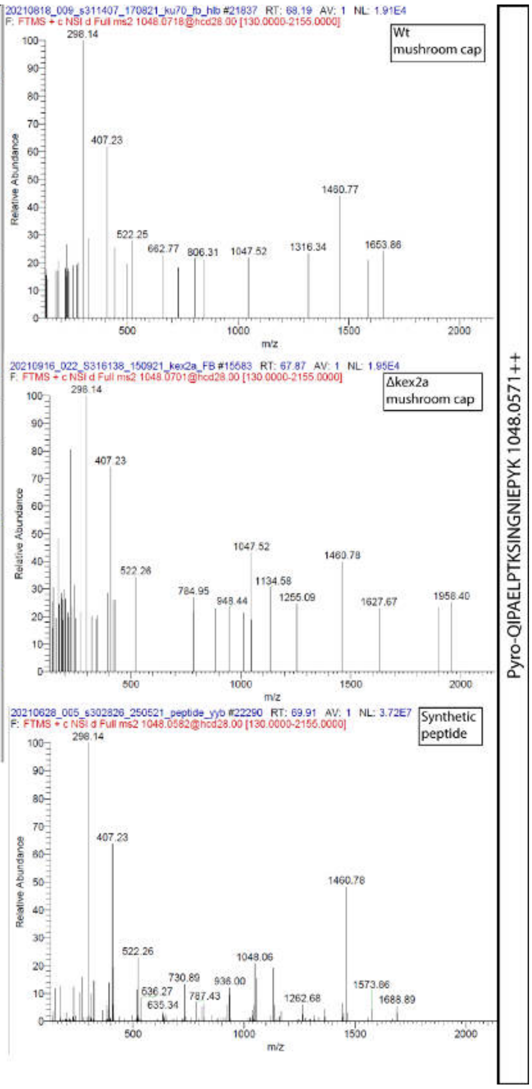

Pyro-QIPAELPTKSINGNIEPYK	idotp	dotp
Δkex2a	0.87	0.76

S9e)

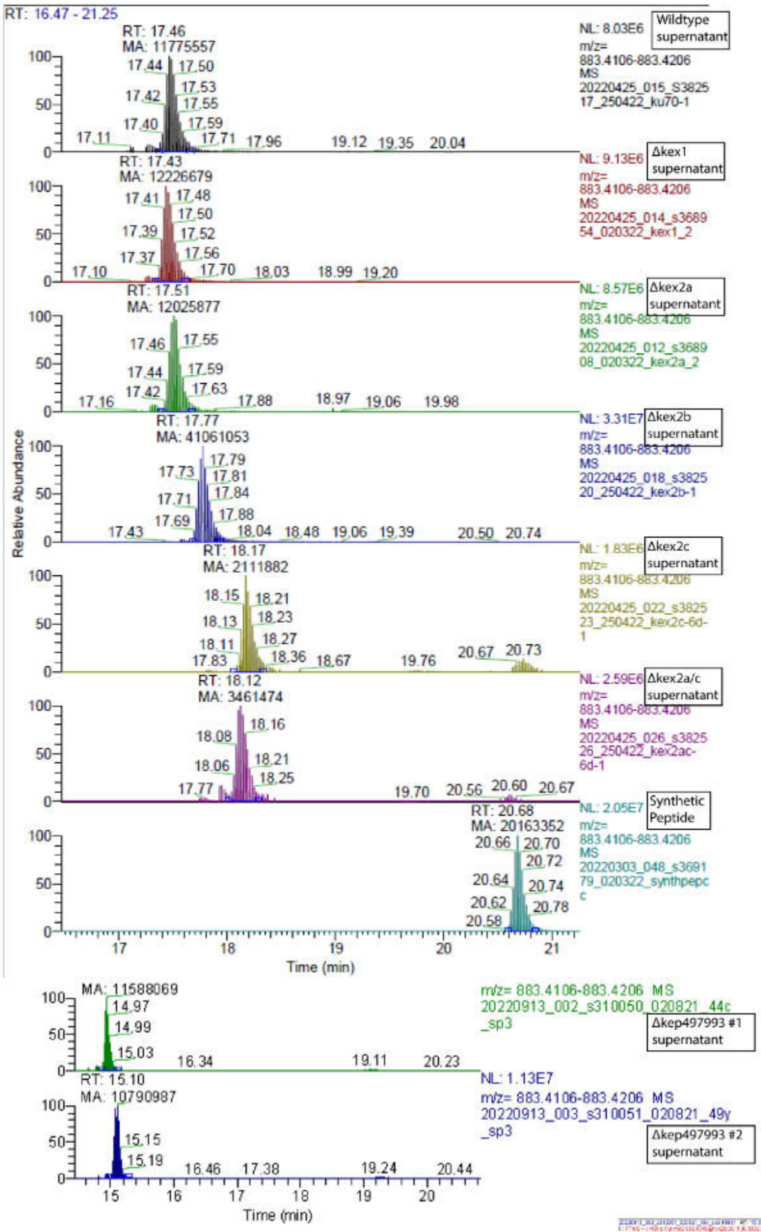

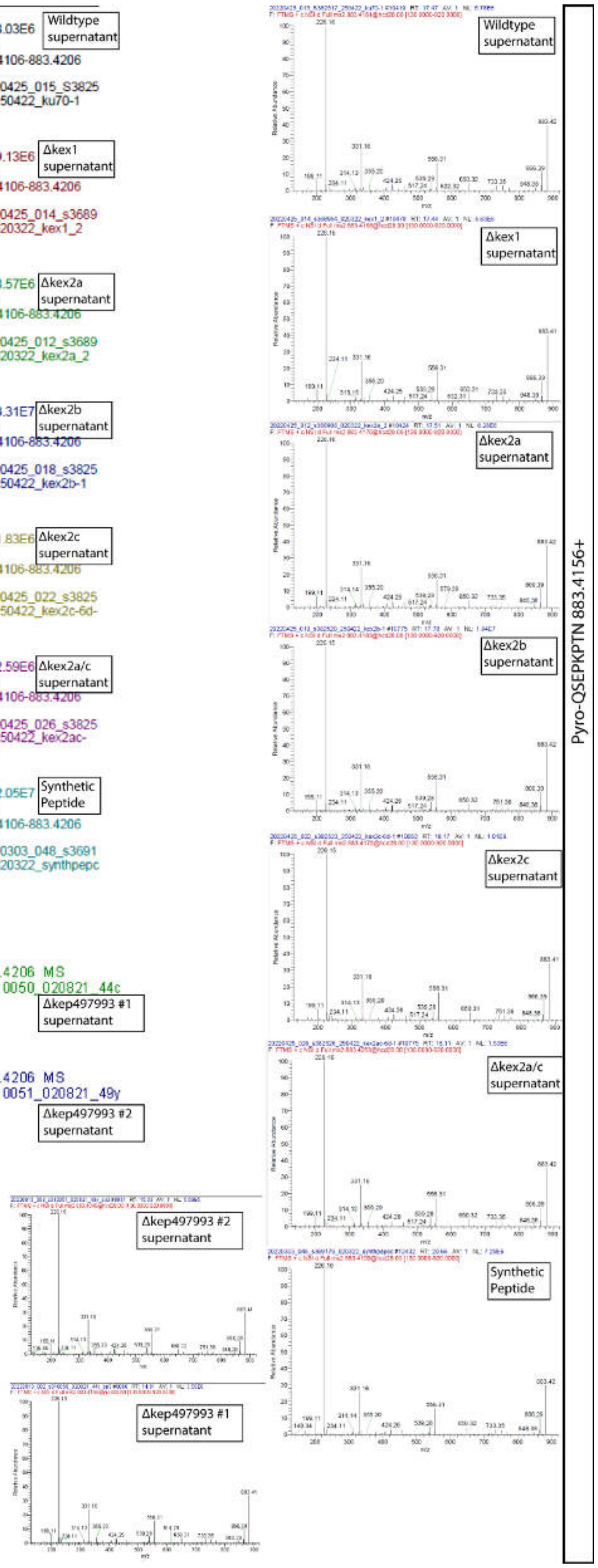

Pyro-QSEPKPTN	idotp	dotp
Δkex1	0.98	0.83
Δkex2a	0.98	0.84
Δkex2b	0.99	0.83
Δkex2c	0.99	0.83
Δkex2a/c	0.98	0.85
Δkep497993-1	0.98	0.84
Δkep497993-2	0.98	0.84

Figure S9: Extracted ion chromatograms and MS/MS spectra of peptides of all constructed *C. cinerea* knockout strains.

Panels a) to d) depict the extracted ion chromatograms (EIC, left) and the MS/MS spectra (right) with the retention times and the peak areas of endogenous peptides from *C. cinerea* samples or synthetic peptides that are used as gold standards. The isotope dot product (idot) and dot product (dotp) values, determined using the software Skyline, are given as an additional means to compare similarity

between the spectra. The peptides are a) pyro-QVPVDEPA in *kex* knockout strains, b) pyro-QVPVDEPA in *kep* knockout strains, c) pyro-QIPAELPTKSINGNIEPY, d) pyro-QIPAELPTKSINGNIEPYK, and e) pyro-QSEPKPTN.

S10a)

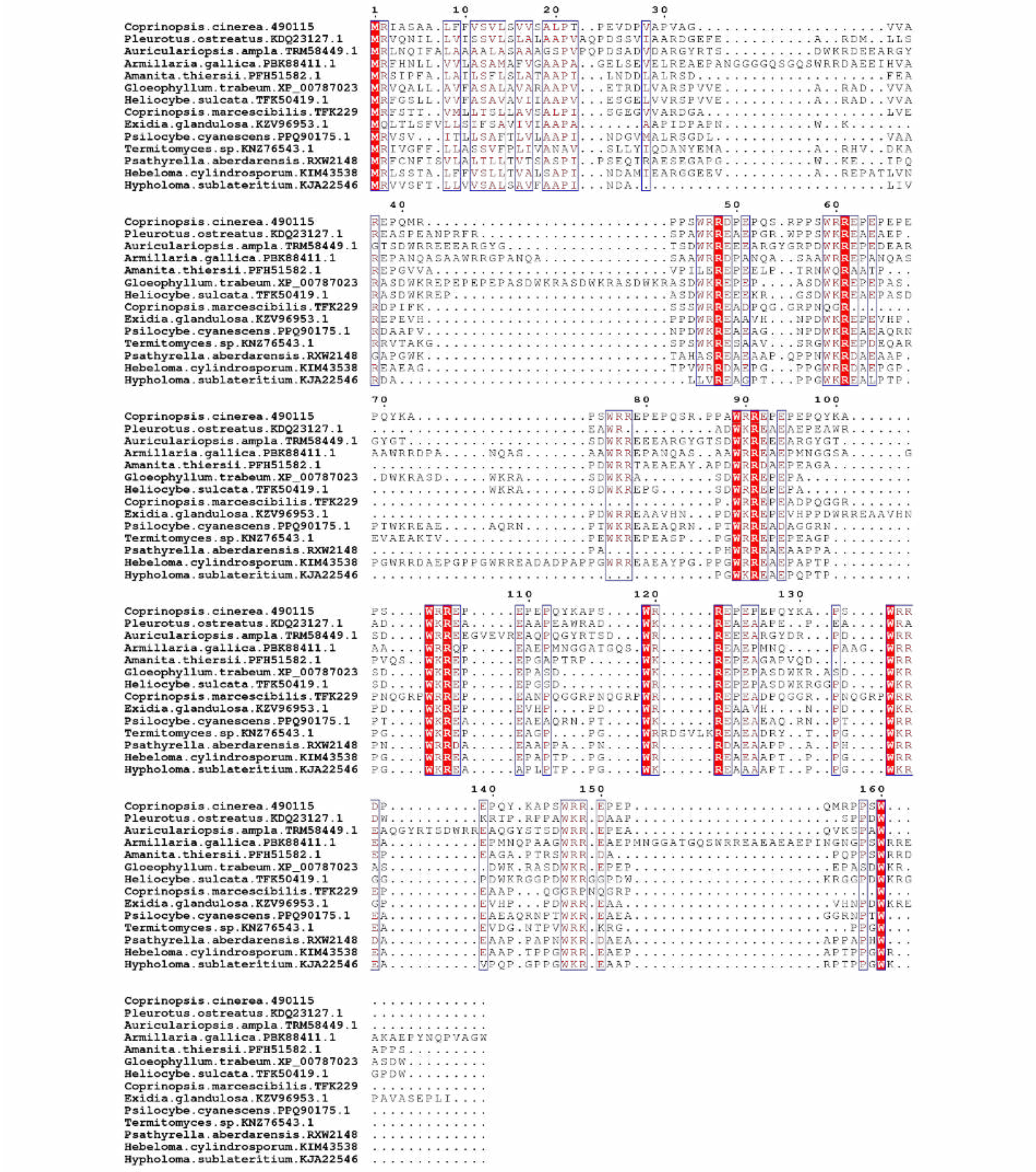
Coprinopsis.cinerea.490115
Pleurotus.ostreatus.KDQ23127.1
Auriculariopsis.ampla.TRM58449.1
Armillaria.gallica.PBK88411.1
Amanita.thiersii.PFH51582.1
Gloeophyllum.trabeum.XP_00787023
Heliocybe.sulcata.TFK50419.1
Coprinopsis.marcescibilis.TFK229
Exidia.glandulosa.KZV96953.1
Psilocybe.cyanescens.PPQ90175.1
Termitomyces.sp.KNZ76543.1
Psathyrella.aberdarensis.RXW2148
Hebeloma.cylindrosporum.KIM43538
Hypholoma.sublateritium.KJA22546

S10b)

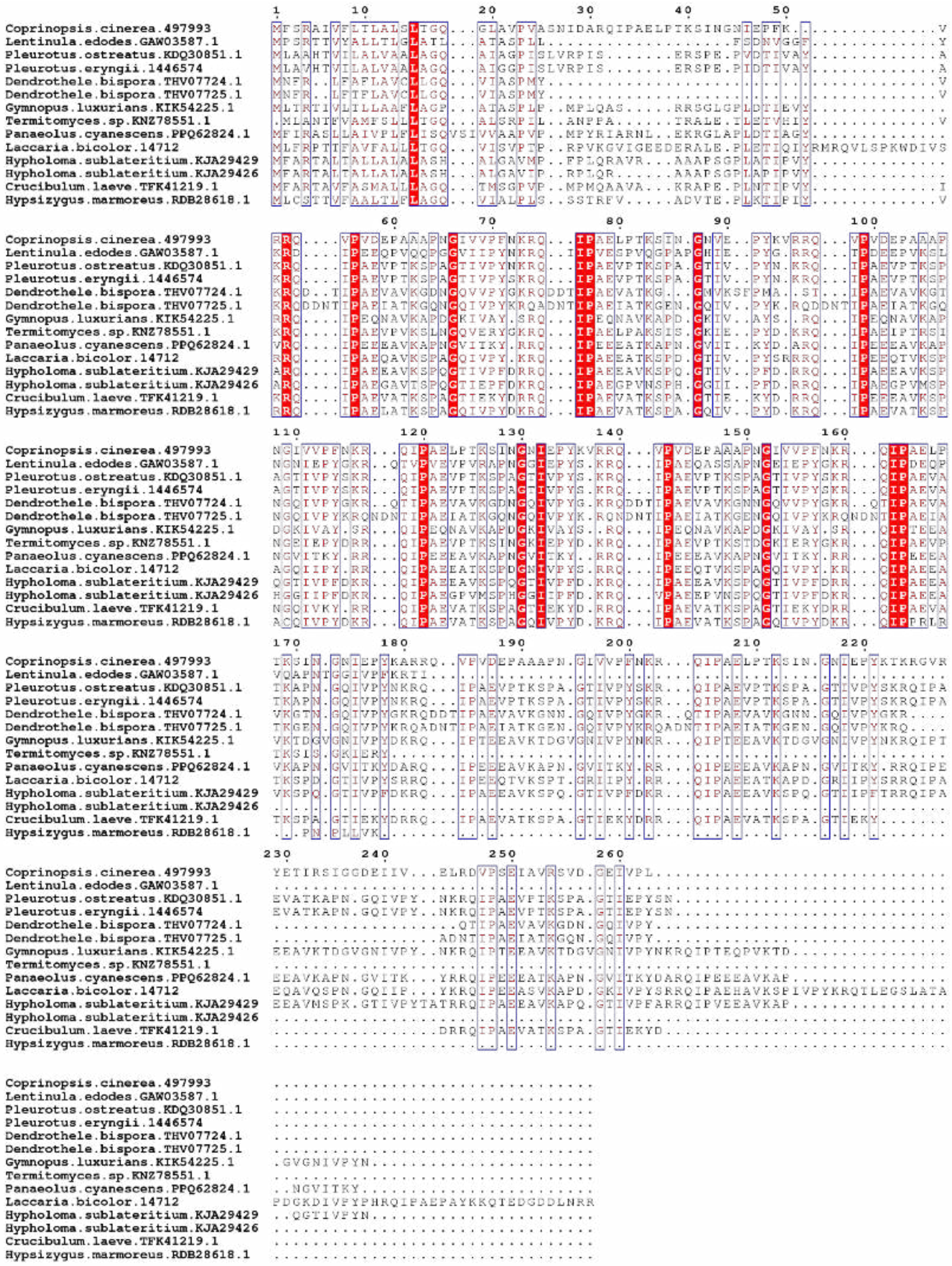
Coprinopsis.cinerea.497993
Lentinula.edodes.GAW03587.1
Pleurotus.ostreatus.KDQ30851.1
Pleurotus.eryngii.1446574
Dendrothele.bispora.THV07724.1
Dendrothele.bispora.THV07725.1
Gymnopus.luxurians.KIK54225.1
Termitomyces.sp.KNZ78551.1
Panaeolus.cyanescens.PPQ62824.1
Laccaria.bicolor.14712
Hypholoma.sublateritium.KJA29429
Hypholoma.sublateritium.KJA29426
Crucibulum.laeve.TFK41219.1
Hypsizygus.marmoreus.RDB28618.1

S10c)

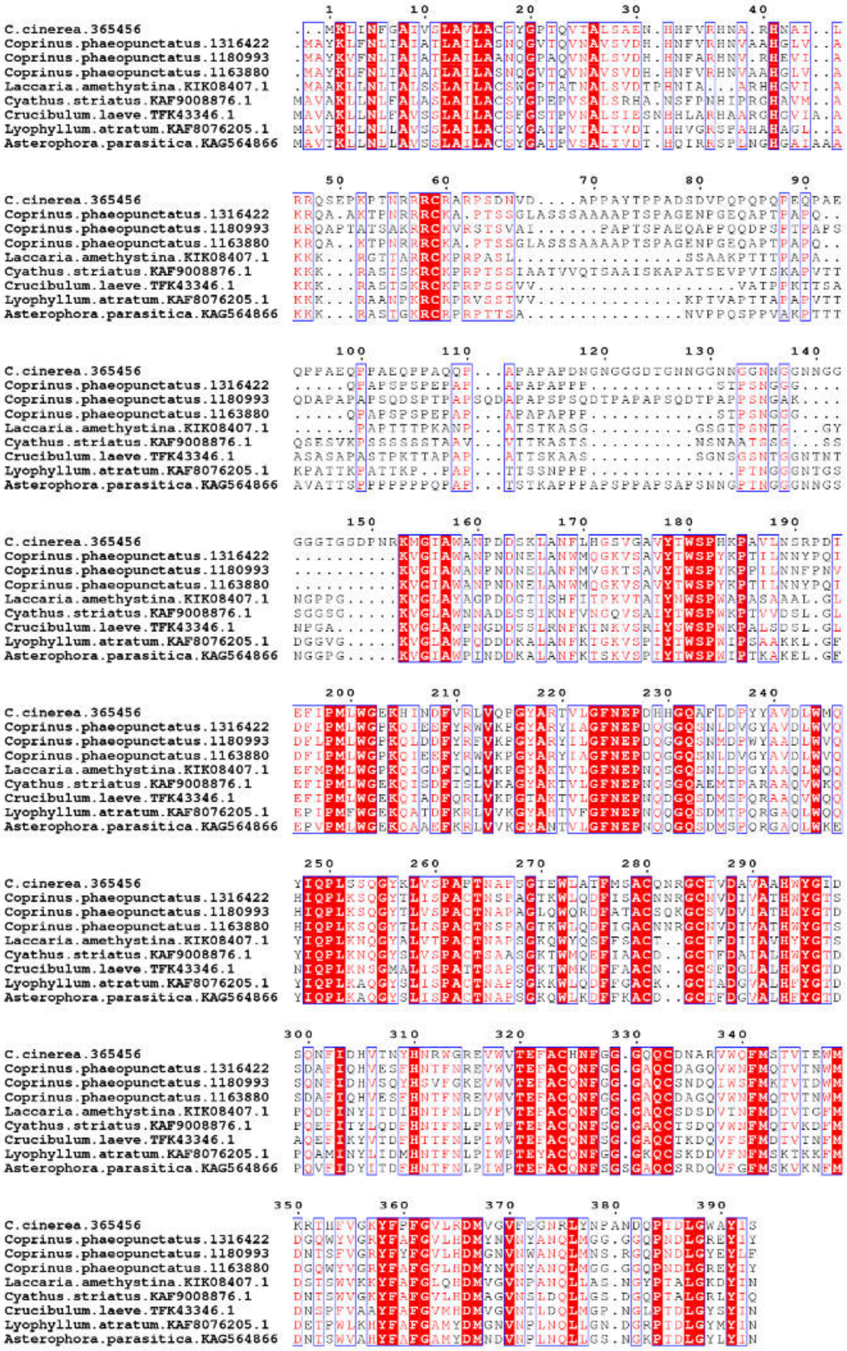
C.cinerea.365456
Coprinus.phaeopunctatus.1316422
Coprinus.phaeopunctatus.1180993
Coprinus.phaeopunctatus.1163880
Laccaria.amethystina.KIK08407.1
Cyathus.striatus.KAF9008876.1
Crucibulum.laeve.TFK43346.1
Lyophyllum.atratum.KAF8076205.1
Asterophora.parasitica.KAG564866

S10d)

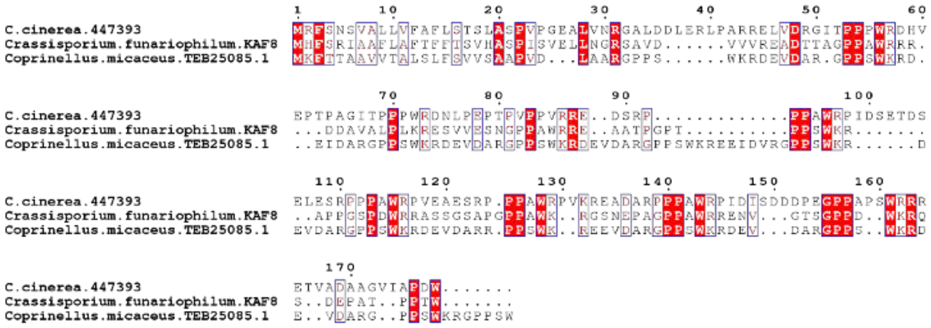
C.cinerea.447393
Crassisporium.funariophilum.KAF8
Coprinellus.micaceus.TEB25085.1

S10e)

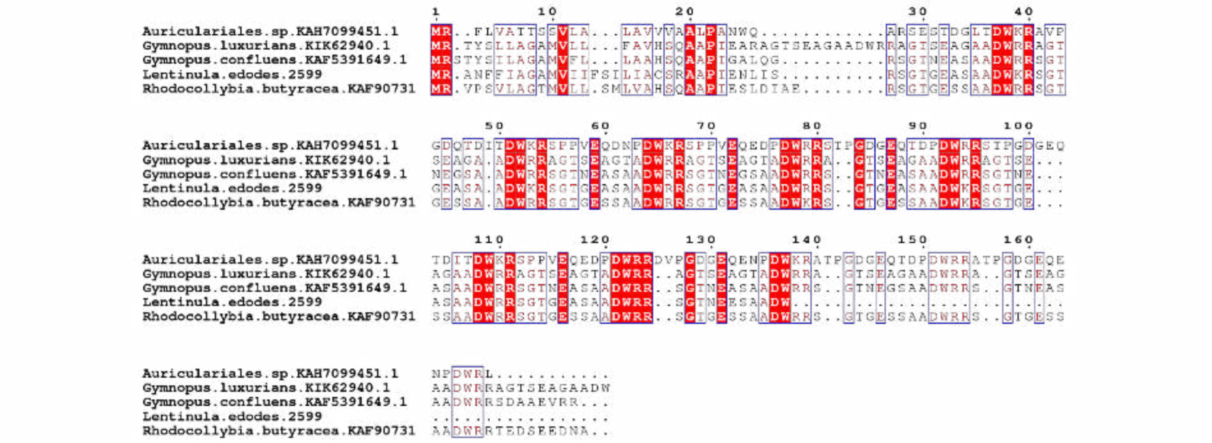

S10f)

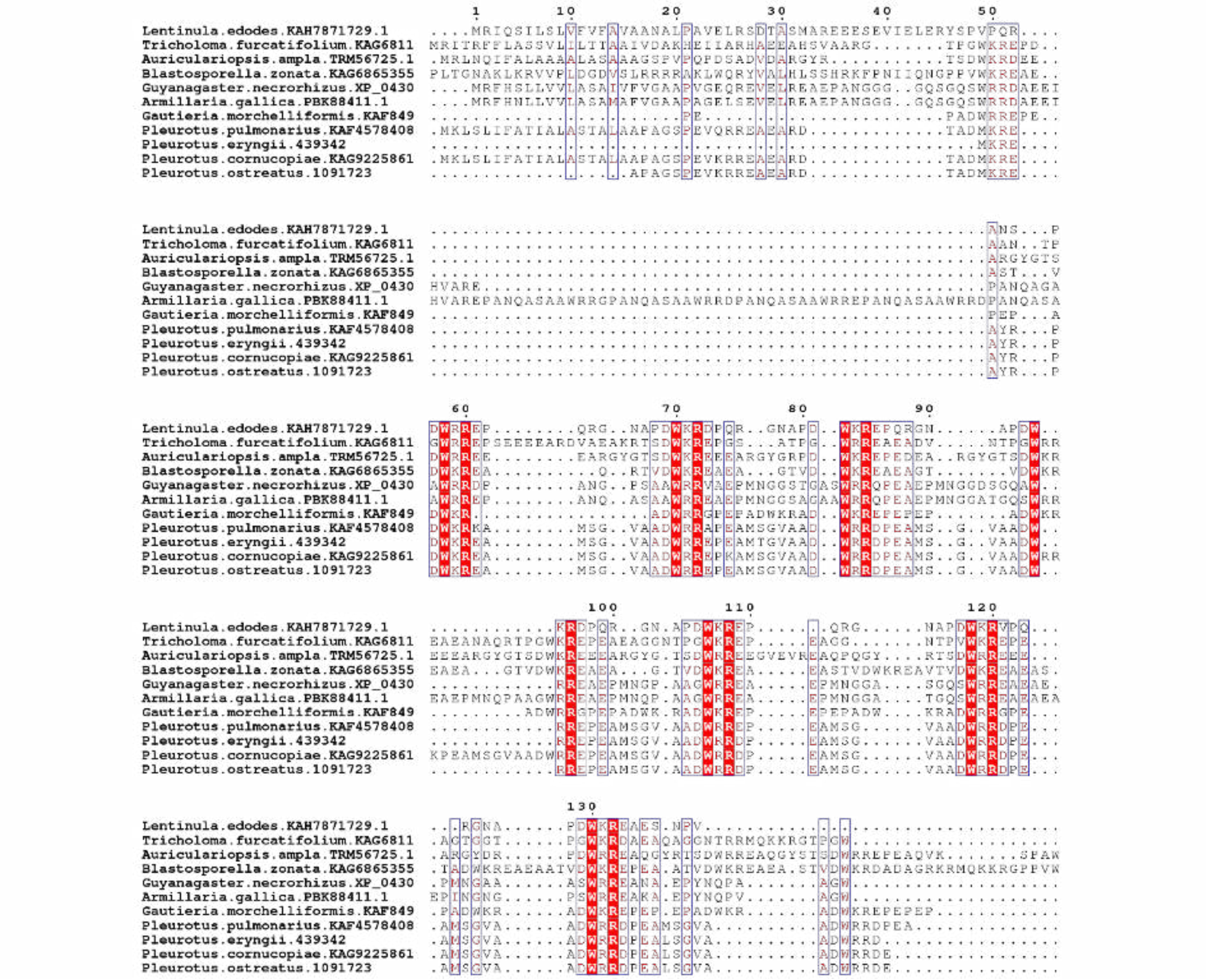

Figure S10: Alignments of analyzed KEX2-cleaved proteins with homologs in other fungal species. BLASTs were carried out using NCBI (https://www.ncbi.nlm.nih.gov) or JGI (https://mycocosm.jgi.doe.gov), alignments were carried out using MUSCLE (MUltiple Sequence Comparison by Log- Expectation) (6) (https://www.ebi.ac.uk/Tools/msa/muscle/), and the graphic rendering of the alignment results using ESPript (Easy Sequencing in PostScript) (https://espript.ibcp.fr) (7). Shown are the alignments of a) *C. cinerea* KEP 490115, b) *C. cinerea* KEP 497993, and c) *C. cinerea* protein 365456 that contains a C-terminal glycoside hydrolase domain (starting at position 153) with two KEX2 cleavage sites (starting at position 46) flanking the sequences

QAAKTPN, QAPTATSA, QAKTPN in *Coprinus phaeopunctatus* and GTTA, ASTS, ASTS, AANP, ASTG in five other fungal species. Subpanel d) shows *C. cinerea* KEP 447393, e) *Lentinula edodes* KEP 2599, and f) the homologs *Pleurotus ostreatus* KEP 1091723 and *Pleurotus eryngii* KEP 439342.

S11a)

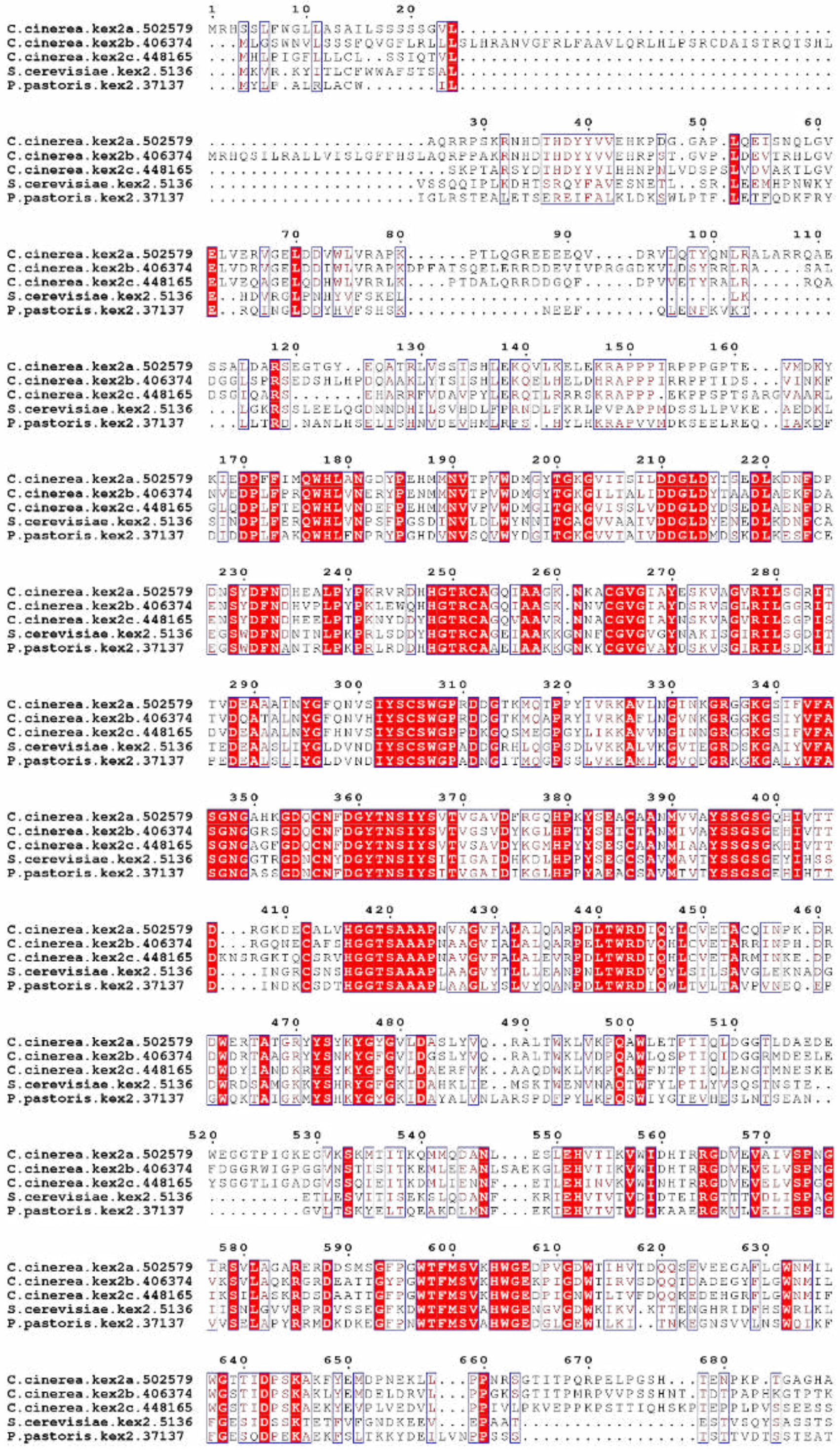

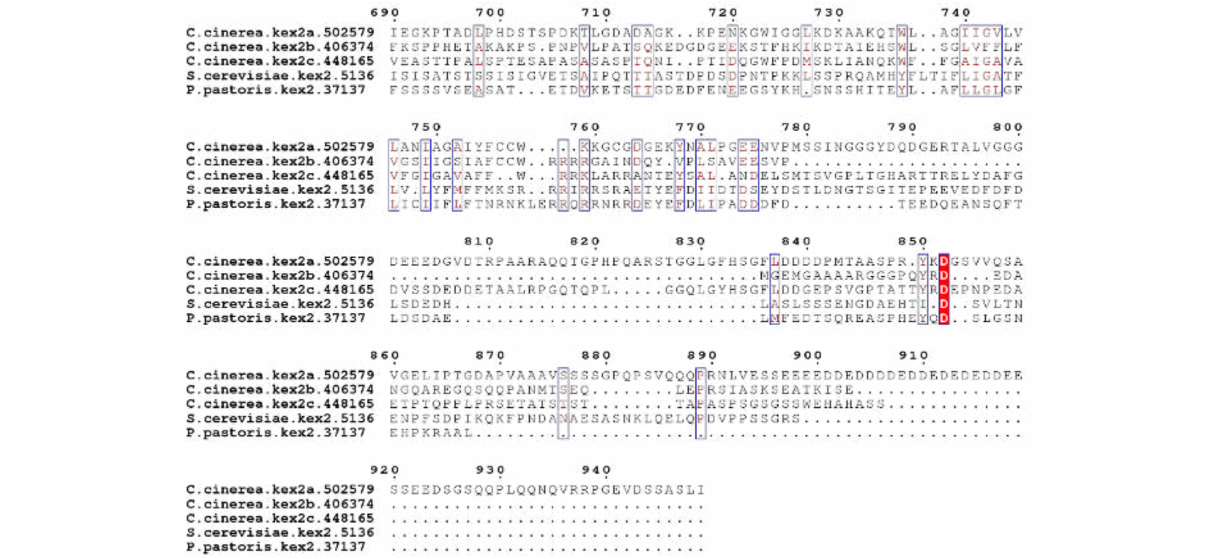

S11b)

	C.cinerea.kex2c.448165	C.cinerea.kex2a.502579	C.cinerea.kex2b.406374	S.cerevisiae.kex2.5136	P.pastoris.kex2.37137
C.cinerea.kex2c.448165	100	54.08	51.59	37.16	39.71
C.cinerea.kex2a.502579	54.08	100	61.33	34.67	38.04
C.cinerea.kex2b.406374	51.59	61.33	100	36.54	38.47
S.cerevisiae.kex2.5136	37.16	34.67	36.54	100	47.28
P.pastoris.kex2.37137	39.71	38.04	38.47	47.28	100

S11c)

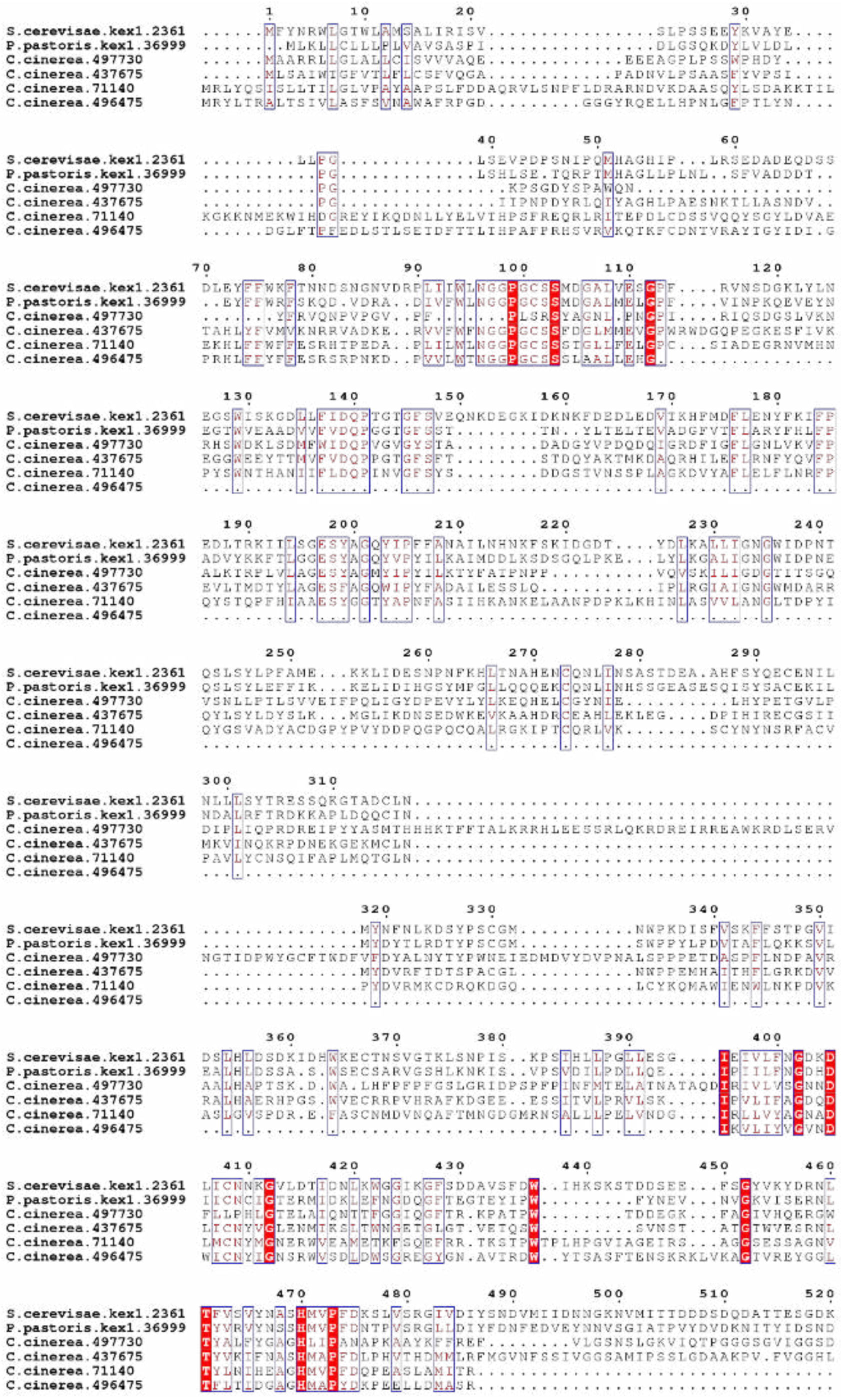

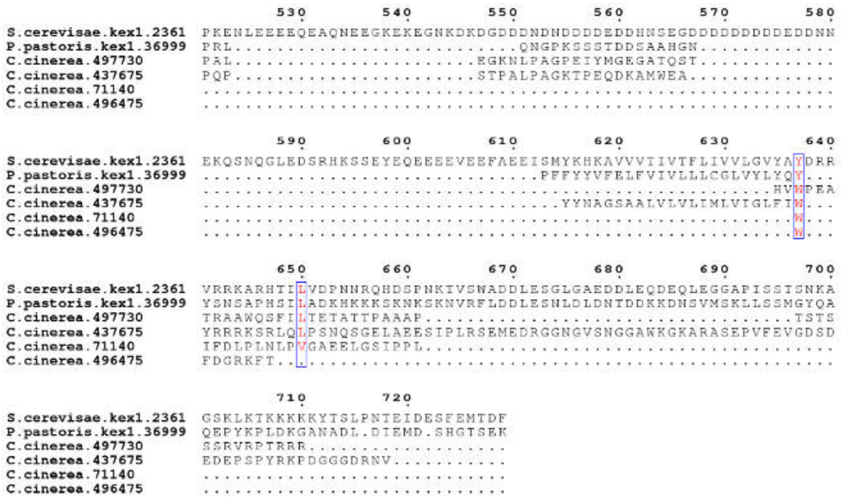

Figure S11: Alignments of *C. cinerea* KEX proteases with homologs in other fungal species.
BLASTs were carried out using NCBI (https://www.ncbi.nlm.nih.gov) or JGI (https://mycocosm.jgi.doe.gov). Shown are a) KEX2 alignments, b) the percent identity matrix calculated by MUSCLE (6) of KEX proteases of *S. cerevisiae*, *P. pastoris* and *C. cinerea*, and c) alignments of KEX1.

S12a)

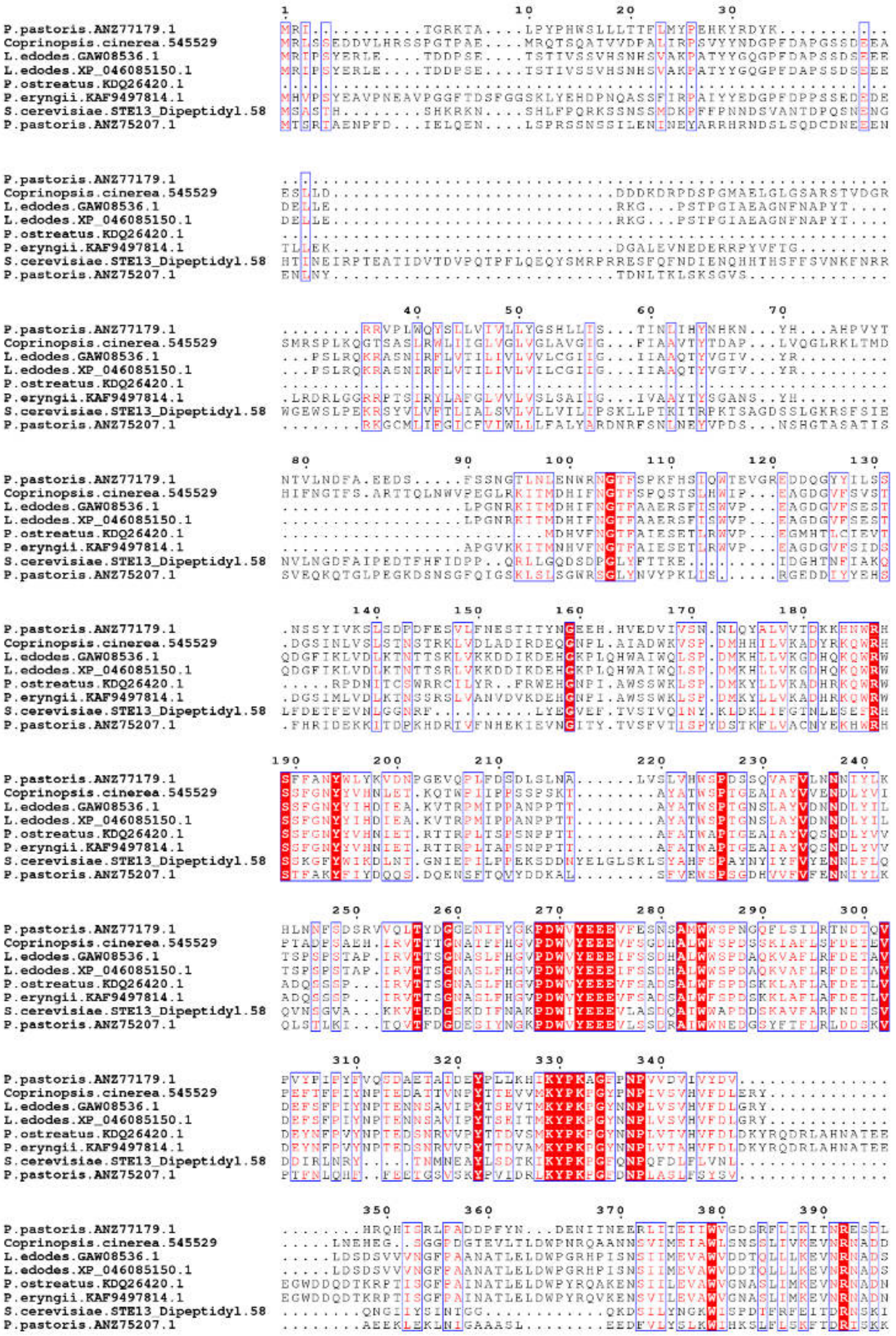
P.pastoris.ANZ77179.1
Coprinopsis.cinerea.545529
L.edodes.GAW08536.1
L.edodes.XP_046085150.1
P.ostreatus.KDQ26420.1
P.eryngii.KAF9497814.1
S.cerevisiae.STE13_Dipeptidyl.58
P.pastoris.ANZ75207.1

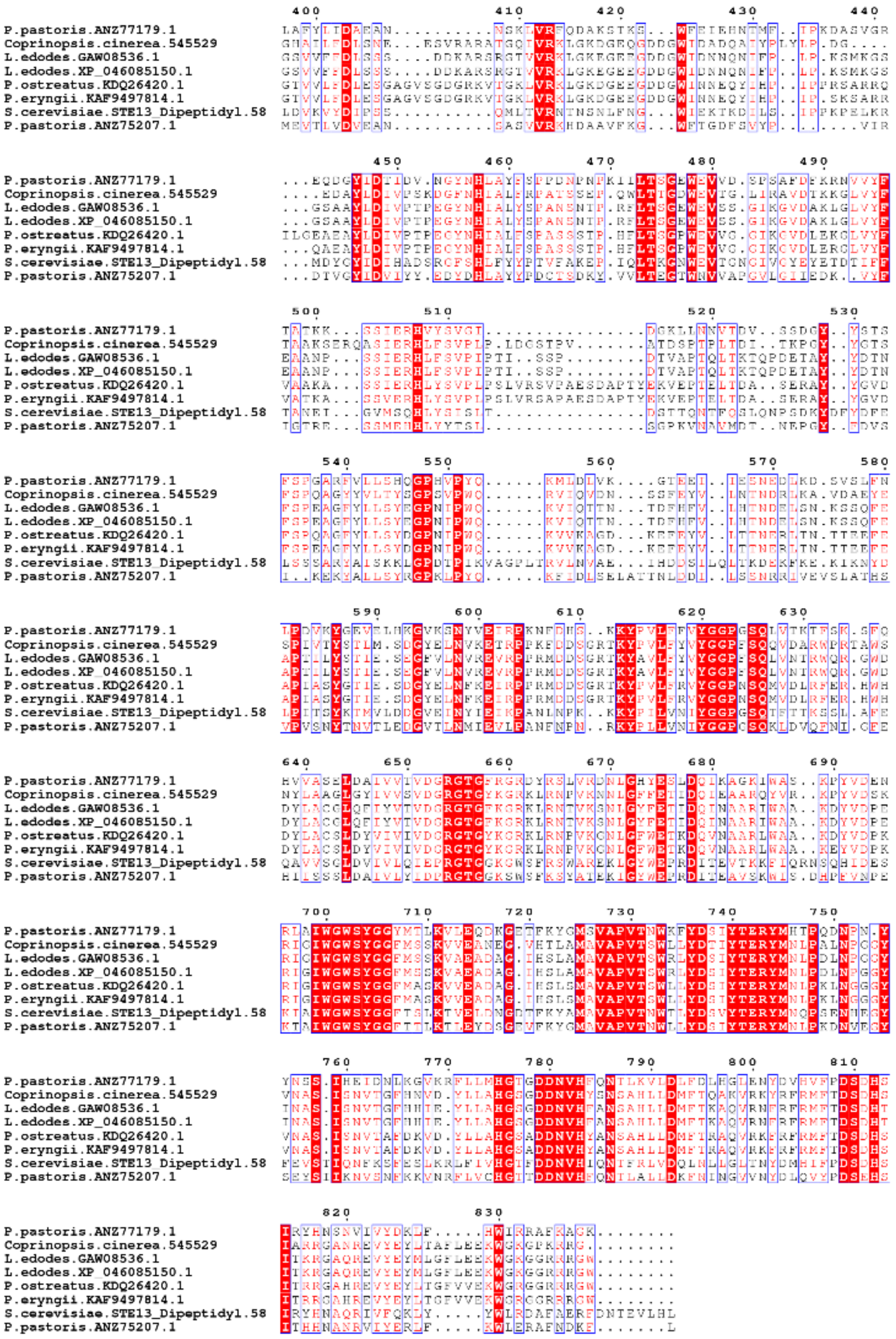
P.pastoris.ANZ77179.1
Coprinopsis.cinerea.545529
L.edodes.GAW08536.1
L.edodes.XP_046085150.1
P.ostreatus.KDQ26420.1
P.eryngii.KAF9497814.1
S.cerevisiae.STE13_Dipeptidyl.58
P.pastoris.ANZ75207.1

S12b)

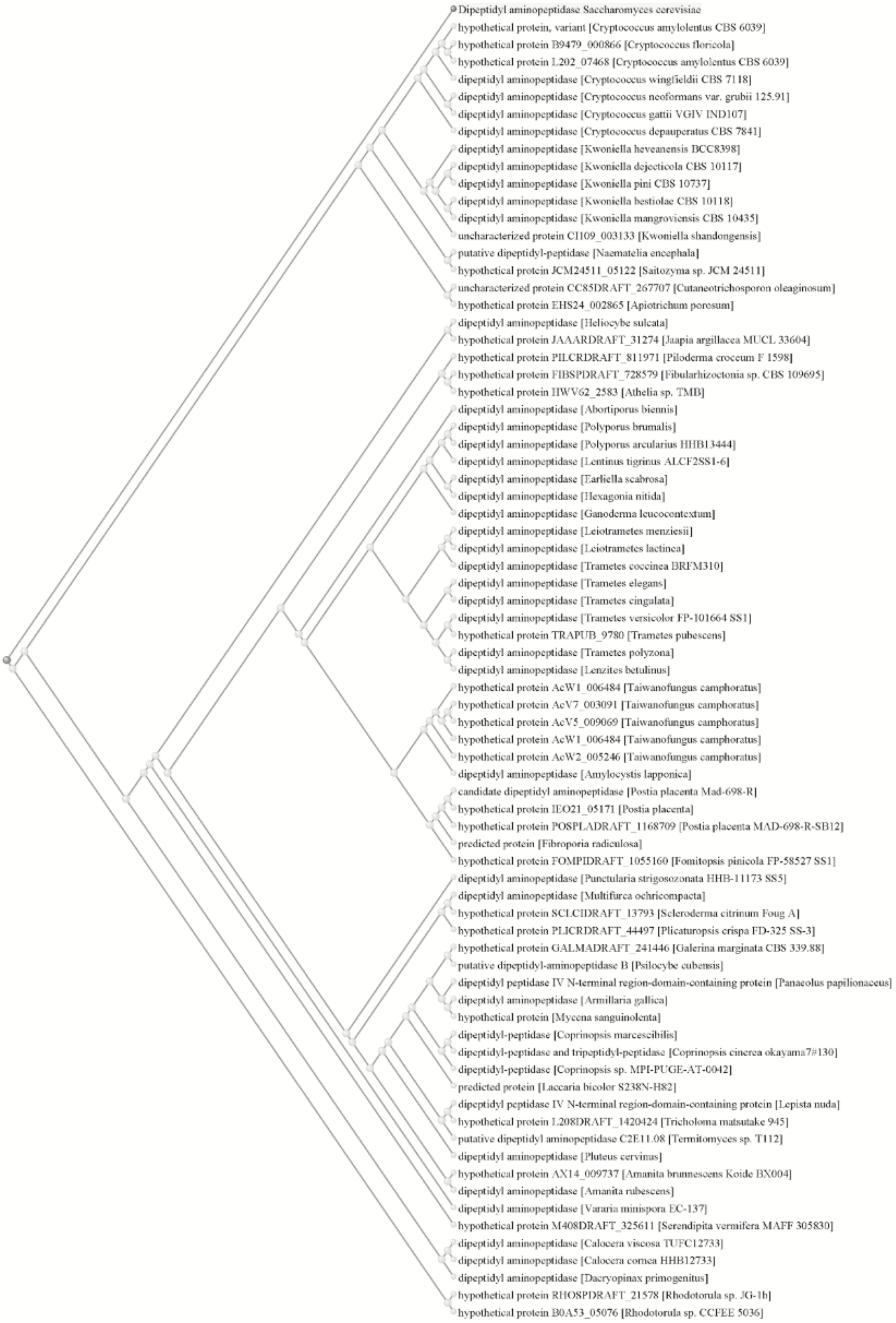

Figure S12: Homologs of dipeptidyl aminopeptidases STE13 in different fungal species.
BLASTs were carried out using NCBI (https://www.ncbi.nlm.nih.gov) or JGI (https://mycocosm.jgi.doe.gov). Shown are a) the alignments of *S. cerevisiae* STE13 with *P. pastoris, C. cinerea, L. edodes, P. ostreatus,* and *P. eryngii,* b) distance tree of BLAST alignments of STE13-homologs in basidiomycetes, visualized using NCBI.

S13

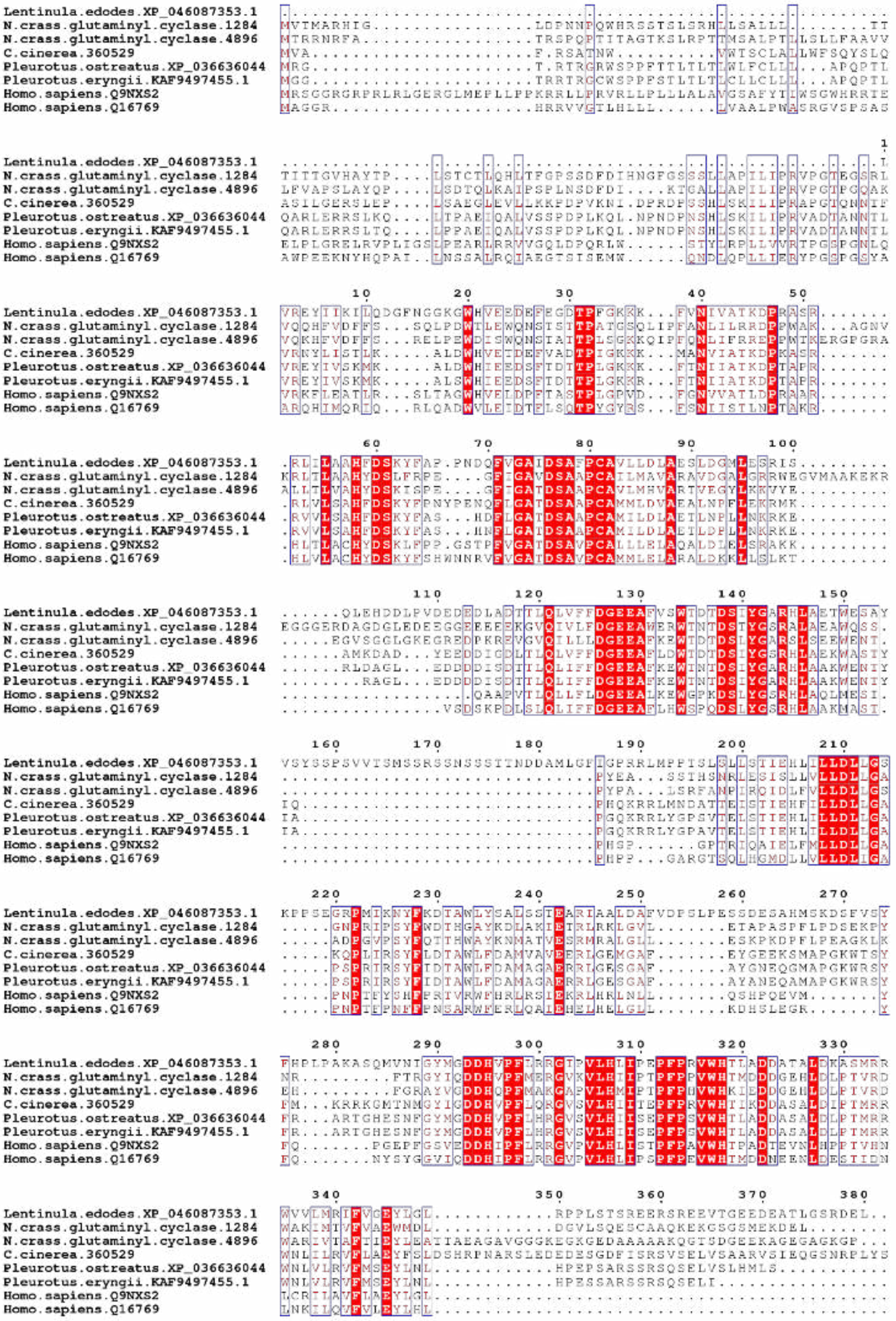
Lentinula.edodes.XP_046087353.1
N.crass.glutaminyl.cyclase.1284
N.crass.glutaminyl.cyclase.4896
C.cinerea.360529
Pleurotus.ostreatus.XP_036636044
Pleurotus.eryngii.KAF9497455.1
Homo.sapiens.Q9NXS2
Homo.sapiens.Q16769

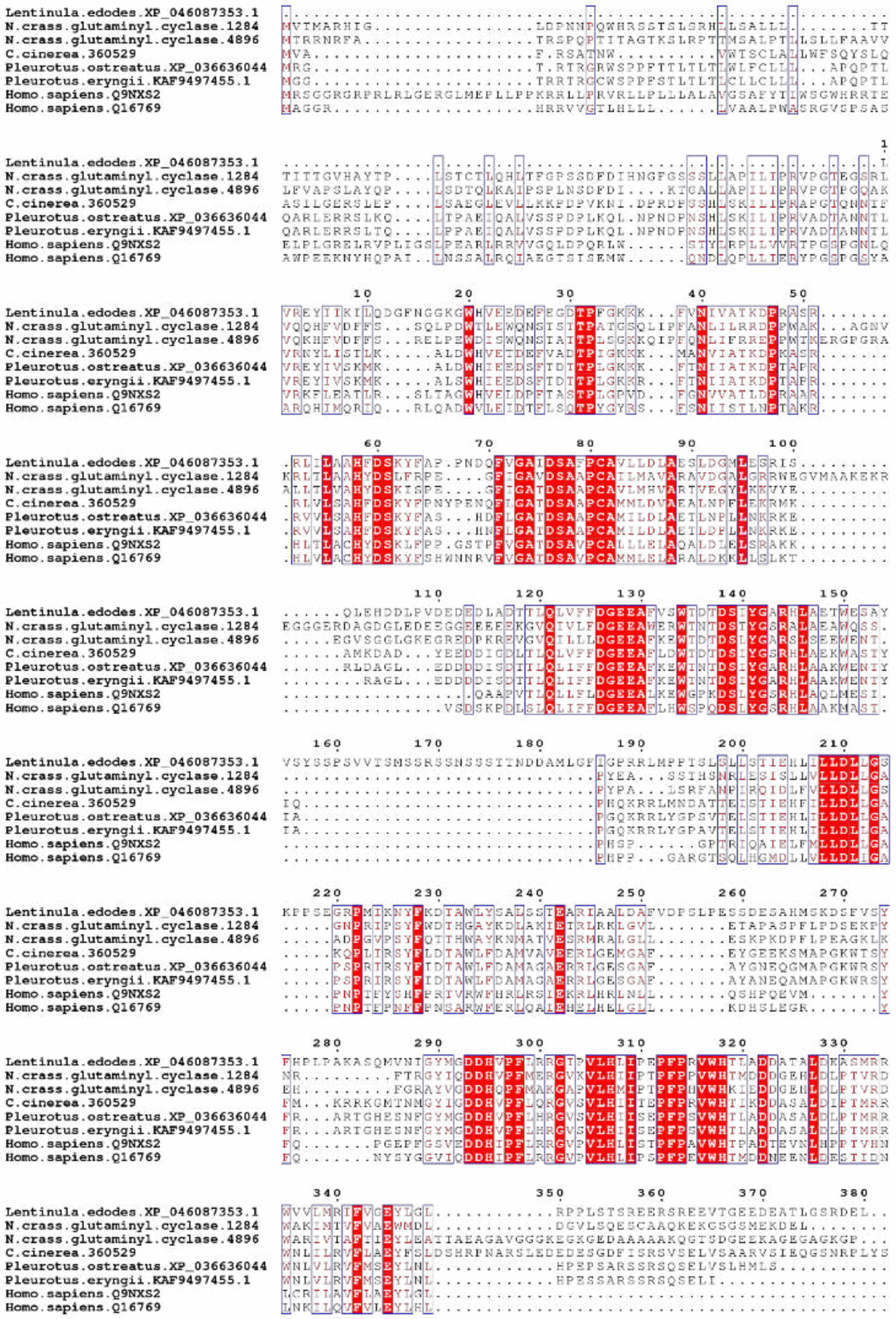

Figure S13: Alignments of glutaminyl cyclase (QC) homologs across different species.

BLASTs were carried out using NCBI (https://www.ncbi.nlm.nih.gov) or JGI (https://mycocosm.jgi.doe.gov). Shown is the alignment of glutaminyl cyclases of *C. cinerea* and homologs from *N. crassa, Homo sapiens, L. edodes, P. ostreatus,* and *P. eryngii.*

a)

Peptide sequence	Endogenous producer	Abbreviation
pyro-QVPVDEPA	*C. cinerea*	Peptide mix A
Pyro-QIPAELPTKSINGNIEPY		
Pyro-QIPAELPTKSINGNIEPYK		
Pyro-QSEPKPTN	*C. cinerea*	Peptide mix B
GPPAPSW		
DDDPEGPPAPSW		
SGTGEASAADW	*L.edodes, P. ostreatus, P. eryngii*	Peptide mix C
MSGVAADW		
SGVAADW		
LSGVAADW		
DAREPIFGF		Neg. ctrl

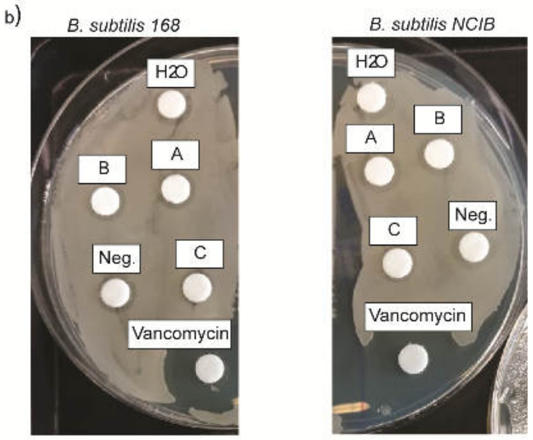

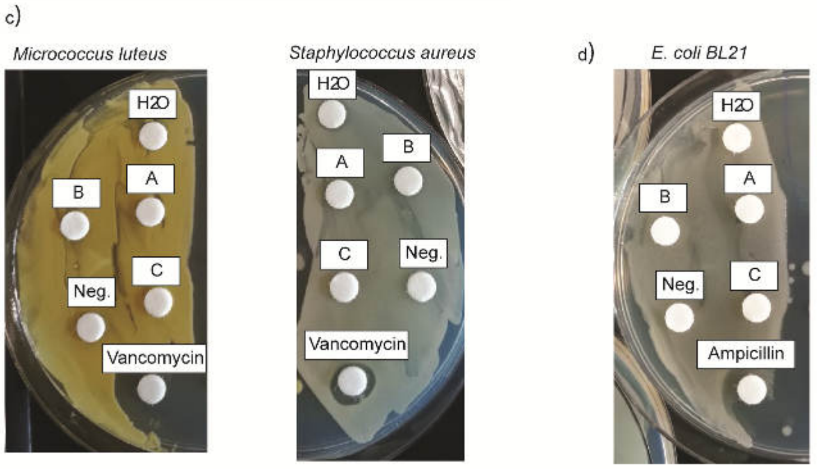

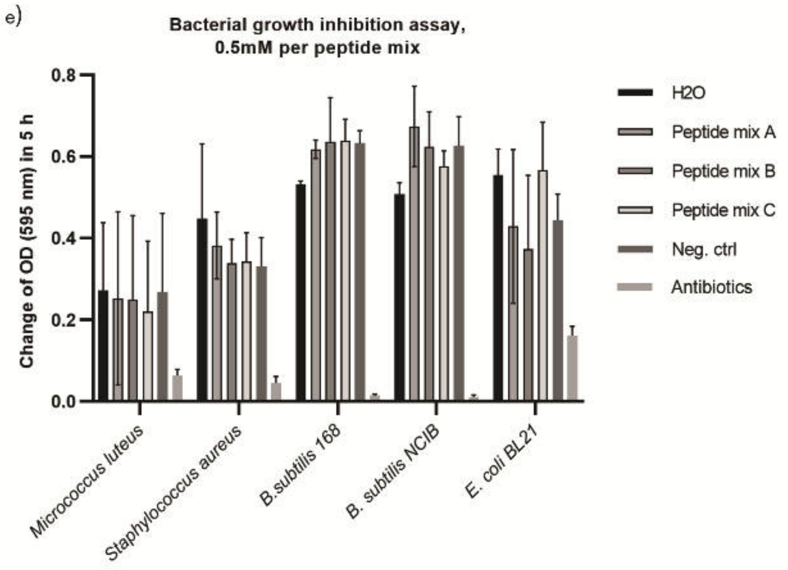

Figure S14: Bacterial growth inhibition assays of synthetic KEP-derived peptides.
Chemically synthesized peptides of each confirmed KEP-derived peptide from *C. cinerea, L. edodes, P. ostreatus,* and *P. eryngii* were tested for bacterial growth inhibition in disk diffusion assays and in optical density (OD) assays in liquid culture. a) List of each KEP-derived peptide confirmed in this study. The peptides were combined in three different peptide mixtures A, B, and C for the tests. b) Disk diffusion assays on *B. subtilis* 168 and *B. subtilis* NCBI 3610, b) *Micrococcus luteus* and *Staphylococcus aureus*, and c) *E. coli* BL21. 70 nmol of each individual peptide (corresponding to approx. 50 µg) and 5 µg of either vancomycin or ampicillin were added to the disks. e) Bacterial growth inhibition assay

using optical density measurement. The same bacterial strains were cultivated in 96 well plates and the OD change after 24 hours was plotted. Each peptide mix had a concentration of 0.5 mM. 50 µg/ml of ampicillin (for *E. coli* BL21) or vancomycin (for all other strains) were used as control. The bars represent the median value of three biological replicates, the error bars represent the 95% confidence interval.

Supplemental Tables

ID	Complete sequence	Detected in			
		This study	Method	Le Marquer et al., 2019	Umemura, 2020
139620	MVKAIVVAAALAAVVPVLSIPINVEYDLEHRDVLDLFERQYNGDEGSTVKAGEDKGGSAVKGGIVPSGKQRTGRRSFDDYDLFERQDPNSGSGEPDEGTKSGGAIKTNITPKGGRKGRRSLDYYDLFERQDPNSGSGEPDEGKKSGGAIKTNITPKGGRKGRRSFDDYDLFERQDPNSGSGEPDEGTKSGGAIKTNITPKGGRK	Yes	1	No	Yes
197636	MRLSLSNFLSFFLLSSLAAAHAHAHDSIPQRRSRSLKLQRRQISLDIPVLDGVLGGGDGPLFPGFPGSGNDNDDDDDDQPDPAPTQEEVTLLPPPMSTSWDPVPVPTPDESSSDSVPEPSPTPSPDPSIPESSSEPVPEPTESVPEPSDPSSEPVPEPSDPSSEPVPEPSTEPSAPSSEEPEPAPTSAPSSSSTPSGLLPTLTSIVGDVTSGLGSIVTEVLPIPTPSSSVPVPEPSVPEPSQPEPSIPEPSLPLPSDPVPEPSDPVPEPSDPVPEPSEPPVPEPSDPLPSLPLPSVSLPLPSISISLPLPSTTEDPGTDPSTSEEPGPGPTSEEPGPSVSVSDPPISEPTPGPSGPSVSDPGPSISDPGPSISDPPISEPTPGPSGSVSVSISEPPISFPSGSVTPSLEPEVPEPSETLSAPIDTSVGIDPGLSFVLTESQLAINTLPSSTITPTISDPDQATATPEPIVTQSATQSFSAAPLPTGIPNKIYPNKRIGEDEDLTGFTLISILFNRELNWPHVIRNENASSQIFAYTPMVIANTLEIGTSSIKTYALQVYIPTSYTGPADQTELGTMYLAYIPTELVDTLAAAIKTRNSKFYTATPGIAGDLAKYVNSGFALRSVQDPGVGNNSGTNGNGAPGNTGATSSDGKSRQDAIIGVVSALGAIALFVLIFLVYRSLKRRRELAHRRLSDPPNADAHGYRPEGREFDQDSIGGQRRRSFYFAEDSLRGYAGAAAQQEYTYNHHQQSQQQMSQRRNINVNPNAISAPILRDNTMNW	Yes	1	No	No
360529	MVAFRSATNWVWTSCLALLWFSQYSLQASILGERSLEPLSAEGLEVLLKKPDPVKNIDPRDPSSHLSKILIPRAPGTQNNTFVRNYLISTLKALDWHVETDEFVADTPIGKKKMANVIATKDPKASRRLVLSAHFDSKYFPNYPENQFLGATDSAAPCAMMLDVAEALNPFLEKRMKAMKDADYEEDDIGDLTLQLVFFDGEEAFLDWTDTDSIYGSRHLAEKWASTYIQPHQKRRLMNDATTEISTIEHFILLDLLGAKQPLIRSYFLDTAWLFDAMVAVEERLGEMGAFEYGEEKSMAPGKWTSYFMKRRKGMTNMGYIGDDHVPFLQRGVSVLHIITEPFPRVWHTIKDDASALDIPTMRRWNLILRVFLAEYFSLDSHRPNARSLEDEDESGDFISRSVSELVSAARVSIEQGSNRPLYS	Yes	1	No	No
377821	MRFSNSIALLVFAFLSTSLASPVPGEALVNRGALDDLERLPARRELADRGITPPPWRDHVEPTPVGITPPPWRDNLPAPTPVPPVRREDSRPPPAWRPIEADSRPPPAWRPVKRAVDSRPPPAWRPISDDDPEGPPAPSRRETADAAGVVGPDW	Yes	1	No	No
392677	MIYPASTLILATLVFNAASVLGAPVRIPSASPPVARALADDPVLPRKVEIVDVPARMRSLDDDPMDARSAPEIDIRDTASSESIEARDPLPEVEEIVRRYPRKVYHDFYQKRSQPSAPPEKRAPEPDTPQIERRFPRRSLYERYQKRSEPASPPVEARAQAAEPPELQQRRYPRKVYADYYEKRSTPPSPPEQPVAREPAPEPETVQARRFPREVYMKHYERRQADPVKHYGRQAETPESAPADATTPDAATPPTDPAAAPAQPSGAAVSGGAAVVQPPQASTPPVTPLNSTSTPLDVNALFHNITSNDAGFQPGTTIQETTVLVHKITHISEPQQKVDTPPVVPPSPPTGSDPTAPSPSDPASSASPSASGGVAADPSAPPVAGGEQAPPQPSPSDAATSPPPTDSSTTPPGAPTTEDGASEPKPEETNSGSESTEGGAAAPPPGEDGAASRRSIGSDASPEPILRRRNLGYSGPAWASYAKRSVTN	Yes	1	No	No
405832	MRISVVSALTLLAATSLTFAAPTTFGDLVNRSERQTGVVKGPERRNIIGVDGDLVQRSERQTGVVKPRNEPPADRETGTVKWKRQNSDRQTGTVKWKRQDDDESDRQTGTVKW	Yes	1	No	No
426342	MQLRFSFFALALLALNAVATPISYADSEDLEIRGKKPNPLPAVNQKVAKLPDDLRRKVFGHWKKQTLQRANARVGHKAIMQELKGQAPPGRWTDRTTAAWAVHQKNKNLRRELLDSDELEVRGRKPNPLPPVNQKVAKLPEELRRKVFGHWKKQTLQRANARAGHKAIVQELKGQAPPGRWTDRTTAAWAVHEKNKNLRRELELLDEVEELL	Yes	1	No	Yes
434504	MVQISTSILVAALAIAPVLAVPIAQEANEFVARDVDLVAEPLVAREPIFGFIKRIFTGKRDFSESEELALRELAEDLDAREPIFGFIKRIFTGKRDLSESEELALRELVEDIDAREPIFGFIKRIFTGKRDLSETEELALREYVDSLDAREPIFGFIKRIFTGKRDLSDLDDLSLRDFEDLEAREPIFGFIKRIFTGKRDLSESEELALREYVDSLDAREPIFGFIKRIFTGKRDFSDVDDLSLRDFEELDAREPIFGFIKRIFTGKRDLSETEELALRELVDDLDAREPIFGFIKRIFTGKRDLSESEELVLREFMDSLDARDPSFAGIAKIGGKALNWLGTAGTLASIPAMFRSSKKDKRDFEDDLVFRDWVLEELDARDFDDDLAYRMFDDEFDARELNELD	Yes	1	No	Yes
440153	MVNCYTLAFAASSLLSSVGPSLVVQAAPVWGSSDFDTLLGRATVANGAANVIPSKRSYDLDQRAPGHKCPGRGGRWKSTLDPGRENLERGIADRTRRALGSASDLDLLERFIANLEERNLELEEGIKERNNLELEGRAGRCTVCGRWRGQVRGTVHVCPGSTNDPKEPGVKRCSVCNQWLGTSGGVHTCPGGGGRGKKGN	Yes	1	No	No
446025	MRFTAATILTAVFLASSGALAFVSIRHLEALHASDPPWSSIINWHQFDDESYEVRDINDVDLFERDFDYELETREPRRRRGGGRRHSKGRFNAIAGALGDAAGQIGGAAVQGAMAQRDFLDEFDLVERDFDDFELEAREPRRRRGGRRRGSSGRFDSAVGALGNAVGQIGGAAVEGAMAQRDFFDELDLVERDFDDFDLEARFRGGRGGRGGRRRGGRRGGFRGGRGRRGGRGGFGGGPGAAAAQAAGGAGGGEAPAERSFTDYDDLFERDFDDFDFEAREPRRRRGGGRRASKGRFNAVAGALGDAAGQIGGAAVQGAMAQRDFEDELEARRFGKIFRGIRNRFRGGGSAAVDAAAQAASANQAREFDEFELKAREPRRGGFGRRGGRRRRGRGRGRGRRGGFGGAGAGAAGEAGGAAAGAASAEAAAAPAARSLDEVEVEARELAVSSPIEVSA	Yes	1	No	Yes
452194	MKLLPFVSASVVLAAGLARAQVDYNPYDSRDLTEDALFEARDFDDIGAYEARDFGDFDEYAARDFDDEGVAARDLNDYVDYALRDVLDQEIDHALRELDDDFAAREYEPVEFEAREVDSFDYKAREFGESDDLSLRSTVLERFSTRELVDELNARLKEIEEKREAEVPSGRRRKARARAGSRHDLPSGLTPSQRRRLIKQRRRAQREKERTRRLLEQKMEEIKTNAKKRRRRHSKGKPDPAERPAAAKPAEKKAGEAAEKDTKKAEISKPKRDEPQVKKDTGSPADRPTAKASPASSEKKAADNNAKPAAAGDKEEPKPAQQ	Yes	1	No	No
454503	MRFTLSSILLSTSVGALALTVPHVRPANHHDLASTDAGSSEPRDIAYDFDSLDARGWEDIELDLRDLETLLELQGRGIGGLAKFAKGPIQKMASSLRRSGGAKSKKASVSPKKPKTSLPSKKAKSKSSSASKAKSKSGSSKSTSAASKKQKSAKKSSSKAVSSGKGSKKSANSKKGKAKSPVASSSNLKAKGSKKASPSGSPKATKKSKSSGSKKSATSVKAKSGGKKSSSTATKASKKAGGSKKAPAASKKDGQKGKGGKKGGKGSGGGKGGGKGGKDGKKKKGAIRNLNFNVDPASMVAAGAGVANLFMN	Yes	1	No	No
456645	MVHFRATLAVAALSVVSSVVAVPVGFDEYDARRELTDLELVERDPLFGAIASVAGNLLGGLMGGGGSSSPPPPPPPKPKPKPKPRRKAPPKKKAAPKKKAAPKRKAAPKKNNNRRRKRDLEDLEDLVTRAIYEFDLDELL	Yes	1	No	No

481379	MVKFATVASASAIIASVAPSVIAAPIVQADDALVEREPLFPLLFGAGSLIANAIKKRKRDLGDVELEQREPEPLFPLLFGAGSLIANAVKRKRGLGDEFELLERGYEDGLEQREPLFPLLFTAGSAIANAVRGRRKRGLEDMELEMREYFDEFEVRDFEDVLDSRYYESLDELD	Yes	1	No	No
490115	MRIASAALFFVSVLSVVSALPTPEVDPVAPVAGVVAREPQMRPPSWRRDPEPQSRPPSWRREPEPEPEPQYKAPSWRREPEPQSRPPAWRREPEPEPQYKAPSWRREPEPEPQYKAPSWRREPEPEPQYKAPSWRRDPEPQYKAPSWRREPEPQMRPPSW	Yes	1	Yes	Yes
497900	MVSYPAVLVLLLTFALWGAAPRVFVHAELLGHVDQLADKTFDFIIAGGGAAGSVLASRLSENPKFSVLLVEASPDNVGVLEIMAPGLAFQIPRTYNWNFVSIPQRGLDNRVINIPRGHVLGGSTCINGMLYTRGSSDNYDNWARNVGDQQWSWKALWPYFKKHERWVAPAGNRSIEGQYDPKAHGYNGKTFVSLPTNGPDEHSFRCLNNTKLQPKLFPLTLDINGGKPIGLTWTQWSIGNGARSSAASSYLTPDVRRRPNLTILVNAYVTRVVPSSVNLNGPLDIRTVEIAPRAGGASKTLTAKKEVILSAGSFGTPRILLSSGIGNKTELDLIGVKVIHDLPDVGEGLTDHAAIEARWGTTATPLPPVDPAEALAEWQANRTGPLTMSSVSSPQLLWNRISSDSPVFKKYRDPAPGPNSPHIEMTLRSPGGPVTPESIVLLTPYSSQHPPSRRKLPMLTPPSNGMHIAGGTVKINSTNPFDDPLIDYNFLGHPFDIEAFKEGIRLGKKFYSGPVWQGYRTSFLGPDPETLSEDEFLNAIKPMVSSWQHPVGTAAMSKRGSKQGVVNPDLRVKGVRGLRIVDASVIVSL	Yes	1	No	No
**<u>497993</u>**	MFSRTIVFLTLALSLTGQGLAVPVASNIDARQIPAELPTKSINGNIEPFKVRRQVPVDEPAAAPNGIVVPFNKRQIPAELPTKSINGNVEPYKVRRQVPVDEPAAAPNGIVVPFNKRQIPAELPTKSINGNIEPYKVRRQVPVDEPAAAPNGIVVPFNKRQIPAELPTKSINGNIEPYKVRRQVPVDEPAAAPNGIVVPFNKRQIPAELPTKSINGNIEPYKTKRGVRYETIRSIGGDEIIVELRDVPSEIAVRSVDGEIVPL	Yes	1	Yes	Yes
503649	MMLRTNFVLLAVAAFSTLGSVFAAPTRTFSDDFDARDLAFEDILDARGGLAIPKPTHGTDGGKASLNTIGKGRRAFLEDILDIRGGLGIPKPTHGTDGGKTSLNTIGKGRRALLEDFLEARGGLALPKPTHGTDGGKASLNTIGKGRREFLEDLLDARDLLDEILDVRGGLALPKPTHGTDGGKASLNTIGKGRRDFFEDILDARGGLALPKPTHGTDGGKASMNTIGKGRRDFWEDIIEA	Yes	1	No	Yes
546172	MRLFNSRSLFLSTITSGILFLLAVQSAAAPVQLQIRESSTKSLQIRPRTNYAARAAAEALLPRIVTGEKDPTPKDAAETPAEMRELEASERLQRTQAWVQAQKGVSYKKVYPGRGTDASHIPTTSHDKFANPNPPQWIKTARRQGRLMPPSRRPWRRVTAAKGPEEQGTNGKNTQPGPAESGKRPEPKKADPLPPVSPPFSGTDGKGSSQTPQTDPPENGKKPEPKRSNSISSASPGRAQNLEPGSNGPGKGGVVQSTQPEPPEPKGSNSASPASGSSGSAPNLEPGSNGPGKGSVQSPQPAPEPPRSNPVSPQAGRPQNQEPGSNGPATGGRQSPQPGPPESAKRPEPKRSDSMSSLSSEKTLVG	Yes	1	No	No
**<u>447393</u>**	MRFSNSVALLVFAFLSTSLASPVPGEALVNRGALDDLERLPARRELVDRGITPPPWRDHVEPTPAGITPPPWRDNLPEPTPVPPVRREDSRPPPAWRPIDSETDSELESRPPPAWRPVEAESRPPPAWRPVKREADARPPPAWRPIDISDDDPEGPPAPSWRRRETVADAAGVIAPDW	Yes	x	No	No
545670	MVKASSIVVASLFAVGSALAFVSSYDDLDSRDLIEADLYERDLYEPFELDLREYIEAREVEEAIQFYQRDPFIGRILGGAKRFLFGRGLDIEDLSDRDVDDLIENVVARDPFIGKILGGAKRFLFGRDLEEIEAMEARSPFIGKIFRGVKNVLFGRDDLEAIEARDPFIGKILGGAKRFLFGRHIELGGSQELDARDPFIGKVFGGVKRFLFGRDGVDFETRSMDGSLDELD	Yes	3	No	No
489010	MVQISTSTLAVAALAAGAALPALAAPIGDSSFVEENAARSMDIEGLEARGLLLPGGPRRPIGWKGVMPPRSLDGSDNLETRGLLIPGGPRRPIGWKGVMPPRSLGMDDLEARGLIMPGQIVPPGWAGAPSVGLLSSGTRKRPRSLDADDLEVRMLPLPGQSLFRRPRGANPLLVYKGVRPRSFENEDLEARLMPNPRLPNLGMPSNRFIYKGVGVRPRSFDIDELEARSIDELD	Yes	3	No	Yes
21614*	MNKLLPFVLAAIALNSVYVDATPIRAADLAARAPNPQLGGPKIPIPTGVPTPVAISRRGVTNDELAARQLGGPRIPIPSGVPTPIVESRRRAVDDKLAARQLGGPKIPIPTGVPTPIAISRRGAANDEIKARQLGGPKIPIPTGVPTPIAISRRGVADNNLAARAPNPQLGGPGIPIPTGVPTPIAVSLVTDIQEPGSTPSVEFELPGSVLTVAVPVPTASEGVSVLVISGTAVSQPVISGAPVSVPVISGTPISVPVISGTPISVPVISGTPISIPVISGTPISVPVISGTPISVPVISGAPISVPVISGTPISVPVISGTPISVPVISGTPISEPVISSTPITVRPTDSADISSSAPVVSIPVPVSSGVLSSVEPGPTESTSIIVSGSISESVRPTESVSVSASASVTARPTDQASLETTTVVQTIPRETGLVTVTRTLTRIVGPTGVAGLPGRGQGRPGRGQGRGQSRPGRGQSRPGRGQGRGQGRPGRGQGRGNGVGVGRPGGRPGQGRPHIVTVRPRPPVVTVRPTGSGVVTVRPTASGSGVVTVRPTANGSGVVTVRPTGSAPVSIPVSSSESPQTSGSAIPSGSAIPSGEETSVFVTSRPTAFSSVFESESGSVRPTGSGVESSGVGVPTESGSVGVPTESGRVTVPTESGSVIVFPSVTARPTSAFA	No		Yes	No
489256	MRLSTSFVTLAAVLAYKSASVLALPMDASADLSLVERDIDASLELEAREFDFEDLDAREWTDELEVRDFDDLDAREFDEFDIEARGFDEFDIEAREFDEVQLEARKSGKGASKTGSKSVTPAQAANSPALKQKTNNQSSTTKKTTTAANKQSSTTKRNTGSNSKQSAANKQTSTTKRNTGNNKQSLSTKKSTSSNNKQSSTTKRNPNSNKKSTSTKRTTSSNNKPANNKQVSSKKNKSPSGSPNTKTTSNKRNSSSTTKKDAKKKTTPSTNKANNNNKKVTSNTGKSPLSTNRKNNNGNQRTQGEGSRRNRPNNFSGGRLGGGFAPRPVGGFSGPRYGGYRAGGFGGGYGGGYGGPRYGGYHAGGWY	No		Yes	No
10933*	MKEIGGSLPGHKTWVAAPPFFPSLILLDLTLLPALVDVEASKKPLETKYKIRKHTMPPKAGLSTTRSTRRAPKTPEQKAREEEARKLRAEERDKAKQQKAAQQEGSKGGKGKGKGNKRKITSRAFIESDEEGDEADEPRPKKAKTTAVDGGSATSQVGDDEDRAADRGSAASQVGDDEDEAAARGSSRGPAGDDEDGAADGGSATSQAGDKEGGAADGGSAASQVGDDEDRVSGFHFV	No		No	Yes
488795	MQLRFSFFALAILAIGVAAGPLPQGSDLVIRGKGGSKGPKPNLAQRVSNLPPELRNQIGNDLRTHKTIQAHFGPGSKHDRLMKEMNGKVPPNKWTDQATPAWSVFEKNRKLRRALESFDDYLEVRAKHKNGKKGPNPLKPPKASAPPKKPAPKPGPKPNLAQRVSNLPPELRNKIGNDLRTHKTIQAHFAPGSVHNRIMQEMKGKVPPTRWSDHTTAAWRILEKNRKLR	No		No	Yes
492752	MVHVSTQALLTALAASPAIAAPLARQFESAEEQFSRDYVEPEFDARDFTEALDVRDPFIINKGLWEFGKKALAAMAMGGGMGVAMAPAFFAKEKGKRSFIDEGELDARDFFDGDELEAREPINLQGLKAAASAVKTFVKANNRLALPIIGGTAFGLGAWGRKKTLRSFEEDLDARDVFDSDELHSRADDMEYLVVRADDGSYHLVARDPNIANILKAGPTLVKGVLSSNKIVTPLVKAAKAIKNNRRLAYPMLGATAFGLGAWGRKKTLRSFDDDLDVRGLFDSGKLDDLD	No		No	Yes
464658	MKCTSHACVFTAFLAAASSVLASDITHDDLSARAADVFEGTLSARSNALFDDVESRSPDPLFFLPALIGTVARVGAQVGARVGARVGGRAGAKAAAKKGAKKGAKKGAKEGGKQGGEAAHKRHQQKKNEKKKRDFEDGWELEMRALDGFAEEFAEVVTREFMESWSDLVERGVSEGAVEDVFVRALAEWDELD	No		No	Yes

447242	MVQAATLVLAAFAVAPVLSAPLSQPTAEEESFTRSTDIDSDALELRDPKFNFGNFIKKAVGVAKTVGKVAIPAARLFLRDTD GNLYEIRALDDVTLDPDLPARDILESDDALLEREFADLDTRDPRFNLGRFLKKAKGVVGKVGGVAGKVAGVAASLALRDEL DGLDLDARAYQELEELLEREFADIDELEVRDPRLRLGKLVKKVGRVAGKVGGVAGRVAGIAGGLGLRDLEGMETEIREVSL NDLD	No	No	Yes
375232	MVKISTVAVTSALVASVAPSVMAAPCLRGVTPTRLRTMDLTARELEAIELYERDPLFGLIAKGASLLGGLFKKRKRDLGDAF DLYERELQFEDMDARDLAEIELYERDPLFGLIAKGVGMLGSIFKKRKRDLGDFDLYERDFENMDVRDIFETLDARGFFDDV EVRELADEFGARELFDDYEVRDFDLNELD	No	No	Yes
381674	MVALSKFYILSLIPILASLSAVAGHSPDALSPARLSRRNNLVDDYNHRRELLEDILYERDLLEDMLYERDLLEDMLYERDLLED LLYERDHETKPKPFVKKPFGTKDAIAYIYANHPFYAPSKKKNVVKRRGVRK	No	No	Yes

Table S1: KEX2-processed repeat proteins (KEPs) of *C. cinerea*.

The columns indicate the JGI IDs (https://mycocosm.jgi.doe.gov), the protein sequence, and whether the protein was found in the two other KEP detection studies by Le Marquer et al., 2019 and Umemura 2020. KEX2 cleavage sites are labeled red, signal sequences are labeled grey (assessed using SignalP 4.0, Petersen et al. 2011), potential STE13 recognition motifs are labeled green. Six proteins whose ID is labeled blue were analyzed in-depth with a heterologous expression in *P. pastoris* and the construction of KEP knockout strains in *C. cinerea*. KEP-derived peptides were detected of the two proteins whose ID is bold and underlined. KEP 447393 was not found in our screen but detected manually upon inspection of proteins with a signal sequence. JGI IDs are based on the protein assemblies by Muraguchi et al., 2015, or by Stajich et al., 2010 (labeled with "*"). In the KEPs 447393, 497993, and 434504, some sequence repeats are separated by single arginine residues. These are also indicated in red.

	ID	Complete sequence
Lentinula edodes, Umemura 2020	GAW0 1176.1	MRIQSILSLFFVFAVAANALPAVELRSDTVSMAREEEPEVIKLERYSPEPQRANSPDWRREPQRGNAPDWKREPQRGNAPDWKRDPQRGNA PDWKREPQRGNAPDWKREAE
	GAW0 1831.1	MVYSRSIVAAIIAASALSALAAPVPEGQGGKPSEGKAPSTADQKGQPAGSNDMHKHDGPGRNRNHGHGHRKHSHGNDPMSATPVVDLPT DTTSPTLPIETDSSSPSTGSAAATADASPTHTSSSASSHSTSKDTSSPDSKSTVSASSTSKSEATPTPHAAASRREVPGAEEQRSEGAENSEKKKKK NHGNGPKKGKGDRKKKHSGTGSSEKSHGGEKESSPKKQRRTIPGQEHGQGKHQENHSPIHAGGRKPNHGGERHPRTLPGQGHMQGGHEE EHGPIHAGGRKPNHGDERHRRTLPGQEHRQGGHEEEHGPIHAGGRKPNHGDERHRRTLPGQEQQHGQGEGEVDHAHGGPGDEGEKNA MEDGKKHGDKKKGTPGGRKQRNRGGRKDKQHGGSGGEKSTGRSEDKDKEVPGAEDGKPEREGRKSQPQMGRRSPQASVSYGADLD
	GAW0 3513.1	MRLISISAIYIILQGFILTVCAAPVNNYSDVNTTSTNTLQARNYENRRTIKNSSALEELKGQGLSNTVTPSDPSQDTSSPPATSTSSREERRSIKEKV HNALEALKGNTPSSGSSSNPSPDFSPDTSSSATPPERRSRSRYENGRSIMEKLTHVVEALKGQGSTAVDSSSTSPDPTQSSSPGKRSFSDGKESRS IKEKLSNALAALKGQTPSSGDSSTSTASEPSDTSSPSPTRRSLSRRLMLAVAVTVSEGEALTPFVSPSQIAVRDVIRELLEAAAPSLALGTHWRLD WENIPSPKADAKSRIEFSMTGPEKCGGTCTAWVEEKKHEIKDHSGKVIYP
	GAW0 5669.1	MRFFVAQIVSLACVLATVQGLNINRGEPAGVIRGEPAGVIRGEPAGVIRGEPAGVIRGEPAGVIRGEPAGVIRGEPAGVIRGEFEAREPTRKIHG RELEFEAREPTRKIGGREPEVEVREPTRKIHGREPEFEAREPTRKIHGREPEFEAREPTRKIHGREPEFEVREPTRKIHGREIEAREPTRKIKP
	GAW0 5670.1	MRFFVAQIVSLACVLATVQGLNINRGEPAGVIRGEPAGVIRGEPAGVIRGEPAGVIRGEFEAREPTRKIHGRELEFEAREPTRKIGGREPEVEVRE PTRKIHGREPEFEAREPTRKIHGREPEFEAREPTRKIHGREPEFEVREPTRKIHGREIEAREPTRKIKP
	GAW0 8746.1	MRANFFIAGAMVIIFSILIACSRAAPIENLISRSGTGEASAADWKRSGTGEASAADWKRSGTGEASAADWRRSGTGEASAADWRRSGTGEAS AADWKRSGTGEASAADWRRSGTGEASAADWRRSGTNEESAADW
	GAW0 9164.1	MRSNFVLAGVMFIFSMLVSHLQAAPIELSERISERSGTNEGSAADWKRSGTSEGSAADWKRSGTNEGSAADWKRSGTNEGSAADW
	KAF88 32306. 1	MFSRTIICALMAFFLAGLATASPLPMPLQFARRGGLGPVDTIEVYRRENLVVRDHGIQYLPRQIPTENAVKSPAGTIVPYDKRQIPTEQAVKSPS GTIEPYKRTIPEEVAVKADTPGNIVQYRDVPSRARRATIPVESPVKALTPGDIVPY
	GAW0 3587.1	MPSRTTVYALLTLGLATLATASPLLFSDNVGGFYKRDIPEEQPVQQPGGVIIPYNKRQIIPVESPVQGPAPGHIEPYGRRQTPDEEPVKSLNGNIE PYGKRQTVPVEVPVRAPNGGIEPYSKRQIPAEQASSAPNGEIEPYGKRQIPDEQPVQAPNTGGIVPFKRTI
Pleurotus ostreatus, Umemura 2020	KDQ26 804.1	MVKSYSIALAATAILFAVAPAAAYEYHDAEARDLGDYSGHMARDLSSYYQFEAREPMDFEGDLYARDKKEEHKDHRKPFFKSRKGRKARKSRL AVLKKTCGKLENKDKDECMKQYKEVTDKTMAALNKYRPIVKKLKADKKKADEERKKKEAEKKKKDEEAKKKKEEEAKKKKEEDDKKKKEEEEK KKKEEEERKKKAGQKRDLDDLEDRDLDYESLLSIMQRAYDDSEDLYAREYLNLEELD
	KDQ26 999.1	MFSKQLFAFAILLGSTFAAALPVAEEGREAKRFFYTDYAVEVEKRDAEPEKRFFYTHYDAEVGKRDAEPEKRFFYTHYEAEMEKRSAEPDKRFFY TNYEAEVEKRDEEGTA
	KDQ28 763.1	MHFNKNFLIFVVAAVLTAANARPPLEVRRHGDSGSRGRGRSHSRSGIVARKHGDSGSRGRGRSHSNSGIIARKHGDSGSRGRGRSHSRSGLVA RKHGDSGSRGRGRSHSNSGIVARKHGDSGSRGRGRSRSTAGLVARKHGDSGSRGRGRSHSRSGLVARKHGDSGSRGRGRSHSRSGLVARKH GDSGSRGRGRSRSTSGLAARKHGDSGSRGRGRSRSRSGLVARKHGDSGSRGRGRSHSRSGLVARKHGDSGSRGRGRSHSRSGLVARAEEEAA QALVSSPASTVIRDLVGVVAATLVRGWGRGRSHSRSGLVARKHGDSGSRGRGRSHSRSGLVALAPVRASLLASMETPGPVDEAEATPALVWL DASMVILALEDVDVVAPDQALLPASMVTLALVDVGAPGLLELGK
	KDQ30 056.1	MVHTSTAVVFGALALNAASALATPLNIESNEPLETRAAKAGKSTLSRVGSGLGTAAAFAPIAGLGLSLATGQPLRRDAEGDDHIEARDSEDEDH LEARAGKSGLRRVGSALGTAAGFAPLAALGLGMATGQPVRRDFEEALYARNVEDNEHLEARGKKSKSGGKKSTLSRVGSGLGKAAAFAPLAAL

<table>
<tr><td></td><td></td><td>GLSLFGGQPVRRDLLEELLARDVDDDHLEARAVKSGLRRVGSGLGTAAAFAPLAALGLGMATGQPVRRELIEELLARDTDDDDHLQARATKSGLRRVGSALGTAAAFAPLAALGLGMATGQPVRRDLSAELMDIFHREISESLEDLD</td></tr>
<tr><td></td><td>KDQ32597.1</td><td>MKNSVVFMLFSAVALSGASQSVAEGSSRDSKPNIEARKAADYAPAAGMAAKILGQALNLPSAQNGPPTMGMGYGMGMGMGMGMGMGMGYYKREVEELLGRALSDDEAEALEDLHAREEEYEELETRKAADYAPAAGMAAKILGQALNLPSAQNGPPTMGMGYGMGMGMGTGYYRRDVVEELLGRELSDHEAGVMDYLRARDNEYEELEARKAADYAPAAGMAAKILGQALNLPSAQNGPPTMGMGYGMGMGMGMGYYRRDFEELLGRELSDDETRVLEGLQARDYDELEARDGADYASRQVEDLFERELLEMFDDSFI</td></tr>
<tr><td rowspan="14">Pleurotus ostreatus, Le Marquer et al., 2019</td><td>>jgi|PleosPC15_2|1070124</td><td>APIDAASLEARIPRPPSWKRGSPDWRRGSPDWRRGSPDWRRGGTQPDW</td></tr>
<tr><td>>jgi|PleosPC15_2|1077497</td><td>APVDQAAAREAPEKRFYYNNYDIEKRDASPEKRFYYTNYDVEKRGDAREKRFYYTHYDGKRFNYNKYKLEKRSEQPEKRFYYNNYEAQERNEQPAERRFYYNHYDMEKRGDEAVVVEERLMDDVPESVAA</td></tr>
<tr><td rowspan="4">>jgi|PleosPC15_2|1088560</td><td>GPISLVRPISERSPEPVDTIVAYAKRQIPAEVPTKSPAGTIVPYNKRQIPAEVPTKSPAG</td></tr>
<tr><td>TIVPYNKRQIPAEVPTKSPAGTIVPYSKRQIPAEVPTKSPAGTIVPYSKRQIPAEVPTKS</td></tr>
<tr><td>PAGTIVPYSKRQIPAEVATKAPNGQIVPYNKRQIPAEVPTKSPAGTIVPYSKRQIPAEVP</td></tr>
<tr><td>TKSPAGTIVPYSKRQIPAEVATKAPNGQIVPYNKRQIPAEVPTKSPAGTIEPYSN</td></tr>
<tr><td>>jgi|PleosPC15_2|1091723</td><td>APAGSPEVKRREAEARDTADMKREAYRPDWKREAMSGVAADWRREPEAMSGVAADWRRDPEAMSGVAADWRREPEAMSGVAADWRRDPEAMSGVAADWRRDPEAMSGVAADWRRDPEALSGVAADWRRDE</td></tr>
<tr><td>>jgi|PleosPC15_2|1099919</td><td>FPLEQGYDNDLEARGPTKRPSQQECEAACTLAGFQGQAWNQCVSQCVAGNYPAPPPSPQNRPVQQARPVPAPRIKVGKRGPTKRPTQQECEAACTLAGFQGQAWNQCVSQCVAGNYPTPPPSPQNRPVQQVKPAVRPAPRIRVGKRDDLEARGPTKRPTQQECEAACTLAGFQGQAWSQCVSQCVAGNYPTPPPSPQNRPAVRPAPRIKVGKRGPTKRPTQQECEAACTLAGFQGQAWNQCVSQCVAGNYPTPPPSPQNRPVQQARPAVRPAPRIKVGKRDDLEARGPTKRPTQQECEAACTLAGFQGQAWTQCVSQCVAGNYPTPPPSPQNRPVQQGKPMVRPAPMVKVGKREVADFGDDE</td></tr>
<tr><td>>jgi|PleosPC15_2|1103933</td><td>SSDNQATGSDALKPSYDTFAVRDIDYMSSIYAREPEPESDFWGHEARELDGDDDHNAPSDSSHNHSAREVEEDVHASGDNHHQRRAALDYVMDLAARSVDPDDHEGERLARREQLDRIVELAARYAPPQPPPNDNDHDKDNSERSLADDERLEARSALGMALRPFAKELGDVIKQVIANKMHRREEADDGREFDARDLHDAVHDELVARSAFGMAFRPIAQELGRAFRTAVLEKVKKREVEELEMRSAFGMAFRPIAQELGRAFRTAILEKVKREIDDREEADGVLESREDVVESALQS</td></tr>
<tr><td>>jgi|PleosPC15_2|1110971</td><td>TPMNVVDTTSHQARDVNENNSQLEERRIGSFLGKAAPFLPLLSFIPGLGGQQRRDVDELEARRLRDTLKNAAPWAPLVPLALDFFKSKPAQAPAEPAPIPPQRREIEEFLGRDLSDHEARGLGKFLGKAAPFIPFLSMIPGFGGQQRRDVNDLGVWGPLIPAGLHAANGGERMGPPPPQRREIEQMLERELTDVEARGLDGILDTVSQIAPWLPMIGGMLPESKPRPPKVPTKPAALRGKPKRSEEADESLEARRLRSTFDKVAPWGTVIGIGADLIRQKLSPKPAPAPVDPNAPAPAPVRRDTQEDNELEARATPKGAKSGKSTLSRIGSGLGTAAAFAPLIGLGIEAVNQGQMRRDIDEQFWARGVEDDEGLEARDFVEEFSARGVEDDDHLEARAPPKAGKSGKSTLSRIGSGLGTAAAFAPLIGLGLSALTQGQRRRDLEDQFWARDADDDDHLEARATPKGGKSTLSRIGSGLGTAAAFAPLVGLGLDAVTQGQMRRELYEEFFARDAEDELEERDFDDADDLVARDLEELLGRELTDIEARGFGTKLGKVAPYVPLIATGLSLFGGMGQARRDVSEELMDIFHRELSESLEDLD</td></tr>
<tr><td>>jgi|PleosPC15_2|1112933</td><td>NALPAVPTAPEVKGVAGNERSLNGNTFQTRDFDSRTDASVGSPPLAVPDVSVVPQAANSVFNDHTKKSHTGKDKMNKTRDIPTGPILEKKDTAATAEKQAGGYFPGGYGGGYGGYGGGYGGGYGGYRGYRFGGYKREEAATQAEQFGGFYGGYGRGYGRGRYGGYKREEAATAETQAEQASNYGYGGYGPYRGYIGYGGYGGGYGGYKREEELTKRGEAATQAEQFGGFYGGYGRGYGRGRYGGYKREEAATAETQAEQASNYGYGGGYGGGYGYGGYGGGYGGGYGYGGYGGYGHGGGYGGYKREEAATAETQAEQASNYGYGGGYGGGHGYGGYGGYGYGGRFGGYKREATKEKREEAATAETQAEQFGGYGGYGYGRGYRYGGYGGYKREEAATAETQAEQFGGYGGYGYGRGYRYGGYGGYKREEAATADTQAEQFGGYGYGRGFGRGRYGGYGSYKRDADETQNAQDADAADVETVAESETHTPETRSTPSIADLD</td></tr>
<tr><td>>jgi|PleosPC15_2|171464|</td><td>APVAQPDSSVIAARDGEFEARDMLLSREASPEANPRFRSPAWKREAEPGRWPPSWKREAEAEPEAWRADWKREAEAEPEAWRADWKREAEAAPEAWRADWKREAEAAPEPEAWRADWKRTPRPPAWKRDAAPSPPDW</td></tr>
<tr><td>>jgi|PleosPC15_2|171838|</td><td>VPKVTRDTATTDAVDADNNGWHGAENRREVATDAVDADNNGWHGAENRREIAAETDAVDSDNNGWHGAENRREVDTDAVDADNNGWHGAENRRDVAAETDAVDADNNGWHGAENKRELDADAVLILHSDADNNGWHGAENRRDIEADAVDADNNGWHGAENKRQVDADAVDADNNGWHGAENKREVDADAVDADNNGWHGAENRRDVDAVDPDNNGWHGRREVAAETDAVDADNNGWHGAENRR</td></tr>
<tr><td>>jgi|PleosPC15_2|171838|</td><td>VPKVTRDTATTDAVDADNNGWHGAENRREVATDAVDADNNGWHGAENRREIAAETDAVDSDNNGWHGAENRREVDTDAVDADNNGWHGAENRRDVAAETDAVDADNNGWHGAENKRELDADAVLILHSDADNNGWHGAENRRDIEADAVDADNNGWHGAENKRQVDADAVDADNNGWHGAENKREVDADAVDADNNGWHGAENRRDVDAVDPDNNGWHGRREVAAETDAVDADNNGWHGAENRR</td></tr>
</table>

Table S2: KEX2-processed repeat proteins (KEPs) in *L. edodes* and *P. ostreatus* detected by Le Marquer et al., 2019 and Umemura 2020.

The columns indicate the protein IDs (JGI (https://mycocosm.jgi.doe.gov) or NCBI (https://www.ncbi.nlm.nih.gov/)) and the protein sequence. KEX2 cleavage sites are labeled red and

potential STE13 recognition sequences green. The genome of *P. eryngii* was only published in 2021 and was therefore not included in the screens by Le Marquer et al., 2019 and Umemura 2020. Similarly, *L. edodes* was only analysed by Umemura 2020.

Strain label	Description	Strain number	Source
Pichia pastoris GS115	*P. pastoris* host strain for heterologous gene expression	SMA #2591	Invitrogen
P. pastoris GS115-405832	*P. pastoris* strain for heterologous expression of KEP 405832	SMA #3020	This study (L. Sonderegger)
P. pastoris GS115-434504	*P. pastoris* strain for heterologous expression of KEP 434504	SMA #3022	This study (L. Sonderegger)
P. pastoris GS115-490115	*P. pastoris* strain for heterologous expression of KEP 490115	SMA #3097	This study
P. pastoris GS115-497993	*P. pastoris* strain for heterologous expression of KEP 497993	SMA #3024	This study (L. Sonderegger)
P. pastoris GS115-503649	*P. pastoris* strain for heterologous expression of KEP 503649	SMA #3026	This study (L. Sonderegger)
P. pastoris GS115-426342	*P. pastoris* strain for heterologous expression of KEP 426342	SMA #3028	This study (L. Sonderegger)
P. pastoris GS115-empty	*P. pastoris* strain transformed with an empty vector, as negative control for heterologous expression.	SMA #1554	A. Essig, ETH Zürich
E. coli DH5α	Strain used for cloning and amplification of plasmids	SMA #2	Jeremy Thorner, University of California at Berkeley
E. coli BL21 DE3	Strain used for protein expression.	SMA #2600	Addgene
E.coli BL21_pET-NLS-Cas9-6xHis	Strain used for NLS-Cas9-6xHis expression.	SMA #3061	This study (L. Sonderegger)
B. subtilis 168	Strain used for bacterial toxicity tests with synthetic peptides	SMA #2598	Uwe Sauer, ETH Zürich
B. subtilis NCBI 3610	“	SMA #1279	Richard Losick, University of Harvard
Micrococcus luteus	“	Practical course database #72	DSMZ 20030
Staphylococcus aureus 113	“	SMA #1253	DSMZ 4910
Coprinopsis cinerea AmutBmut	A43mut B43mut *pab 1.2**	CMK #1	U. Kües, Georg-August-Universität Göttingen
C. cinerea AmutBmut pab1-2 Δku70	A43mut B43mut *pab 1.2 Δku70::*Flt^{R} *;Δku70* strain used for mutagenesis of *kex* genes	CMK #100	(10)
C. cinerea AmutBmut Δku70 Δkep 405832 #1	Knockout strain of KEP 405832 based on the parent strain *C. cinerea AmutBmut* pab1.2 *Δku70*	SMA #3212	This study
C. cinerea AmutBmut Δku70 Δkep 405832 #2	Knockout strain of KEP 405832 based on the parent strain *C. cinerea AmutBmut* pab1.2 *Δku70*	SMA #3213	This study
C. cinerea AmutBmut Δku70 Δkep 434504#1	Knockout strain of KEP 434504 based on the parent strain *C. cinerea AmutBmut* pab1.2 *Δku70*	SMA #3214	This study
C. cinerea AmutBmut Δku70 Δkep 434504#2	Knockout strain of KEP 434504 based on the parent strain *C. cinerea AmutBmut* pab1.2 *Δku70*	SMA #3215	This study
C. cinerea AmutBmut Δku70 Δkep 490115#1	Knockout strain of KEP 490115 based on the parent strain *C. cinerea AmutBmut* pab1.2 *Δku70*	SMA #3216	This study
C. cinerea AmutBmut Δku70 Δkep 490115#2	Knockout strain of KEP 490115 based on the parent strain *C. cinerea AmutBmut* pab1.2 *Δku70*	SMA #3217	This study
C. cinerea AmutBmut Δku70 Δkep 497993#1	Knockout strain of KEP 497993 based on the parent strain *C. cinerea AmutBmut* pab1.2 *Δku70*	SMA #3218	This study
C. cinerea AmutBmut Δku70 Δkep 497993#2	Knockout strain of KEP 497993 based on the parent strain *C. cinerea AmutBmut* pab1.2 *Δku70*	SMA #3219	This study
C. cinerea AmutBmut Δku70 Δkep 503649	Knockout strain of KEP 503649 based on the parent strain *C. cinerea AmutBmut* pab1.2 *Δku70*	SMA #3220	This study
C. cinerea AmutBmut Δku70 Δkep 426342#1	Knockout strain of KEP 426342 based on the parent strain *C. cinerea AmutBmut* pab1.2 *Δku70*	SMA #3221	This study
C. cinerea AmutBmut Δku70 Δkep 426342#2	Knockout strain of KEP 426342 based on the parent strain *C. cinerea AmutBmut* pab1.2 *Δku70*	SMA #3222	This study

C. cinerea AmutBmut Δku70 Δkex1 (437675)	Knockout strain of Kex protease based on the parent strain *C. cinerea AmutBmut* pab1.2 *Δku70*	SMA # 3317	This study
C. cinerea AmutBmut Δku70 Δkex2a (502579)	Knockout strain of Kex protease based on the parent strain *C. cinerea AmutBmut* pab1.2 *Δku70*	SMA #3095	This study (L. Sonderegger)
C. cinerea AmutBmut Δku70 Δkex2b (406374)	Knockout strain of Kex protease based on the parent strain *C. cinerea AmutBmut* pab1.2 *Δku70*	SMA #3211	This study (Y. Chen)
C. cinerea AmutBmut Δku70 Δkex2c (448165)	Knockout strain of Kex protease based on the parent strain *C. cinerea AmutBmut* pab1.2 *Δku70*	SMA #3318	This study
C. cinerea AmutBmut Δku70 Δkex2a/c (502579, 448165)	Double knockout strains of Kex proteases based on the parent strain *C. cinerea AmutBmut* pab1.2 *Δku70*	SMA #3319	This study

Table S3: Strains used in this study.

The *C. cinerea strain AmutBmut pab 1.2* is labeled according to Wälti et al. 2006. The same strain is labeled as *AmutBmut pab 1-1* in other publications.

**PCR/Sequencing primer**	**Sequence (5'-3')**	**Description**
Primer_f_KEP1(405832)	GTATACGCTATGCGCATCTC	Amplification of the KEP genes (with a small overhang into UTR (underlined) from the *C. cinerea* cDNA for heterologous expression in *P. pastoris*
Primer_r_ KEP1(405832)	CCGTCCATTATGCATTACC	"
Primer_f_ KEP2(434504)	TCAACATGGTTCAAATCTCC	"
Primer_r_ KEP2(434504)	CGTGCATCTAGTCGAGCT	"
Primer_f_KEP4(497993)	ATGTTCTCTCGCACTATCG	"
Primer_r_KEP4(497993)	TCACAAAGGTACGATTTCACC	"
Primer_f_KEP5(503649)	ATGATGCTCCGCACCAAC	"
Primer_r_KEP5(503649)	TCAAGCCTCAATTATATCCTCC	"
Primer_f_KEP6(426342)	TCAAGATGCAACTCCGTTTC	"
Primer_r_KEP6(426342)	CTATTTACAACAACTCCTCAACC	"
F_EcoRI_ApaI_KEP1(405832)	TAAGCAGAATTCATGCGCATCTCCGTTGTTTCT	Amplification of the KEPs from the pGEM-Teasy vectors for later restriction digest and insertion into pPICZA (EcoRI and ApaI restriction sites), with 5' overhang
R_EcoRI_ApaI_KEP1(405832)	TGCTTAGGGCCCCCACTTCACAGTCCCAGTCTG	"
F_EcoRI_ApaI_KEP2(434504)	TAAGCAGAATTCATGGTTCAAATCTCCACCTCC	"
R_EcoRI_ApaI_KEP2(434504)	TGCTTAGGGCCCGTCGAGCTCGTTGAGTTC	"
F_EcoRI_ApaI_KEP4(497993)	TAAGCAGAATTCATGTTCTCTCGCACTATC	"
R_EcoRI_ApaI_KEP4(497993)	TGCTTAGGGCCCCAAAGGTACGATTTCACC	"
F_EcoRI_ApaI_KEP5(503649)	TAAGCAGAATTCATGATGCTCCGCACCAACTTC	"
R_EcoRI_ApaI_KEP5(503649)	TGCTTAGGGCCCAGCCTCAATTATATCCTCCCAA	"
F_EcoRI_ApaI_KEP6(426342)	TAAGCAGAATTCATGCAACTCCGTTTCTCATTT	"
R_EcoRI_ApaI_KEP6(426342)	TGCTTAGGGCCCCAACAACTCCTCAACCTC	"
AOX1_f	GACTGGTTCCAATTGACA	Colony PCR to check for integration of genes into Pichia genome
AOX1_r	GCAAATGGCATTCTGACA	"
T7	5' TAATACGACTCACTATAGGG	Sequencing primers to confirm correct pGEM-Teasy plasmid construction
M13r	5' CAGGAAACAGCTATGAC	"
Pcpab1_seq_r	5' TCGGCATAGCGCAGCTAG	Sequencing primers to confirm correct gene mutation
Pcpab1_seq_f	5' TGAGTGGCGAAACAACTGTG	"
Snf5_seq_f	5' CTGGTTCCTCGACATCTGGC	"
Snf5_seq_r	5' CTCTCCACCAAGACCCAGATC	"
KEP1(405832)_seq_f	TTC AAC CAT CAG CTT CAT C	"
KEP1(405832)_seq_r	GTT ACG GAA TGA ACA CGA G	"
KEP2(434504)_seq_f	GAC ATT TAT TCC TGC TGT TCC	"
KEP2(434504)_seq_r	CAA CGG ATA AGG CAA AGT G	"
KEP3(490115)_seq_f	GTA TCC AAG GTG AAG GTG C	"

KEP3(490115)_seq_r	TAC CGA TAA AGG AGA CAA GGG	“
KEP4(497993)_seq_f	TCA TCT TGT TCT GGG TTT GC	“
KEP4(497993)_seq_r	GAC CTC TGT TTT GTC GAC C	“
KEP5(503649)_seq_f	ATT CAA TTC CGG TCC GAC	“
KEP5(503649)_seq_r	GTT TAC GGT GAC TCC TGG	“
KEP6(426342)_seq_f	CCA GGA GTC GAA GCT ATT ATC	“
KEP6(426342)_seq_r	GAA ATG CGG AGG GTG AAT C	“
KEX1_gene_r	AGTGTTTCTGGCCGTTCTCTT	Sequencing primers to confirm correct gene mutation, binding inside gene
KEX2c_gene_r	GACAGGCAGAGGAGCAGAAA	“
KEX2a_gene_r	CGATGAGCTCGAGAGGATG	“
KEX2b_gene_r	TCAGTGACCCGGTATGCAC	“
Cop6_seq_f	5' TTGCGCACCATAGGTCTTG	“
Cop6_seq_r	5' TCCACTAACAGGGTTTCTCC	“
KEX2a_seq_f	5' GGAGTTGCTGGTTGTTCAG	“
KEX2a_seq_r	5' GCACTTTCAATCATCGTAGCC	“
Kex1_probe_f	GAAGATAATCGCCAACGCC	“
Kex1_probe_r	GATTGTTGGAAATCCTAGAGTC	“
KEX2a _probe_f	5' TAATTGTCGGCTTCATCGTCC	Primers for amplification of labeled DNA probe for southern blots
KEX2a_probe_r	5' TACTGGTAACTGCACATGTCG	“
KEX2b_probe_f	GTCACACTGAGACCTTTGAACGC	“
KEX2b_probe_r	5'GTTCGAGCTTGCGACACGTC	“
KEX2c_probe_f	CGACGTTTATAGCAGGGTTGG	“
KEX2c_probe_r	GATCCCGTCACAGCCACTCA	“
KEP1(405832)_probe_f	CAAACGAAGGTGAGCAGG	“
KEP1(405832)_probe_r	TTTGGCTTTGGATGTGCG	“
KEP2(434504)_probe_f	AAGTCCAGGTTTCTTCCC	“
KEP2(434504)_probe_r	TTTGGCTTTGGATGTGCG	“
KEP3(490115)_probe_f	TTGTCTCGCACATGATGC	“
KEP3(490115)_probe_r	GGTAAGCGACAGATTTTGG	“
KEP4(497993)_probe_f	AATATCGTCGCCTCCAAG	“
KEP4(497993)_probe_r	CACAACATGATCGCCAGC	“
KEP5(503649)_probe_f	CATGGAGGCGTAATTTCTG	“
KEP5(503649)_probe_r	CATATGCTTGGAGGGTGG	“
KEP6(426342)_probe_f	GTTCTCTTTGATGCTGTG	“
KEP6(426342)_probe_r	AGCTAGGGAATGACAAC	“
KEP1(405832)_A_f	GCCAATGCCCTAAGTGGACTTGCGTG	Primers for cloning of repair templates for knockout establishment
KEP1(405832)_A_r	ggacctctgaattcttcttcAGCGTATACTCAGATCC	“
KEP1(405832)_B_f	GGATCTGAGTATACGCTgaagaagaattcagaggtcc	“
KEP1(405832)_B_r	GTCCGTCCATTATGCAcagtccacaatgacagc	“
KEP1(405832)_C_f	gctgtcattgtggactgTGCATAATGGACGGAC	“
KEP1(405832)_C_r	GCTCCTTTCACTATGTGAGCCGTATTTACTTAGTCC	“
KEP2(434504)_A_f	TGATGCAGCGATAAGTCAACACCC	“
KEP2(434504)_A_r	gcggacctctgaattcttcttcGTTGATAGATAGAAATTG	“
KEP2(434504)_B_f	CAATTTCTATCTATCAACgaagaagaattcagaggtccgc	“
KEP2(434504)_B_r	CTGCATATATCCCGTGCATcagtccacaatgacag	“
KEP2(434504)_C_f	ctgtcattgtggactgATGCACGGGATATATGCAG	“
KEP2(434504)_C_r	GTTGCTGAGCGAGGATCAGTACGG	“
KEP3(490115)_A_f	CACATAAATCGTTCGTGCTTGTCAAGGTCC	“
KEP3(490115)_A_r	cctctgaattcttcttcCGTGAGGACAACAGTGAG	“
KEP3(490115)_B_f	CTCACTGTTGTCCTCACGgaagaagaattcagagg	“
KEP3(490115)_B_r	GTTGGCGGTCGAAGGCGAcagtccacaatgacagc	“
KEP3(490115)_C_f	gctgtcattgtggactgTCGCCTTCGACCGCCAAC	“
KEP3(490115)_C_r	CATGAATAGACTATGTGGAAGAGAATTCGAAGTC GC	“

KEP4(497993)_A_f	CCTTCATCGTTGTCGCTGTCATCGCC	"
KEP4(497993)_A_r	gacctctgaattcttcttcCGTGGAGGAGGACGGG	"
KEP4(497993)_B_f	CCCGTCCTCCTCCACGgaagaagaattcagaggtc	"
KEP4(497993)_B_r	GAAAGCTCTCCAAGACTAAcagtccacaatgacagctctc	"
KEP4(497993)_C_f	gagagctgtcattgtggactgTTAGTCTTGGAGAGCTTTC	"
KEP4(497993)_C_r	GCTGAGTCCTCAGGTAGCGTTCGTAGG	"
KEP5(503649)_A_f	AGCCATGTGGTCAGTGTTCCCG	"
KEP5(503649)_A_f	gacctctgaattcttcttcCTTGTTGAGCAAAAAGT	"
KEP5(503649)_B_f	ACTTTTTGCTCAACAAGgaagaagaattcagaggtc	"
KEP5(503649)_B_f	GTAGTCAAAAGTCACGCcagtccacaatgacagctc	"
KEP5(503649)_C_f	gagctgtcattgtggactgGCGTGACTTTTGACTAC	"
KEP5(503649)_C_f	GAACAGTTGCGGTGAAACCAGAACCC	"
KEP6(426342)_A_f	GATGTACGTGCACTTCGGATGGGTG	"
KEP6(426342)_A_f	GCTTCACCTAAAGAATCAAGgaagaagaattcagaggtc c	"
KEP6(426342)_B_f	GCTTCACCTAAAGAATCAAGgaagaagaattcagaggtc c	"
KEP6(426342)_B_f	GGTCATCATGATCATCTATcagtccacaatgacagctctc	"
KEP6(426342)_C_f	gagagctgtcattgtggactgATAGATGATCATGATGACC	"
KEP6(426342)_C_f	CAAGTCCCACTGGTTCGTAGGGAACG	"
Kex1_A_f	ATGAGGATTTCCTCCATGACATTGACGGTATCGTT G	"
Kex1_A_r	GGACCTCTGAATTCTTCTTCTTCGGAAGCAGATTAC ATTAG	"
Kex1_B_f	CTAATGTAATCTGCTTCCGAAGAAGAAGAATTCAG AGGTCC	"
Kex1_B_r	GGCGATTATCTTCAGGTTATCAGTCCACAATGACA GCTC	"
Kex1_C_f	GAGCTGTCATTGTGGACTGATAACCTGAAGATAAT CGCC	"
Kex1_C_r	GTCGGGTGGATCGGTTACTAAACATGATTGTTGG	"
KEX2a_A_f	TTGAACCGTCCTCGAAGGTACTGGCTCGCCT	"
KEX2a_A_r	ggacctctgaattcttcttcTGTGATGGGGTATGGCGGGT	"
KEX2a_B_f	ACCCGCCATACCCCATCACAgaagaagaattcagaggtc c	"
KEX2a_B_r	CCCCAAACCCAAGAACCAATcagtccacaatgacagctct	"
KEX2a_C_f	agagctgtcattgtggactgATTGGTTCTTGGGTTTGGGG	"
KEX2a_C_r	ACCGCCTCGTCCCTCGCAATAGCAGCGGAGATAAA	"
KEX2b_A_f	GGACTTGGAGTGTGCGTCTTCCG	"
KEX2b_A_r	CCTCTGAATTCTTCTTCATGGACAAGAAGTGGTAG AA	"
KEX2b_B_f	TTCTACCACTTCTTGTCCATGAAGAAGAATTCAGA GGTCC	"
KEX2b_B_r	TCTCAGTGTGACTTACGCAGTCCACAATGACAGC	"
KEX2b_C_f	CTGTCATTGTGGACTGCGTAAGTCACACTGAGA	"
KEX2b_C_r	AGGGATTCGGTGATGGAGTATATGCCTC	"
KEX2c_A_f	CGGTCATCTCGCGGAGAATAAGGTGAT	"
KEX2c_A_r	CCTCTGAATTCTTCTTCAAATCGTCAATCGCCACGA T	"
KEX2c_B_f	GTGGCGATTGACGATTTGAAGAAGAATTCAGAGG	"
KEX2c_B_r	GCTTAGCTCTTGAGCCACAGTCCACAATGACAG	"
KEX2c_C_f	CTGTCATTGTGGACTGTGGCTCAAGAGCTAAGC	"
KEX2c_C_r	TGCGGTAAGGGTGGGGAAGTGTT	"

Table S4: Primers used in this study.

*kep*1<br>(405832)	ATGCGCATCTCCGTTGTTTCTGCCCTGACCCTCCTTGCGGCGACTAGTCTCACTTTCGCAGCTCCTACTA<br>CCTTTGGAGACCTCGTGAACAGGTCCGAGCGCCAAACTGGTGTCGTCAAAGGTCCAGAAAGGAGGAA<br>TATCATCGGGGTTGATGGAGATCTAGTTCAGCGGTCAGAACGGCAGACGGGCGTCGTAAAGCCACGA<br>AATGAGCCACCGGCCGACCGCGAAACCGGTACAGTGAAGTGGAAGCGACAGAATTCGGATCGCCAAA<br>CCGGGACTGTAAAGTGGAAGAGGCAGGACGACGATGAATCAGATCGCCAGACTGGGACTGTGAAGT<br>GGTAA	Coding sequence for heterologous expression in *P. pastoris*, stop codon in red
*kep*2<br>(434504)	ATGGTTCAAATCTCCACCTCCATCCTCGTCGCCGCCCTTGCCATCGCGCCCGTCCTCGCCGTCCCCATTG<br>CCCAGGAGGCCAACGAGTTCGTTGCCCGGGATGTTGATCTCGTAGCTGAGCCACTTGTCGCTCGAGAG<br>CCCATCTTTGGTTTCATCAAGAGGATTTTCACCGGGAAGCGAGACTTCTCTGAGTCTGAAGAGCTCGCT<br>CTCCGGGAGTTGGCCGAAGACCTTGACGCCCGTGAACCCATCTTCGGTTTCATCAAACGAATCTTCACC<br>GGCAAGCGGGACTTGTCCGAGTCTGAGGAGCTTGCCCTCCGTGAGCTTGTCGAAGACATCGACGCCCG<br>TGAGCCCATCTTCGGTTTCATCAAACGAATTTTCACTGGCAAGCGTGATCTTTCGGAGACCGAGGAGCT<br>CGCGCTCCGCGAGTACGTCGACTCGCTTGACGCCCGTGAACCTATTTTCGGGTTCATCAAACGTATCTT<br>CACCGGCAAGCGCGACCTTTCCGACCTCGACGACCTCTCCCTCCGCGACTTTGAGGACCTTGAAGCTCG<br>CGAGCCCATCTTCGGATTCATCAAGCGAATATTCACTGGCAAGCGTGATCTTTCCGAATCCGAGGAACT<br>CGCCCTCCGCGAGTATGTTGACTCCCTCGACGCCCGTGAGCCCATCTTTGGCTTCATCAAGAGAATCTTC<br>ACCGGCAAGCGAGACTTCTCCGACGTCGACGACCTTTCCCTTCGTGACTTCGAAGAGCTCGACGCCCGA<br>GAGCCCATCTTCGGTTTCATCAAGAGAATCTTCACTGGCAAGCGTGATCTCTCTGAGACTGAGGAGCTC<br>GCTCTTCGTGAGCTCGTCGACGACCTCGATGCCCGTGAACCCATCTTCGGCTTCATCAAGAGGATCTTC<br>ACCGGCAAGCGAGACCTCTCTGAATCCGAAGAGCTCGTCCTTCGCGAGTTCATGGACTCTCTCGACGCT<br>CGCGATCCTTCCTTTGCTGGCATTGCCAAGATCGGTGGCAAGGCCTTGAACTGGCTCGGTACCGCTGG<br>AACTCTCGCTTCAATCCCTGCTATGTTCAGGAGCAGCAAGAAGGACAAGCGCGACTTCGAGGACGACC<br>TCGTCTTCCGTGACTGGGTCCTCGAGGAGTTGGATGCCCGTGACTTTGACGATGACCTCGCCTACCGCA<br>TGTTCGACGATGAGTTCGACGCTCGTGAACTCAACGAGCTCGACTAG	“
*kep*3<br>(490115)	ATGCGGATTGCAAGTGCAGCCCTATTCTTCGTTTCGGTACTGAGTGTCGTTAGCGCCTTGCCTACACCT<br>GAGGTCGATCCCGTCGCCCCTGTGGCAGGAGTCGTTGCACGTGAACCTCAAATGAGGCCCCCTTCCTG<br>GAGAAGAGACCCGGAACCTCAAAGCAGGCCCCCTTCCTGGCGACGGGAGCCCGAACCCGAGCCCGAA<br>CCTCAATACAAAGCACCCTCATGGAGACGCGAACCCGAGCCTCAAAGCAGGCCTCCTGCCTGGCGCCG<br>CGAGCCTGAGCCCGAACCGCAATACAAGGCTCCTTCCTGGCGCCGCGAACCTGAGCCCGAACCGCAAT<br>ACAAGGCTCCTTCCTGGCGCCGCGAACCTGAGCCCGAACCCCAGTACAAAGCTCCTTCCTGGCGACGC<br>GATCCGGAACCTCAGTACAAGGCCCCTTCTTGGAGGCGCGAACCCGAACCCCAGATGAGGCCTCCCTC<br>CTGGTGA	“
*kep*4<br>(497993)	ATGTTCTCTCGCACTATCGTCTTCCTCACCCTCGCCCTCTCCCTGACTGGTCAGGGCCTAGCCGTCCCAG<br>TTGCCTCGAACATTGATGCCCGTCAGATCCCAGCTGAGCTGCCCACCAAGTCCATCAACGGCAACATTG<br>AACCATTCAAGGTTCGCCGTCAGGTTCCCGTGGACGAGCCTGCTGCTGCTCCCAATGGCATCGTGGTGC<br>CGTTCAACAAGCGTCAAATCCCTGCAGAGCTCCCAACCAAGTCGATCAACGGGAATGTCGAACCCTAC<br>AAGGTTCGCCGTCAAGTCCCTGTCGATGAGCCTGCTGCCGCTCCCAATGGAATCGTAGTGCCCTTCAAC<br>AAGCGCCAAATTCCCGCTGAGCTCCCGACCAAGTCCATTAACGGCAACATCGAGCCATACAAAGTTCGC<br>CGTCAAGTCCCTGTTGACGAGCCCGCTGCGGCACCCAATGGGATTGTGGTACCGTTCAACAAGCGTCA<br>AATCCCCGCTGAACTGCCAACCAAGTCGATCAACGGCAACATTGAGCCTTACAAGGTTCGCCGCCAGG<br>TCCCCGTGGACGAGCCTGCTGCCGCCCCCAATGGCATTGTAGTGCCCTTCAACAAGCGCCAAATCCCCG<br>CCGAACTGCCGACCAAGTCTATCAATGGCAACATCGAACCCTACAAGACCAAGCGGGGAGTGCGCTAC<br>GAAACTATCAGGTCCATTGGAGGCGATGAGATCATCGTCGAACTACGCGACGTCCCAAGCGAGATTGC<br>CGTCAGGTCCGTTGACGGTGAAATCGTACCTTTGTGA	“
*kep*5<br>(503649)	ATGATGCTCCGCACCAACTTCGTTCTCCTCGCCGTCGCCGCCTTCTCCACCCTCGGCTCGGTCTTCGCTG<br>CGCCTACTCGCACTTTCTCTGATGACTTTGACGCACGAGACCTCGCTTTTGAGGACATCCTCGACGCAC<br>GGGGTGGTCTCGCCATTCCTAAACCCACCCATGGAACCGACGGCGGAAAAGCCTCGTTGAACACCATT<br>GGAAAGGGCAGGCGCGCCTTCCTTGAAGACATCCTGGACATCCGTGGCGGCCTCGGAATCCCCAAGCC<br>TACTCACGGCACCGACGGTGGCAAAACCTCACTCAACACCATCGGCAAGGGAAGGCGTGCCCTCTTGG<br>AGGACTTCCTTGAGGCCAGGGGTGGACTCGCGCTTCCTAAGCCTACGCACGGCACCGACGGCGGCAA<br>GGCATCGCTGAACACCATCGGCAAGGGCAGGCGGGAGTTCTTGGAGGATCTCCTCGATGCTCGAGAC<br>CTGTTGGACGAGATTCTCGATGTGCGGGGTGGTTTGGCTCTCCCTAAACCTACACATGGCACGGATGG<br>TGGCAAGGCTTCCCTCAACACTATTGGCAAAGGCAGGAGGGATTTTTTCGAGGATATCCTTGATGCCA<br>GGGGCGGGCTCGCGCTCCCCAAGCCTACCCATGGAACTGACGGTGGCAAGGCTTCGATGAATACCATT<br>GGAAAGGGCAGGAGGGATTTTTGGGAGGATATAATTGAGGCTTGA	“
*kep*6<br>(426342)	ATGCAACTCCGTTTCTCATTTTTCGCTCTCGCCTTGTTGGCCCTCAACGCAGTCGCCACGCCCATCTCCTA<br>CGCCGACTCGGAGGACCTCGAAATCCGAGGCAAGAAGCCCAACCCCCTCCCAGCCGTCAACCAAAAGG<br>TCGCCAAACTGCCCGACGATCTCCGCCGCAAAGTCTTCGGTCACTGGAAGAAGCAGACTTTGCAACGT<br>GCCAACGCTCGGGTTGGACACAAGGCGATTATGCAGGAGTTGAAGGGACAGGCTCCTCCCGGCCGCT<br>GGACTGATCGCACTACAGCTGCTTGGGCTGTACACCAGAAGAACAAGAATCTGAGGAGGGAGTTGCT<br>GGACTCGGACGAGCTCGAAGTCCGAGGTAGGAAGCCCAACCCGCTGCCGCCTGTCAACCAAAAGGTC<br>GCCAAATTGCCTGAAGAACTCCGCCGCAAAGTCTTCGGTCACTGGAAGAAGCAGACTTTGCAGCGTGC<br>AAACGCTCGGGCTGGACACAAGGCGATCGTGCAGGAATTGAAGGGACAGGCTCCTCCCGGCCGCTGG<br>ACAGATCGAACTACCGCAGCTTGGGCTGTGCATGAGAAGAACAAGAATTTGAGGAGGGAGTTGGAAC<br>TTTTGGATGAGGTTGAGGAGTTGTTGTAA	“
*kep*3<br>(490115)<br>codon<br>optimized	GAATTCATGCGGATTGCAAGTGCAGCCCTATTCTTCGTTTCGGTACTGAGTGTCGTTAGCGCCTTGCCT<br>ACACCTGAGGTCGATCCCGTCGCCCCTGTGGCAGGAGTCGTTGCACGTGAACCTCAAATGAGGCCCCC<br>TTCCTGGAGAAGAGACCCGGAACCTCAAAGCAGGCCCCCTTCCTGGCGACGGGAGCCCGAACCCGAG<br>CCCGAACCTCAATACAAAGCACCCTCATGGAGACGCGAACCCGAGCCTCAAAGCAGGCCTCCTGCCTG<br>GCGCCGCGAGCCTGAACCTGAACCCCAGTATAAAGCCCCAAGCTGGCGTCGTGAACCAGAACCTGAGC<br>CACAGTATAAAGCACCCTCGTGGCGTCGAGAGCCAGAACCAGAGCCACAGTACAAAGCTCCTTCCTGG	Coding sequence of *kep* 490115, codon optimized, with restriction sites (underlined), without STOP codon. The digested piece can

	CGACGCGATCCGGAACCTCAGTACAAGGCCCCTTCTTGGAGGCGCGAACCCGAACCCCAGATGAGGCC TCCCTCCTGGGGGCCC	immediately be ligated into the pPICZA vector.

Table S5: Coding sequences of heterologously expressed *kep* genes.

Name	Description	Strain label	Source
pRS426-pAbGPDII-iePcGPD-Pcpab1-tPcMNP	Plasmid containing the *P. chrysosporium Pcpab1* cassette selection marker	PMK #476	(4)
pET-NLS-Cas9-6xHis	Plasmid for *S. pyogenes* Cas9 expression in bacterial cells	PMA #1582	Addgene (12)
pGEM-T easy_Snf5_Pcpab1	Repair-template plasmid for *snf5* disruption	PMA #1610	This study
pGEM-03563KO-PcPAB	Repair-template plasmid for *cop6* deletion	PMA #1184	(4)
pGEM-T easy_kex2a(502579)_Pcpab1	Repair-template plasmid for *kex* deletion	PMA #1628	This study
pGEM-Teasy_KO_kex2b(406374)_pab1	"	PMA #1658	This study
pGEM-Teasy_KO_kex2c(448165)_pab1	"	PMA #1657	This study
pGEM-Teasy_KO_kex1(437675)_pab1	"	PMA #1651	This study
pGEM-Teasy_KO_KEP1(405832)_pab1	Repair-template plasmid for *kep* deletion	PMA #1642	This study
pGEM-Teasy_KO_KEP2(434504)_pab1	"	PMA #1643	This study
pGEM-Teasy_KO_KEP3(490115) _pab1	"	PMA #1644	This study
pGEM-Teasy_KO_KEP4(497993)_pab1	"	PMA #1645	This study
pGEM-Teasy_KO_KEP5(503649)_pab1	"	PMA #1646	This study
pGEM-Teasy_KO_KEP6(426342)_pab1	"	PMA #1647	This study
pPICZA_KEP1(405832)	Plasmid for *P. pastoris* transformation	PMA #1574	This study
pPICZA_KEP2(434504)	"	PMA #1575	This study
pPICZA_KEP3(490115)	"	PMA #1613	This study
pPICZA_KEP4(497993)	"	PMA #1576	This study
pPICZA_KEP5(503649)	"	PMA #1577	This study
pPICZA_KEP6(426342)	"	PMA #1578	This study

Table S6: Plasmids used in this study.

snf5	ACUCAGCAUACCUCUAGAGCAGG
cop6_5'	GUUAUCACAAGGACGUCCUACGG
cop6_3'	UCCUCACCACGUUACAGGCUGGG
kex1_5'	UGAGCGGUCUCAGCGGGCUU
kex1_3'	AUCUUCAGGUUAUCGGAGCG
kex2a_5'	GCGGACGCUUCAAGGAAGGG
kex2a_3'	CUCUGCAACAAAAUCAAGUC
kex2b_5'	CGCUCAGGUUGUGGCGUUCA
kex2b_3'	GUCUCAGUGUGACUUACGCU
kex2c_5'	AUCGUGGCGAUUGACGAUUU
kex2c_3'	UUAUGUAUCUGUAAGACCGG
kep1_crRNA_5	GAGGGTCAGGGCAGAAACAA
kep1_crRNA_3	GCCAGACTGGGACTGTGAAG
kep2_crRNA_5	TCAATTTCTATCTATCAACA
kep2_crRNA_3	ACGAGCTCGACTAGATGCAC
kep3_crRNA_5	TCACTGTTGTCCTCACGATG
kep3_crRNA_3	GTCGAAGGCGATCACCAGGA

kep4_crRNA_5	GATAGTGCGAGAGAACATCG
kep4_crRNA_3	CTCTCCAAGACTAATCACAA
kep5_crRNA_5	CGAGGAGAACGAAGTTGGTG
kep5_crRNA_3	TTGACTACATATGTACTACA
kep6_crRNA_5	CGAGAGCGAAAAATGAGAAA
kep6_crRNA_3	TAGATGATCATGATGACCTG

Table S7: crRNAs used in this study.
These sequences consist of the protospacer sequence without the protospacer adjacent motif (PAM) and without the tracrRNA-complementary region.

Fungal strain	Reference genome
Coprinopsis cinerea AmutBmut pab1.2 v1.0	(1)
Coprinopsis cinerea Okayama 7 (#130)	(13)
Lentinula edodes W1-26	(14)
Pleurotus ostreatus PC15 v2.0	(15)
Pleurotus eryngii ATCC 90797 v1.0	(16)
Pichia pastoris GS115	(17)
Saccharomyces cerevisiae	(18)
Auriculariopsis ampla NL-1724 v1.0	(19)
Exidia glandulosa v1.0	(20)
Gloeophylum trabeum v1.0	(21)
Heliocybe sulcata OMC1185 v1.0	(22)
Armillaria gallica 21-2 v1.0	(23)
Psilocybe serbica v1.0	(24)
Coprinopsis marcescibilis CBS121175 v1.0	(22)
Amanita thiersii Skay4041 v1.0	(25)
Termitomyces sp. J132	(26)
Psathyrella aberdarensis	(27)
Hebeloma cylindrosporum h7 v2.0	(28)
Hypholoma sublateritium v1.0	(28)
Auricularia subglabra v2.0	(29)
Dendrothele bispora CBS 962.96 v1.0	(22)
Gymnopus luxurians v1.0	(28)
Panaeolus cyanescens	GenBank GCA_002938355.1
Laccaria bicolor S238N-H82]	(30)
Crucibulum laeve CBS 166.37 v1.0	(22)
Hypsizygus marmoreus 51987-8	(31)
Coprinus phaeopunctatus MPI-PUGE-AT-0042	(32)
Laccaria amethystina LaAM-08-1 v2.0	(28)
Cyathus striatus AH 40144 v1.0	(16)
Lyophyllum atratum CBS 144462 v1.0	(33)
Asterophora parasitica isolate AP01	NCBI accession number ASM1828200v1
Crassisporium funariophilum CBS 144457 v1.0	(33)
Coprinellus micaceus FP101781 v2.0	(22)
Auriculariales sp. MPI-PUGE-AT-0066	GenBank GCA_020744175.1
Gymnopus luxurians v1.0	(28)

Gymnopus confluens	(34)
Rhodocollybia butyracea AH 40177 v1.0	(16)
Neurospora crassa OR74A v2.0	(35)
Tricholoma furcatifolium	NCBI accession number ASM1885489v1
Blastosporella zonata	NCBI accession number ASM1885629v1
Guyanagaster necrorhizus MCA 3950 v1.0	(36)
Gautieria morchelliformis GMNE.BST v1.0	(37)
Pleurotus pulmonarius	NCBI accession number ASM1298053v1
Pleurotus cornucopiae	(38)
Cryptococcus amylolentus CBS 6039	(39)
Cryptococcus floricola	(40)
Cryptococcus wingfieldii CBS 7118	(40)
Cryptococcus neoformans var. grubii 125.91	NCBI accession number GCA_002215885
Cryptococcus gattii VGIV IND107	(41)
Cryptococcus depauperatus CBS 7841	NCBI accession number GCA_001720195.1
Kwoniella heveanensis BCC8398	GCA_000507405.3
Kwoniella dejecticola CBS 10117	GCA_000512565.2
Kwoniella pini CBS 10737	GCA_000512605.2
Kwoniella bestiolae CBS 10118	GCA_000512585.2
Kwoniella mangroviensis CBS 10435	GCA_000507885.2
Kwoniella shandongensis	GCA_008629635.1
Naematelia encephala	GCA_002105065.1
Saitozyma sp. JCM 24511	GCA_001600855.1
Cutaneotrichosporon oleaginosum	GCA_008065305.1
Apiotrichum porosum	GCA_003942205.1
Jaapia argillacea MUCL 33604	GCA_000697665.1
Piloderma croceum F 1598	(28)
Fibularhizoctonia sp. CBS 109695	(20)
Athelia sp. TMB	(42)
Abortiporus biennis	GCA_022606235.1
Polyporus brumalis	GCA_003367725.1
Polyporus arcularius HHB13444	GCA_004369055.1
Lentinus tigrinus ALCF2SS1-6	(43)
Earliella scabrosa	GCA_022605405.1
Hexagonia nitida	GCA_022606115.1
Ganoderma leucocontextum	GCA_022813035.1
Leiotrametes menziesii	GCA_022606275.1
Leiotrametes lactinea	GCA_022376465.1
Trametes coccinea BRFM310	GCA_002092935.1
Trametes elegans	GCA_022606155.1
Trametes cingulata	GCA_022385765.1
Trametes versicolor FP-101664 SS1	GCA_000271585.1
Trametes pubescens	GCA_001895945.1
Trametes polyzona	GCA_022606195.1
Lenzites betulinus	GCA_022264855.1
Taiwanofungus camphoratus	GCA_003999685.1
Amylocystis lapponica	GCA_022376435.1
Postia placenta MAD-698-R-SB12	(44)
Fibroporia radiculosa	GCA_000313525.1
Fomitopsis pinicola FP-58527 SS1	(29)

Punctularia strigosozonata HHB-11173 SS5	GCA_000264995.1
Multifurca ochricompacta	GCA_022496185.1
Scleroderma citrinum Foug A	(28)
Plicaturopsis crispa FD-325 SS-3	GCA_000827205.1
Galerina marginata CBS 339.88	GCA_023014335.1
Psilocybe cubensis	GCA_017499595.2
Panaeolus papilionaceus	GCA_015501605.1
Armillaria gallica	GCA_012064365.1
Mycena sanguinolenta	GCA_014462675.1
Lepista nuda	GCA_015584075.1
Tricholoma matsutake 945	(37)
Termitomyces sp. T112	GCA_018296085.1
Pluteus cervinus	GCA_004369065.1
Amanita brunnescens Koide BX004	GCA_001691785.2
Amanita rubescens	GCA_015039365.1
Vararia minispora EC-137	(45)
Serendipita vermifera MAFF 305830	(28)
Calocera viscosa TUFC12733	GCA_001630345.1
Calocera cornea HHB12733	GCA_001632435.1
Dacryopinax primogenitus	GCA_000292625.1
Rhodotorula sp. JG-1b	(46)
Rhodotorula sp. CCFEE 5036	(47)

Table S8: Reference genomes used in this study.

Coprinopsis cinerea		
**Gene name**	**JGI protein ID Okayama7** (13)	**JGI protein ID AmutBmut** (1)
kep	CC1G_04905	405832
kep	CC1G_06528	434504
kep	CC1G_11591	490115
kep	CC1G_10545	497993
kep	CC1G_06039	503649
kep	CC1G_09529	426342
kep	CC1G_06036	447393
Gene encoding glycoside hydrolase domain-containing protein	CC1G_01253	365456
kex1	CC1G_01392	437675
kex2a	CC1G_01625	502579
kex2b	CC1G_02379	406374
kex2c	CC1G_12012	448165
Ste13	CC1G_04021	545529
Glutaminyl cyclase (*qc*)	CC1G_13886	360529
snf5	CC1G_15539	365798
cop6	CC1G_03563	394772
Lentinula edodes W1-26		
	**JGI protein ID** (14)	
kep	2599	
Pleurotus ostreatus PC15 v2.0		

	**JGI protein ID** (15)
kep	1091723
Pleurotus eryngii ATCC 90797 v1.0	
	**JGI protein ID** (16)
kep	439342

Table S9: JGI protein IDs of proteins used in this study.

ANOVA results	Sum of Squares	dF	Mean Square	F (DFn, DFd)	P value
Treatment (between columns)	43.27	6	7.211	F (6, 13) = 25.00	P<0.0001
Residual (within columns)	3.75	13	0.2885		
Total	47.02	19			

Table S10: ANOVA results of mycelial diameter comparison between *C. cinerea* knockout strains.

Strain label	Sample origin	Description	Original file name
P. pastoris GS115-405832	SN	Heterologous expression of *C. cinerea* KEP *in P. pastoris*	20200506_006_S216402_2020_Pp_KEP1.raw
P. pastoris GS115-434504	SN	Heterologous expression of *C. cinerea* KEP *in P. pastoris*	20200506_002_S216403_2020_Pp_KEP2_rep.raw
P. pastoris GS115-490115	SN	Heterologous expression of *C. cinerea* KEP *in P. pastoris*	20200506_003_S216404_2020_Pp_KEP3.raw
P. pastoris GS115-497993	SN	Heterologous expression of *C. cinerea* KEP *in P. pastoris*	20200506_007_S216405_2020_Pp_KEP4.raw
P. pastoris GS115-503649	SN	Heterologous expression of *C. cinerea* KEP *in P. pastoris*	20200506_004_S216406_2020_Pp_KEP5.raw
P. pastoris GS115-426342	SN	Heterologous expression of *C. cinerea* KEP *in P. pastoris*	20200506_008_S216407_2020_Pp_KEP6.raw
P. pastoris GS115-empty	SN	*P. pastoris* strain transformed with an empty vector, as negative control for heterologous expression	20190808_004_S202141_DDA_Pp_empty.raw
C. cinerea AmutBmut pab1-2	SN	*C. cinerea* supernatant and tissue samples for screening for KEP-derived peptides	20200715_004_S219838_2020July_C_c__AmutBmut_HLB.raw
C. cinerea AmutBmut pab1-2 Δku70	SN	"	20211104_027_S325406_021121_ku70_SN_HLB_correctmethod.raw
"	Premature cap	"	20210818_009_S311407_170821_ku70_FB_HLB.raw
"	Cap	"	20211104_024_S325410_021121_ku70_overmature_HLB.raw
"	Mycelium	"	20211021_002_S322908_191021_ku70_drymyc1_MeOH37_HLB_20211022155916.raw
"	Basidio-spores	"	20210818_011_S311409_170821_ku70_basidiospore.raw
"	Stem	"	20211104_021_S325407_021121_ku70_stem_HLB.raw
C. cinerea AmutBmut Δku70 Δkep 405832 #1"	SN	Knockout strain of *C. cinerea* KEP	20220913_008_S412406_120922_CcKO_KEP1_SP3.raw
C. cinerea AmutBmut Δku70 Δkep 434504#1	SN	Knockout strain of *C. cinerea* KEP	20220905_028_S410966_020922_CcKO_KEP2_HLB.raw
C. cinerea AmutBmut Δku70 Δkep 490115#1	SN	Knockout strain of *C. cinerea* KEP	20210805_013_S310048_020821_31B_SP3.raw
C. cinerea AmutBmut Δku70 Δkep 497993#1	SN	Knockout strain of *C. cinerea* KEP	20220913_002_S310050_020821_44C_SP3.raw

C. cinerea AmutBmut Δku70 Δkep 497993#1	Cap	Knockout strain of *C. cinerea* KEP	20210916_021_S316137_150921_44C_FB.raw
C. cinerea AmutBmut Δku70 Δkep 503649"	SN	Knockout strain of *C. cinerea* KEP	20220913_009_S412407_120922_CcKO_KEP5_SP3.raw
C. cinerea AmutBmut Δku70 Δkep 426342#1	SN	Knockout strain of *C. cinerea* KEP	20220913_010_S412408_120922_CcKO_KEP6_SP3.raw
*C. cinerea AmutBmut Δku70 Δkex1 (*437675)	SN	Knockout strain of *C. cinerea* KEX protease	20220425_014_S368954_020322_kex1_2.raw
C. cinerea AmutBmut Δku70 Δkex2a (502579)	SN	Knockout strain of *C. cinerea* KEX protease	20220425_012_S368908_020322_kex2a_2.raw
"	Cap	Knockout strain of *C. cinerea* KEX protease	20220913_004_S316138_150921_kex2a_FB.raw
C. cinerea AmutBmut Δku70 Δkex2b (406374)	SN	Knockout strain of *C. cinerea* KEX protease	20220425_018_S382520_250422_kex2b-1.raw
*C. cinerea AmutBmut Δku70 Δkex2c (*448165)	SN	Knockout strain of *C. cinerea* KEX protease	20220425_022_S382523_250422_kex2c-6d-1.raw
*C. cinerea AmutBmut Δku70 Δkex2a/c (*502579, 448165)	SN	Double knockout strain of *C. cinerea* KEX proteases	20220425_026_S382526_250422_kex2ac-6d-1.raw
Lentinula edodes (strain 4312, Sylvan, USA)	Cap	Fruiting bodies purchased from a local mushroom farm	20210818_003_S311403_170821_shitake_fresh.raw
Pleurotus ostreatus (strain P24/HK35, Sylvan, USA)	Cap	Fruiting bodies purchased from a local mushroom farm	20210818_008_S311406_170821_pleurotus_dry.raw
Pleurotus ostreatus (strain P24/HK35, Sylvan, USA)	Cap	Fruiting bodies purchased from a local mushroom farm	20211021_005_S322911_191021_Pe_MeOH-37-HLB.raw
Pleurotus eryngii (strain 3066, Sylvan, USA)	Cap	Fruiting bodies purchased from a local mushroom farm	20220303_002_S316123_150921_Pe_MeOH-37-HLB.raw

Table S11: List of proteomics data deposited to the ProteomeXchange Consortium.
The data can be found online (http://proteomecentral.proteomexchange.org) with the dataset identifier PXD036934. The data was uploaded using the PRIDE partner repository (48).

Supplemental references

1. Muraguchi H, Umezawa K, Niikura M, Yoshida M, Kozaki T, Ishii K, Sakai K, Shimizu M, Nakahori K, Sakamoto Y, Choi C, Ngan CY, Lindquist E, Lipzen A, Tritt A, Haridas S, Barry K, Grigoriev I V., Pukkila PJ. 2015. Strand-specific RNA-seq analyses of fruiting body development in Coprinopsis cinerea. PLoS One 10:1–23.

2. Kombrink A, Tayyrov A, Essig A, Stöckli M, Micheller S, Hintze J, van Heuvel Y, Dürig N, Lin C wei, Kallio PT, Aebi M, Künzler M. 2019. Induction of antibacterial proteins and peptides in the coprophilous mushroom Coprinopsis cinerea in response to bacteria. ISME J 13:588–602.

3. Tayyrov A, Stanley CE, Azevedo S, Künzler M. 2019. Combining microfluidics and RNA-sequencing to assess the inducible defensome of a mushroom against nematodes. BMC Genomics 20:243.

4. Stöckli M, Lin C wei, Sieber R, Plaza DF, Ohm RA, Künzler M. 2017. Coprinopsis cinerea intracellular lactonases hydrolyze quorum sensing molecules of Gram-negative bacteria. Fungal Genet Biol 102:49–62.

5. Ando Y, Nakazawa T, Oka K, Nakahori K, Kamada T. 2013. Cc.snf5, a gene encoding a putative component of the SWI/SNF chromatin remodeling complex, is essential for sexual development in the agaricomycete Coprinopsis cinerea. Fungal Genet Biol 50:82–89.

6. Pearce M, Tivey ARN, Basutkar P, Lee J, Edbali O, Madhusoodanan N, Kolesnikov A, Lopez R. 2022. Search and sequence analysis tools services from EMBL-EBI in 2022 F abio 1–4.

7. Robert X, Gouet P. 2014. Deciphering key features in protein structures with the new ENDscript server. Nucleic Acids Res 42:320–324.

8. Petersen TN, Brunak S, Von Heijne G, Nielsen H. 2011. SignalP 4.0: Discriminating signal peptides from transmembrane regions. Nat Methods 8:785–786.

9. Swamy S, Uno I, Ishikawa T. 1984. Morphogenetic effects of mutations at the A and B incompatibility factors in Coprinus cinereus. J Gen Microbiol 130:3219–3224.

10. Nakazawa T, Honda Y. 2015. Absence of a gene encoding cytosine deaminase in the genome of the agaricomycete Coprinopsis cinerea enables simple marker recycling through 5-fluorocytosine counterselection. FEMS Microbiol Lett 362:1–7.

11. Wälti MA, Villalba C, Buser RM, Grünler A, Aebi M, Künzler M. 2006. Targeted gene silencing in the model mushroom Coprinopsis cinerea (Coprinus cinereus) by expression of homologous hairpin RNAs. Eukaryot Cell 5:732–744.

12. Zuris JA, Thompson DB, Shu Y, Guilinger JP, Bessen JL, Hu JH, Maeder ML, Joung JK, Chen ZY, Liu DR. 2015. Cationic lipid-mediated delivery of proteins enables efficient protein-based genome editing in vitro and in vivo. Nat Biotechnol 33:73–80.

13. Stajich JE, Wilke SK, Ahrén D, Au CH, Birren BW, Borodovsky M, Burns C, Canbäck B, Casselton LA, Cheng CK, Deng J, Dietrich FS, Fargo DC, Farman ML, Gathman AC, Goldberg J, Guigó R, Hoegger PJ, Hooker JB, Huggins A, James TY, Kamada T, Kilaru S, Kodira C, Kües U, Kupfer D, Kwan HS, Lomsadze A, Li W, Lilly WW, Ma LJ, Mackey AJ, Manning G, Martin F, Muraguchi H, Natvig DO, Palmerini H, Ramesh MA, Rehmeyer CJ, Roe BA, Shenoy N, Stanke M, Ter-Hovhannisyan V, Tunlid A, Velagapudi R, Vision TJ, Zeng Q, Zolan ME, Pukkila PJ. 2010. Insights into evolution of multicellular fungi from the assembled chromosomes of the mushroom Coprinopsis cinerea (Coprinus cinereus). Proc Natl Acad Sci U S A 107:11889–11894.

14. Chen L, Gong Y, Cai Y, Liu W, Zhou Y, Xiao Y, Xu Z, Liu Y, Lei X, Wang G, Guo M, Ma X, Bian Y. 2016. Genome sequence of the edible cultivated mushroom Lentinula edodes (shiitake)

reveals insights into lignocellulose degradation. PLoS One 11:1–20.

15. Riley R, Salamov AA, Brown DW, Nagy LG, Floudas D, Held BW, Levasseur A, Lombard V, Morin E, Otillar R, Lindquist EA, Sun H, LaButti KM, Schmutz J, Jabbour D, Luo H, Baker SE, Pisabarro AG, Walton JD, Blanchette RA, Henrissat B, Martin F, Cullen D, Hibbett DS, Grigoriev I V. 2014. Extensive sampling of basidiomycete genomes demonstrates inadequacy of the white-rot/brown-rot paradigm for wood decay fungi. Proc Natl Acad Sci U S A 111:9923–9928.

16. Ruiz-Dueñas FJ, Barrasa JM, Sánchez-García M, Camarero S, Miyauchi S, Serrano A, Linde D, Babiker R, Drula E, Ayuso-Fernández I, Pacheco R, Padilla G, Ferreira P, Barriuso J, Kellner H, Castanera R, Alfaro M, Ramírez L, Pisabarro AG, Riley R, Kuo A, Andreopoulos W, LaButti K, Pangilinan J, Tritt A, Lipzen A, He G, Yan M, Ng V, Grigoriev I V., Cullen D, Martin F, Rosso MN, Henrissat B, Hibbett D, Martínez AT. 2021. Genomic Analysis Enlightens Agaricales Lifestyle Evolution and Increasing Peroxidase Diversity. Mol Biol Evol 38:1428–1446.

17. De Schutter K, Lin YC, Tiels P, Van Hecke A, Glinka S, Weber-Lehmann J, Rouzé P, Van De Peer Y, Callewaert N. 2009. Genome sequence of the recombinant protein production host Pichia pastoris. Nat Biotechnol 27:561–566.

18. Goffeau A, Barrell G, Bussey H, Davis RW, Dujon B, Feldmann H, Galibert F, Hoheisel JD, Jacq C, Johnston M, Louis EJ, Mewes HW, Murakami Y, Philippsen P, Tettelin H, Oliver SG. 1996. Life with 6000 genes. Science (80-) 274:546–567.

19. Almási É, Sahu N, Krizsán K, Bálint B, Kovács GM, Kiss B, Cseklye J, Drula E, Henrissat B, Nagy I, Chovatia M, Adam C, LaButti K, Lipzen A, Riley R, Grigoriev I V., Nagy LG. 2019. Comparative genomics reveals unique wood-decay strategies and fruiting body development in the Schizophyllaceae. New Phytol 224:902–915.

20. Nagy LG, Riley R, Tritt A, Adam C, Daum C, Floudas D, Sun H, Yadav JS, Pangilinan J, Larsson KH, Matsuura K, Barry K, Labutti K, Kuo R, Ohm RA, Bhattacharya SS, Shirouzu T, Yoshinaga Y, Martin FM, Grigoriev I V., Hibbett DS. 2016. Comparative genomics of early-diverging mushroom-forming fungi provides insights into the origins of lignocellulose decay capabilities. Mol Biol Evol 33:959–970.

21. Kerem Z, Jensen KA, Hammel KE. 1999. Biodegradative mechanism of the brown rot basidiomycete Gloeophyllum trabeum: Evidence for an extracellular hydroquinone-driven fenton reaction. FEBS Lett 446:49–54.

22. Varga T, Krizsán K, Földi C, Dima B, Sánchez-García M, Sánchez-Ramírez S, Szöllősi GJ, Szarkándi JG, Papp V, Albert L, Andreopoulos W, Angelini C, Antonín V, Barry KW, Bougher NL, Buchanan P, Buyck B, Bense V, Catcheside P, Chovatia M, Cooper J, Dämon W, Desjardin D, Finy P, Geml J, Haridas S, Hughes K, Justo A, Karasiński D, Kautmanova I, Kiss B, Kocsubé S, Kotiranta H, LaButti KM, Lechner BE, Liimatainen K, Lipzen A, Lukács Z, Mihaltcheva S, Morgado LN, Niskanen T, Noordeloos ME, Ohm RA, Ortiz-Santana B, Ovrebo C, Rácz N, Riley R, Savchenko A, Shiryaev A, Soop K, Spirin V, Szebenyi C, Tomšovský M, Tulloss RE, Uehling J, Grigoriev I V., Vágvölgyi C, Papp T, Martin FM, Miettinen O, Hibbett DS, Nagy LG. 2019. Megaphylogeny resolves global patterns of mushroom evolution. Nat Ecol Evol 3:668–678.

23. Sipos G, Prasanna AN, Walter MC, O'Connor E, Bálint B, Krizsán K, Kiss B, Hess J, Varga T, Slot J, Riley R, Bóka B, Rigling D, Barry K, Lee J, Mihaltcheva S, Labutti K, Lipzen A, Waldron R, Moloney NM, Sperisen C, Kredics L, Vágvölgyi C, Patrignani A, Fitzpatrick D, Nagy I, Doyle S, Anderson JB, Grigoriev I V., Güldener U, Münsterkötter M, Nagy LG. 2017. Genome expansion and lineage-specific genetic innovations in the forest pathogenic fungi Armillaria. Nat Ecol Evol 1:1931–1941.

24. Fricke J, Blei F, Hoffmeister D. 2017. Enzymatic Synthesis of Psilocybin. Angew Chemie - Int Ed

56:12352–12355.

25. Hess J, Skrede I, Wolfe BE, Butti K La, Ohm RA, Grigoriev I V., Pringle A. 2014. Transposable element dynamics among asymbiotic and ectomycorrhizal amanita fungi. Genome Biol Evol 6:1564–1578.

26. Poulsen M, Hu H, Li C, Chen Z, Xu L, Otani S, Nygaard S, Nobre T, Klaubauf S, M. Schindler P, Hauser F, Pan H, Yang Z, Sonnenberg ASM, Wilhelm De Beer Z, Zhang Y, Wingfield MJ, Grimmelikhuijzen CJP, De Vries RP, Korb J, Aanen DK, Wang J, Boomsma JJ, Zhang G. 2014. Complementary symbiont contributions to plant decomposition in a fungus-farming termite. Proc Natl Acad Sci U S A 111:14500–14505.

27. Bau T, Yan JQ. 2021. Two new rare species of Candolleomyces with pale spores from China. MycoKeys 80:149–161.

28. Kohler A, Kuo A, Nagy LG, Morin E, Barry KW, Buscot F, Canbäck B, Choi C, Cichocki N, Clum A, Colpaert J, Copeland A, Costa MD, Doré J, Floudas D, Gay G, Girlanda M, Henrissat B, Herrmann S, Hess J, Högberg N, Johansson T, Khouja HR, Labutti K, Lahrmann U, Levasseur A, Lindquist EA, Lipzen A, Marmeisse R, Martino E, Murat C, Ngan CY, Nehls U, Plett JM, Pringle A, Ohm RA, Perotto S, Peter M, Riley R, Rineau F, Ruytinx J, Salamov A, Shah F, Sun H, Tarkka M, Tritt A, Veneault-Fourrey C, Zuccaro A, Tunlid A, Grigoriev I V., Hibbett DS, Martin F. 2015. Convergent losses of decay mechanisms and rapid turnover of symbiosis genes in mycorrhizal mutualists. Nat Genet 47:410–415.

29. Floudas D, Binder M, Riley R, Barry K, Blanchette RA, Henrissat B, Martínez AT, Otillar R, Spatafora JW, Yadav JS, Aerts A, Benoit I, Boyd A, Carlson A, Copeland A, Coutinho PM, De Vries RP, Ferreira P, Findley K, Foster B, Gaskell J, Glotzer D, Górecki P, Heitman J, Hesse C, Hori C, Igarashi K, Jurgens JA, Kallen N, Kersten P, Kohler A, Kües U, Kumar TKA, Kuo A, LaButti K, Larrondo LF, Lindquist E, Ling A, Lombard V, Lucas S, Lundell T, Martin R, McLaughlin DJ, Morgenstern I, Morin E, Murat C, Nagy LG, Nolan M, Ohm RA, Patyshakuliyeva A, Rokas A, Ruiz-Dueñas FJ, Sabat G, Salamov A, Samejima M, Schmutz J, Slot JC, John FS, Stenlid J, Sun H, Sun S, Syed K, Tsang A, Wiebenga A, Young D, Pisabarro A, Eastwood DC, Martin F, Cullen D, Grigoriev I V., Hibbett DS. 2012. The paleozoic origin of enzymatic lignin decomposition reconstructed from 31 fungal genomes. Science (80-) 336:1715–1719.

30. Martin F, Aerts A, Ahrén D, Brun A, Danchin EGJ, Duchaussoy F, Gibon J, Kohler A, Lindquist E, Pereda V, Salamov A, Shapiro HJ, Wuyts J, Blaudez D, Buée M, Brokstein P, Canbäck B, Cohen D, Courty PE, Coutinho PM, Delaruelle C, Detter JC, Deveau A, DiFazio S, Duplessis S, Fraissinet-Tachet L, Lucic E, Frey-Klett P, Fourrey C, Feussner I, Gay G, Grimwood J, Hoegger PJ, Jain P, Kilaru S, Labbé J, Lin YC, Legué V, Le Tacon F, Marmeisse R, Melayah D, Montanini B, Muratet M, Nehls U, Niculita-Hirzel H, Secq MPO Le, Peter M, Quesneville H, Rajashekar B, Reich M, Rouhier N, Schmutz J, Yin T, Chalot M, Henrissat B, Kües U, Lucas S, Van De Peer Y, Podila GK, Polle A, Pukkila PJ, Richardson PM, Rouzé P, Sanders IR, Stajich JE, Tunlid A, Tuskan G, Grigoriev I V. 2008. The genome of Laccaria bicolor provides insights into mycorrhizal symbiosis. Nature 452:88–92.

31. Min B, Kim S, Oh YL, Kong WS, Park H, Cho H, Jang KY, Kim JG, Choi IG. 2018. Genomic discovery of the hypsin gene and biosynthetic pathways for terpenoids in Hypsizygus marmoreus. BMC Genomics 19:1–12.

32. Mesny F, Miyauchi S, Thiergart T, Pickel B, Atanasova L, Karlsson M, Hüttel B, Barry KW, Haridas S, Chen C, Bauer D, Andreopoulos W, Pangilinan J, LaButti K, Riley R, Lipzen A, Clum A, Drula E, Henrissat B, Kohler A, Grigoriev I V., Martin FM, Hacquard S. 2021. Genetic determinants of endophytism in the Arabidopsis root mycobiome. Nat Commun 12:1–15.

33. Steindorff AS, Carver A, Calhoun S, Stillman K, Liu H, Lipzen A, He G, Yan M, Pangilinan J,

LaButti K, Ng V, Bruns TD, Grigoriev I V. 2021. Comparative genomics of pyrophilous fungi reveals a link between fire events and developmental genes. Environ Microbiol 23:99–109.

34. Antonín V, Halling RE, Noordeloos ME. 1997. Generic concepts within the groups of Marasmius and Collybia sensu lato. Mycotaxon 63:359–368.

35. Galagan JE, Calvo SE, Borkovich KA, Selker EU, Read NO, Jaffe D, FitzHugh W, Ma LJ, Smirnov S, Purcell S, Rehman B, Elkins T, Engels R, Wang S, Nielsen CB, Butler J, Endrizzi M, Qui D, Ianakiev P, Bell-Pedersen D, Nelson MA, Werner-Washburne M, Selitrennikoff CP, Kinsey JA, Braun EL, Zelter A, Schulte U, Kothe GO, Jedd G, Mewes W, Staben C, Marcotte E, Greenberg D, Roy A, Foley K, Naylor J, Stange-Thomann N, Barrett R, Gnerre S, Kamal M, Kamvysselis M, Mauceli E, Bielke C, Rudd S, Frishman D, Krystofova S, Rasmussen C, Metzenberg RL, Perkins DD, Kroken S, Cogoni C, Macino G, Catcheside D, Li W, Pratt RJ, Osmani SA, DeSouza CPC, Glass L, Orbach MJ, Berglund JA, Voelker R, Yarden O, Plamann M, Seiler S, Dunlap J, Radford A, Aramayo R, Natvig DO, Alex LA, Mannhaupt G, Ebbole DJ, Freitag M, Paulsen I, Sachs MS, Lander ES, Nusbaum C, Birren B. 2003. The genome sequence of the filamentous fungus Neurospora crassa. Nature 422:859–868.

36. Koch RA, Yoon GM, Aryal UK, Lail K, Amirebrahimi M, LaButti K, Lipzen A, Riley R, Barry K, Henrissat B, Grigoriev I V., Herr JR, Aime MC. 2021. Symbiotic nitrogen fixation in the reproductive structures of a basidiomycete fungus. Curr Biol 31:3905-3914.e6.

37. Miyauchi S, Kiss E, Kuo A, Drula E, Kohler A, Sánchez-García M, Morin E, Andreopoulos B, Barry KW, Bonito G, Buée M, Carver A, Chen C, Cichocki N, Clum A, Culley D, Crous PW, Fauchery L, Girlanda M, Hayes RD, Kéri Z, LaButti K, Lipzen A, Lombard V, Magnuson J, Maillard F, Murat C, Nolan M, Ohm RA, Pangilinan J, Pereira M de F, Perotto S, Peter M, Pfister S, Riley R, Sitrit Y, Stielow JB, Szöllősi G, Žifčáková L, Štursová M, Spatafora JW, Tedersoo L, Vaario LM, Yamada A, Yan M, Wang P, Xu J, Bruns T, Baldrian P, Vilgalys R, Dunand C, Henrissat B, Grigoriev I V., Hibbett D, Nagy LG, Martin FM. 2020. Large-scale genome sequencing of mycorrhizal fungi provides insights into the early evolution of symbiotic traits. Nat Commun 11:1–17.

38. Zhang Y, Gao W, Sonnenberg A, Chen Q, Zhang J, Huang C. 2021. Genetic Linkage and Physical Mapping for an Oyster Mushroom (Pleurotus cornucopiae) and Quantitative Trait Locus Analysis for Cap Color. Appl Environ Microbiol 87:e0095321.

39. Sun S, Yadav V, Billmyre RB, Cuomo CA, Nowrousian M, Wang L, Souciet JL, Boekhout T, Porcel B, Wincker P, Granek JA, Sanyal K, Heitman J. 2017. Fungal genome and mating system transitions facilitated by chromosomal translocations involving intercentromeric recombination. PLoS Biol 15:e2002527.

40. Passer AR, Coelho MA, Billmyre RB, Nowrousian M, Mittelbach M, Yurkov AM, Averette AF, Cuomo CA, Sun S, Heitman J. 2019. Genetic and genomic analyses reveal boundaries between species closely related to Cryptococcus pathogens. MBio 10:e00764-19.

41. Farrer RA, Desjardins CA, Sakthikumar S, Gujja S, Saif S, Zeng Q, Chen Y, Voelz K, Heitman J, May RC, Fisher MC, Cuomo CA. 2015. Genome evolution and innovation across the four major lineages of cryptococcus gattii. MBio 6:e00868-15.

42. Konkel Z, Scott K, Slot JC. 2021. Draft Genome Sequence of the Termite-Associated "Cuckoo Fungus," Athelia (Fibularhizoctonia) sp. TMB Strain TB5. Microbiol Resour Announc 10:28–30.

43. Wu B, Xu Z, Knudson A, Carlson A, Chen N, Kovaka S, LaButti K, Lipzen A, Pennachio C, Riley R, Schakwitz W, Umezawa K, Ohm RA, Grigoriev I V., Nagy LG, Gibbons J, Hibbett D. 2018. Genomics and development of Lentinus tigrinus: A white-rot wood-decaying mushroom with

dimorphic fruiting bodies. Genome Biol Evol 10:3250–3261.

44. Martinez D, Challacombe J, Morgenstern I, Hibbett D, Schmoll M, Kubicek CP, Ferreira P, Ruiz-Duenas FJ, Martinez AT, Kersten P, Hammel KE, Vanden Wymelenberg A, Gaskell J, Lindquist E, Sabat G, BonDurant SS, Larrondo LF, Canessa P, Vicuna R, Yadav J, Doddapaneni H, Subramanian V, Pisabarro AG, Lavín JL, Oguiza JA, Master E, Henrissat B, Coutinho PM, Harris P, Magnuson JK, Baker SE, Bruno K, Kenealy W, Hoegger PJ, Kües U, Ramaiya P, Lucas S, Salamov A, Shapiro H, Tu H, Chee CL, Misra M, Xie G, Teter S, Yaver D, James T, Mokrejs M, Pospisek M, Grigoriev I V., Brettin T, Rokhsar D, Berka R, Cullen D. 2009. Genome, transcriptome, and secretome analysis of wood decay fungus Postia placenta supports unique mechanisms of lignocellulose conversion. Proc Natl Acad Sci U S A 106:1954–1959.

45. Looney B, Miyauchi S, Morin E, Drula E, Courty PE, Kohler A, Kuo A, LaButti K, Pangilinan J, Lipzen A, Riley R, Andreopoulos W, He G, Johnson J, Nolan M, Tritt A, Barry KW, Grigoriev I V., Nagy LG, Hibbett D, Henrissat B, Matheny PB, Labbé J, Martin FM. 2022. Evolutionary transition to the ectomycorrhizal habit in the genomes of a hyperdiverse lineage of mushroom-forming fungi. New Phytol 233:2294–2309.

46. Goordial J, Raymond-Bouchard I, Riley R, Ronholm J, Shapiro N, Woyke T, LaButti KM, Tice H, Amirebrahimi M, Grigoriev I V., Greer C, Bakermans C, Whyte L. 2016. Improved high-quality draft genome sequence of the eurypsychrophile Rhodotorula sp. JG1b, isolated from permafrost in the hyperarid upper-elevation McMurdo Dry Valleys, Antarctica. Genome Announc 4:9–10.

47. Coleine C, Masonjones S, Onofri S, Selbmann L, Stajich JE. 2020. Draft Genome Sequence of the Yeast Rhodotorula sp. Strain CCFEE 5036, Isolated from McMurdo Dry Valleys, Antarctica 9:14–16.

48. Perez-Riverol Y, Csordas A, Bai J, Bernal-Llinares M, Hewapathirana S, Kundu DJ, Inuganti A, Griss J, Mayer G, Eisenacher M, Pérez E, Uszkoreit J, Pfeuffer J, Sachsenberg T, Yilmaz Ş, Tiwary S, Cox J, Audain E, Walzer M, Jarnuczak AF, Ternent T, Brazma A, Vizcaíno JA. 2019. The PRIDE database and related tools and resources in 2019: Improving support for quantification data. Nucleic Acids Res 47:D442–D450.
